# Discrete subdomains establish epigenetic diversity in subtelomeric heterochromatin in fission yeast

**DOI:** 10.1038/s41467-026-78445-5

**Published:** 2026-10-12

**Authors:** Agnisrota Mazumder, John Cooper, Can Goksal, Rosario Y. J. Brockhausen, Jonas M. Schauß, Jasbeer S. Khanduja, Richard I. Joh, Ahmed A. A. Amine, Johannes J. Groos, Junko Kanoh, Mo Motamedi, Ilya J. Finkelstein, Bassem Al-Sady, Sigurd Braun

**Affiliations:** 1 Institute of Genetics, Faculty of Biology and Chemistry, Justus Liebig University Giessen, Giessen, Germany; 2 BioMedical Center (BMC), Division of Physiological Chemistry, Faculty of Medicine, LMU Munich, Munich, Germany; 3 Dept. Molecular Biosciences, The University of Texas-Austin, Austin, TX USA; 4 Department of Microbiology and Immunology, George Williams Hooper Foundation, University of California San Francisco, San Francisco, CA USA; 5 TETRAD graduate program, University of California San Francisco, San Francisco, CA USA; 6 Mass General Brigham Krantz Family Center for Cancer Research and Department of Medicine, Harvard Medical School, Charlestown, MA USA; 7 Department of Physics, Virginia Commonwealth University, Richmond, VA USA; 8 Dept. Life Sciences, Graduate School of Arts and Sciences, The University of Tokyo, Tokyo, Japan; 9 Present Address: EMBL Partnership Institute, Vilnius University, Vilnius, Lithuania

**Keywords:** Gene silencing, Telomeres, Chromatin structure

## Abstract

Subtelomeres are imperfect repeats adjacent to telomeres that are transcriptionally repressed by heterochromatin. Although essential for genome integrity, their repetitive nature has thwarted dissection of local heterochromatin assembly and maintenance mechanisms. By engineering fission yeast strains carrying fluorescent reporters at a single subtelomere, we uncovered distinct subdomains. These subdomains have different silencing requirements: telomere-proximal regions rely on canonical shelterin- or RNAi-dependent nucleation pathways, whereas telomere-distal regions involve nucleosome remodelers, histone chaperones, and boundary-associated factors. Subdomains also exhibit discrete epigenetic states emerging both at homologous loci on different chromosome arms and along the same subtelomeric sequence. We document these epigenetic states using multi-generational live imaging and targeted perturbations. These analyses show that subtelomeric subdomains display position-specific, clonally variable silencing across a spectrum from robust to fragile epigenetic states. Interestingly, structural variants, common in subtelomeric sequences across eukaryotes, dictate local epigenetic stability. These findings reveal that subtelomeres, long recognized for their sequence variability, form a dynamic mosaic of coexisting epigenetic domains. This implies a wide range of gene-repressive regulatory logic at the chromosome ends, from environmental responsiveness to silencing stability.

## Abstract (web summary)

Here the authors show that subtelomeric heterochromatin in S. pombe forms a mosaic of subdomains with distinct requirements for gene silencing and different epigenetic stability, ranging from highly fragile to very stable.

## Introduction

Gene-repressing heterochromatin is a highly conserved feature marked by methylation of histone H3 at lysine 9 (H3K9me). This histone modification is deposited by Suv39-family methyltransferases, such as Suv39h1 and Suv39h2 in mammalian systems or Clr4 in the fission yeast *Schizosaccharomyces pombe* (*S. pombe*)^1^. Heterochromatin formation is initiated when Suv39 methyltransferases are guided to specific sites by non-coding RNAs or DNA-bound factors. Once nucleated by this mechanism, heterochromatin expands into adjacent regions via a DNA sequence-independent mechanism termed spreading. This expansion depends on conserved positive feedback involving Suv39, its enzymatic product, and reader proteins recognizing H3K9me, including members of the Heterochromatin Protein 1 (HP1) family^2–5^. Local three-dimensional (3D) contacts between nucleosomes are critical for this positive feedback across systems, facilitating both spreading and maintenance of the heterochromatic domains^6,7^. In both yeast and metazoans, such mechanisms typically support only short-range spreading, rarely exceeding 10 kb^6,8–10^. In *S. pombe*, this constraint is evident when heterochromatin nucleates and spreads within a euchromatic region, either through ectopic heterochromatin assembly^11–14^ or at native heterochromatin islands^15^. However, assessing spreading in constitutive heterochromatin is more complex, as these domains often contain multiple distinct nucleation sites^16–18^ and are insulated by defined DNA elements in yeast and mammals (CTCF)^19^. Consequently, it remains unclear whether spreading in these regions is limited or whether it can propagate more extensively than in euchromatic environments.

Such long-range spreading is best assessed in simple model systems with defined and minimally redundant heterochromatin pathways. *S. pombe* has been an outstanding model system for dissecting mechanisms of constitutive heterochromatin formation and addressing these questions. This is due to its limited and spatially defined heterochromatin content, making up only 2% of its genome, the low redundancy of trans acting factors, and its facile genetics. Much progress has been made in uncovering mechanisms of heterochromatin formation at domains with defined nucleation sites, in particular the MAT locus and the pericentromeres. However, the subtelomeric regions, which present the most conserved and largest heterochromatin domains extending over 50 kb^19–21^, have been proven more difficult to investigate. Subtelomeres lie adjacent to telomeres but are structurally distinct, consisting of mosaic-like arrays of common sequence elements that occur in variable copy numbers rather than the short tandem repeats present at telomeric regions. This structural complexity, combined with their repeated occurrence across chromosomes, has hindered efforts to precisely define local heterochromatin spreading dynamics. Although subtelomeres are hotspots of genome evolution, subtelomeric heterochromatin plays a protective role by suppressing excessive recombination and restricting the expression of transcripts whose misregulation has been linked to disease^22,23^. Despite this important function, the rules governing subtelomeric heterochromatin formation, dynamics, and epigenetic stability remain poorly understood. In *S. pombe*, as in other systems, subtelomeres lack known internal nucleation sites and instead rely on heterochromatin nucleation near chromosome ends, from which H3K9me-marked domains are thought to extend. Although the molecular organization of telomeres varies across model systems, heterochromatin nucleation at chromosomal ends, either within telomere repeats or adjacent subtelomeric regions, appears to be evolutionarily conserved across fungi, plants, and animals^24–26^.

In *S. pombe*, subtelomeric heterochromatin assembles at the subtelomeric homologous (SH) sequences, which are present on most chromosomal arms. Ranging from 33 to 55 kb, the SH sequences are composed of imperfect repeats and multiple gene-containing segments that share ~90% identity between chromosomal arms, with occasional insertions and deletions^22,27,28^. SH regions lack DNA-encoded boundary elements and instead exhibit gradual transitions into adjacent subtelomeric unique (SU) regions, which are devoid of heterochromatic marks^21,28^. Two distinct nucleation pathways initiate heterochromatin formation near the telomeric repeats through the recruitment of Clr4. The first relies on RNA interference (RNAi) and nucleates at repeats present in *tlh1–4* genes, ancient duplications of RecQ helicase-derived genes^29^. These repeats drive RNAi-mediated nucleation at pericentromeres, the MAT locus, and subtelomeres via the RNA-induced transcriptional silencing (RITS) complex, in which the scaffold protein Tas3 bridges Argonaute (Ago1) and the chromodomain protein Chp1^19,20,27,29^. The second pathway depends on the telomere-protecting shelterin complex, which is targeted to telomere-proximal sequences by Taz1 and recruits Clr4 and the Snf2-like remodeler/HDAC-containing repressor complex (SHREC) via the shelterin subunit Ccq1^20,30–35^. In addition, the inner nuclear membrane protein Lem2 facilitates SHREC recruitment and acts redundantly with shelterin and RNAi^36–38^.

The relatively large size and the absence of known boundary elements raise key questions about how such extended heterochromatin is assembled and maintained. In one model, SH may form a single continuous domain. Here, RNAi- and shelterin-dependent pathways seed heterochromatin at the telomere-proximal end, resulting in its propagation toward the SH/SU boundary. This process potentially requires mechanisms that exclude heterochromatin antagonists such as Epe1^39^. Alternatively, multiple independent nucleation events may occur along the SH region, generating distinct subdomains. To overcome the challenges of SH sequence similarity within and across chromosome arms^27^, we integrated a series of gene silencing reporters into a previously generated yeast strain, which only contains a single SH domain^21^. This approach enabled the detailed dissection of the molecular characteristics, epigenetic behavior, and genetic requirements governing SH heterochromatin. We find evidence for multiple, epigenetically distinct subdomains within the SH region that initiate heterochromatin formation independently of previously described, canonical molecular mechanisms.

## Results

### The SH domain carries a heterogeneous chromatin landscape

**Fig. 1:**
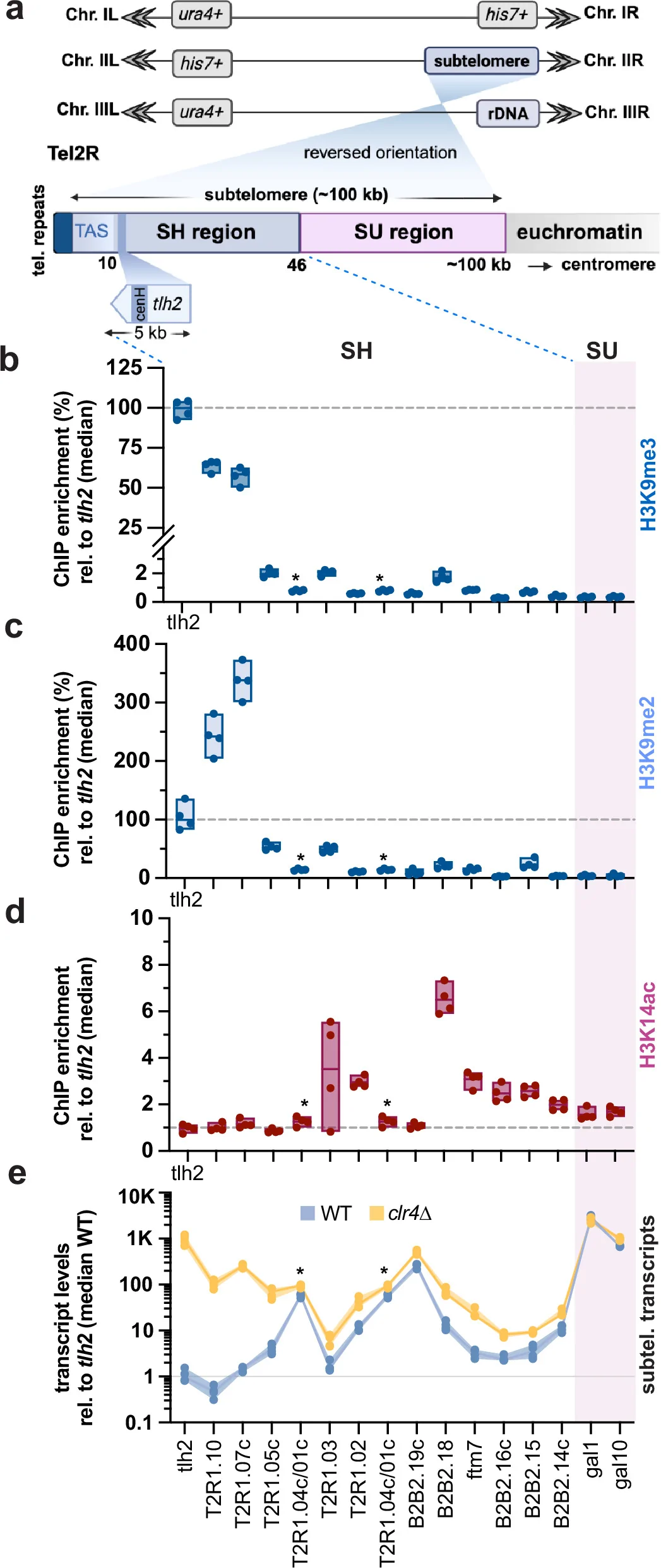
The SH domain carries a heterogeneous chromatin landscape. **a** Schematic of the *SD4[2R*+*]* strain with engineered subtelomeric deletions. The 100-kb subtelomeric domain comprises a ~50-kb subtelomeric homology (SH, blue) region and a subtelomeric unique (SU, pink) region. All other SH sequences were replaced with *ura4* or *his7* markers, leaving only the native subtelomere on the right arm of chromosome II. Following previous studies (Oizumi et al.^27^), Tel2R is shown in reversed orientation (telomere left, centromere right) to facilitate comparison across subtelomeres; this orientation is used throughout this study. Created in BioRender. Braun, S. (2026) https://BioRender.com/s50mjk7. ChIP-qPCR analysis of histone modifications across SH region and part of SU: **b** H3K9me3, **c** H3K9me2 and **d** H3K14ac. Input-normalized IP signals were normalized to the mean across subtelomeric loci (see Methods and Source Data) and are shown relative to median *tlh2*. Floating bars show the median and range (minimum to maximum); individual data points representing four independent biological replicates (*n* = 4). The SU region is shaded in pink. Primer pairs for unique target sequences are described in Lei et al. 2020 (ref. ^86^). **e** RT-qPCR analysis of SH and SU gene expression in wild-type (WT; blue) and *clr4Δ* (yellow) strains. Transcript levels were normalized to *act1* and are shown relative to the median *tlh2* expression in WT. Independent biological replicates (*n* = 4) are shown as individual data points, with the median and range. SU is shaded in pink. Asterisks in (**b**–**d**) denote gene pair *SPBCPT2R1.04c*/*SPBCPT2R1.01c*, which cannot be distinguished due to 99.9% sequence identity.

To determine whether the ~50 kb heterochromatin domain assembled over SH sequences is contiguous or comprises discrete domains with potentially independent nucleation sites, we analyzed a fission yeast strain in which all but one SH domain was replaced by marker genes (*his7*, *ura4*)^21^. Specifically, we used the *SD4[2R*+] strain that carries a single SH domain on the right arm of chromosome II, while the remaining SH sequences have been removed (Fig. 1a). Heterochromatin dosage overall is not altered in this strain, as SH-independent heterochromatin of comparable size is still formed on the other chromosome ends^21^. Chromatin immunoprecipitation followed by quantitative PCR (ChIP-qPCR) across individual subtelomeric genes revealed strong enrichment of tri- and di-methylated H3K9 (H3K9me3, H3K9me2) within a ~10-kb region near the telomere (Fig. 1b, c). This region includes the RNAi-dependent nucleation site at *tlh2*, which shares sequence similarity with pericentromeric *dg-dh* repeats and the *cenH* element at the mating type locus^20^. We found that H3K9me3 levels peak near the *tlh2* nucleation site, while H3K9me2 reaches maximum levels more distally. Beyond this region, we also observed that H3K9me levels decline sharply but remain detectable, consistent with previous observations^20,21^. To examine euchromatic features, we measured acetylation of histone H3 at lysine 14 (H3K14ac), a mark associated with active transcription^40^. H3K14ac displays an inverse pattern to H3K9me, with higher levels in telomere-distal regions and sporadic enrichment at individual SH genes (Fig. 1d). These chromatin profiles suggest a non-uniform landscape within the SH domain.

We next assessed whether these chromatin features correlate with transcriptional output by performing reverse transcription quantitative PCR (RT-qPCR) assays in *SD4[2R*+*]* and in a derivative lacking Clr4, the sole H3K9 methyltransferase in *S. pombe*. Downstream of *tlh2*, the SH region contains poorly characterized genes that likely arose through gene duplication events and exhibit varying levels of sequence similarity, along with several dubious ORFs (Supplementary Fig. S1a). Most SH genes exhibit low transcript levels in the WT background dependent on Clr4, with telomere-proximal genes such as *tlh2* repressed by up to 1000-fold (Fig. 1e). The variation in gene expression likely reflects differences in chromatin structure, while also being influenced by intrinsic differences in promoter strength. In contrast, genes in the adjacent SU region display 500- to 1000-fold higher expression that is Clr4-independent (Fig. 1e, and Supplementary Fig. S1b). Notably, two SH genes, *SPBCPT2R1.04c* and *SPBB2B2.19c*, located ~28 and ~38 kb from the telomere, also show high expression and only weak dependence on Clr4, indicating a distinct chromatin context.

To assess whether these features are strain-specific, we compared *SD4[2R*+*]* to FY1193, a derivative of the commonly used laboratory strain 972^41^. Although SH sequences vary in DNA sequence and segment copy numbers among different laboratory and wild strains^27^, this variation is primarily confined to the telomere-proximal SH-P region, which is excluded from our analysis. We found that SH gene repression is generally stronger in *SD4[2R*+*]* than in FY1193, but the overall expression pattern is conserved (Supplementary Fig. S1c). These findings indicate that the observed heterogeneity in *SD4[2R*+*]* is not due to strain-specific variation or the loss of SH sequences on the other chromosome arms.

### Position-specific single-cell reporters identify distinct silencing states across the SH domain

**Fig. 2:**
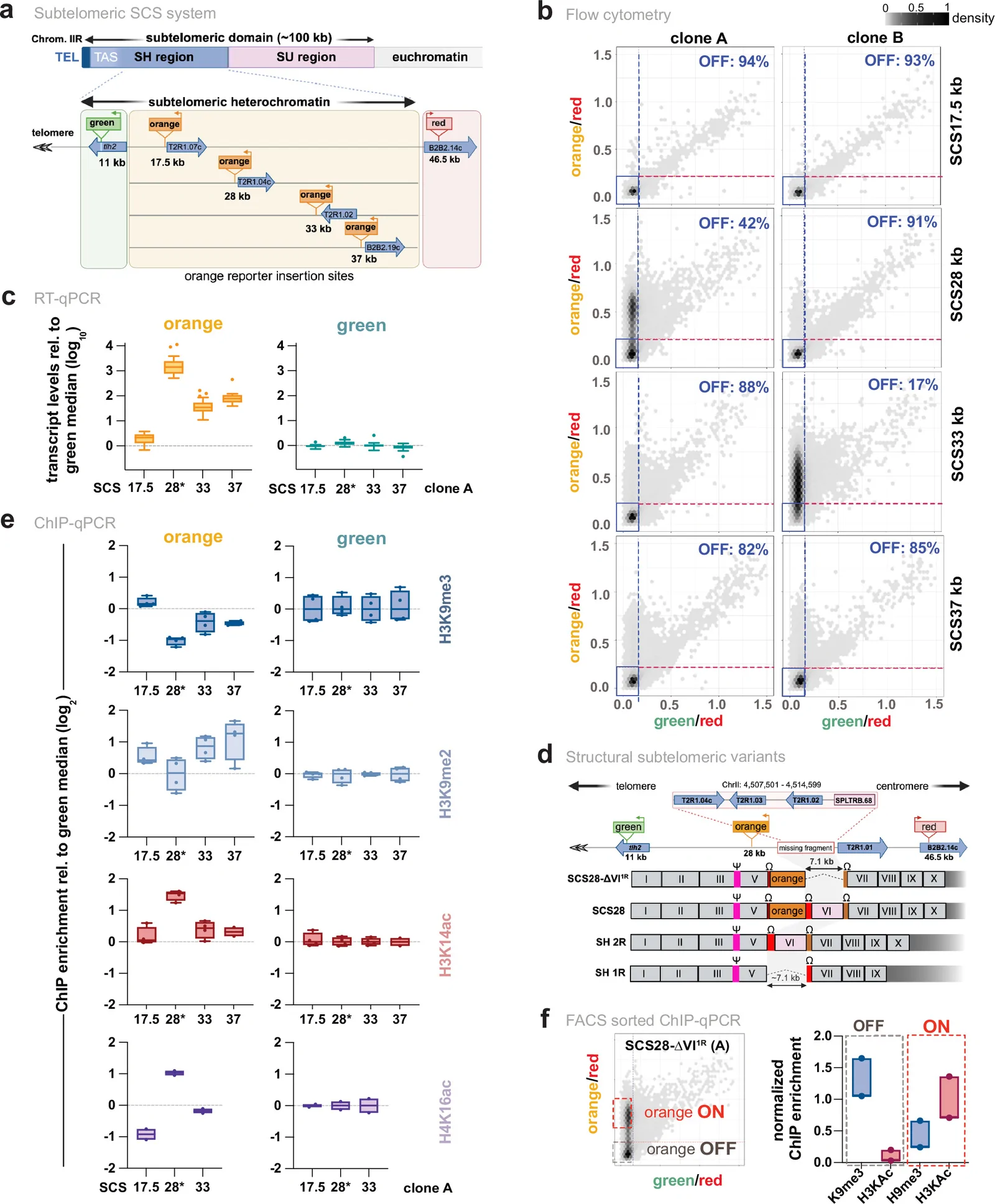
Position-specific single-cell reporters identify distinct silencing states across the SH domain. **a** Schematic of the subtelomeric SCS reporter system integrated into the SH region of chromosome IIR. Each strain contains ‘green’ and ‘red’ reporters at fixed positions within *tlh2* and at 46.5 kb, respectively, and an ‘orange’ reporter at one of four SH positions. Created in BioRender. Braun, S. (2026) https://BioRender.com/5ucojhg. **b** Two-dimensional hexbin density plots showing green (*x*-axis) and orange (*y*-axis) fluorescence normalized to red. Blue and red dashed lines indicate OFF thresholds for ‘green’ and ‘orange’, respectively. Two clones are shown per construct (A, left; B, right). The scale bar indicates relative cell density, with bin cutoffs as fractions of the most populated bin. **c** RT-qPCR analysis of ‘orange’ (left) and ‘green’ (right) reporters (clone A), normalized to *act1* and shown relative to median ‘green’ reporter expression. Tukey box-whisker plots show the median and IQR; whiskers extend to extreme values within 1.5× IQR. Independent biological replicates: *n* = 20 (SCS17.5-A), 16 (28-A*), 29 (33-A), and 18 (37-A). The asterisk in 28-A* denotes a 7.1-kb deletion (see text). **d** Subtelomeric SH regions of Tel1R and Tel2R based on common block sequences (modified from Oizumi et al.^27^). A 7.1-kb fragment corresponding to block VI is absent from both the natural SH1R variant (bottom) and SCS28-A (top, SCS28-ΔVI^1R^). Pink, red, and brown boxes denote Ψ and Ω homologous box sequences; the reporter insertion in Ω is in orange. Created in BioRender. Braun, S. (2026) https://BioRender.com/9jd4gle. **e** ChIP-qPCR analysis of H3K9me3, H3K9me2, H3K14ac and H4K16ac (top to bottom) at ‘orange’ and ‘green’ (clone A). Input-normalized IP signals were pool-normalized to the mean of subtelomeric loci (see “Methods” and Source Data) and are shown relative to median ‘green’ signal (log_2_ scale). Box-and-whisker plots show all data points; whiskers indicate minimum and maximum. Independent biological replicates: *n* = 4, except H3K14ac for 37-A and H4K16ac (all reporters), *n* = 2. **f** ChIP-qPCR on flow cytometry-sorted populations of SCS28-ΔVI^1R^. ‘Green’^OFF^ cells were sorted into ‘orange’^ON^ and ‘orange’^OFF^ populations (see “Methods” and Source Data) and analyzed for H3K9me3 and H3 pan-acetylation, normalized to pericentromeric *cen-dg* repeats and *act1*, respectively. Data points represent the mean of three technical ChIP replicates from each of two independent biological experiments (*n* = 2).

To dissect position-specific heterogeneity in silencing across the SH domain, we developed a single-cell sensor (SCS) reporter system that overcomes the confounding effects of variable promoter strength in endogenous genes and high sequence similarity among SH genes (Fig. 2a). This system builds on our previously established single-cell heterochromatin spreading sensor^12^ with specific adaptations for the SH region. It comprises three transcriptionally encoded fluorescent reporters positioned at defined loci across the SH domain, enabling precise, locus-specific quantification of heterochromatin-mediated silencing in single cells under a uniform promoter context. To monitor telomere-proximal silencing, we integrated a green fluorescent reporter (superfolder GFP under the *ade6* promoter, *ade6p*; “green”) 11 kb from the telomere of *SD4[2R*+*]*, adjacent to the RNAi-dependent *tlh2* nucleation site^20^. Additional reporters (monomeric Kusabira Orange 2 under *ade6p*; “orange”) were inserted at increasing distances from the telomere (17.5 kb, 28 kb, 33 kb, and 37 kb) to survey nucleation-distal regions. A third reporter (E2Crimson under the *act1* promoter; “red”) was integrated at 46.5 kb, marking the SH-SU transition and serving as an internal control to correct for cell-to-cell transcriptional variability caused by intrinsic and extrinsic noise.

We quantified silencing across thousands of cells during logarithmic growth by flow cytometry, analyzing two independent clones for each reporter (clones A and B; see “Methods”). Fluorescence signals from “green” and “orange” reporters were normalized to the “red” control and scaled to the fully derepressed state in *clr4Δ* cells, as described previously^12^. Hexbin plots of normalized fluorescence intensities revealed non-uniform silencing distributed across the SH region, consistent with the transcriptional heterogeneity of endogenous genes (Fig. 1). The “green” and “orange” reporters at 11 kb and 17.5 kb, respectively, exhibited strong silencing in both clones, with over 90% of cells in the OFF states, consistent with their proximity to the *tlh2* nucleation site (Fig. 2b). At 28 kb, silencing became heterogeneous, particularly in clone A, which displayed a bimodal distribution of transcriptionally silenced and active cells. We also observed clone-specific differences at 33 kb, whereas silencing at 37 kb was robust in both clones (82–85%), though weaker than at telomere-proximal sites. To validate the chromatin-dependent behavior of the reporters, we measured transcript levels by RT-qPCR (Fig. 2c). Consistent with flow cytometry data, the “orange” reporter at 17.5 kb shows low expression similar to the “green” reporter at 11 kb (Fig. 2c). The “orange” reporter at 28 kb (clone A) is ~1000-fold more active, and reporters at 33 kb and 37 kb show intermediate expression levels (~50–100-fold above green). Conversely, transcript levels of the “green” reporter are not affected by the varying insertion positions of the “orange” reporter. Similarly, while the “red” reporter is located close to the silent SH region, its expression is robust and largely unaffected by the presence of heterochromatin (Supplementary Fig. S2a). These findings demonstrate that our position-dependent reporters accurately capture the spatial heterogeneity of subtelomeric gene silencing with greater resolution than endogenous gene readouts.

To test whether reporter insertion perturbs local gene expression, we profiled the transcript levels of endogenous genes. Overall expression patterns were preserved across reporter strains in both wild-type and *clr4∆* backgrounds (Supplementary Fig. S2b), with only minor changes for genes near the reporter insertion sites. However, in SCS28 clone A, three genes adjacent to the 28 kb reporter (*SPBCPT2R1.04c*, *SPBCPT2R1.03*, and *SPBCPT2R1.02*) were completely absent. These genes belong to a common sequence block (VI) flanked by two nearly identical Ω homologous box sequences (Fig. 2d). This 7.1-kb sequence is also naturally absent from the Tel1R arm of the reference laboratory strain 972, likely due to a historical recombination event between the Ω elements^27^. While this structural variant may contribute to the altered silencing profile in clone A, additional evidence indicates that epigenetic mechanisms are also involved (see below). To reflect its structural similarity to the truncated Tel1R chromosome arm, we hereafter refer to clone A as SCS28-∆VI^1R^.

We next examined histone modifications at the reporter loci using ChIP-qPCR. All reporters display high H3K9me2 and H3K9me3 levels, comparable to those at pericentromeric *cen-dg* repeats (Supplementary Fig. S2c). However, we observed variation among the “orange” reporters consistent with the expression data from flow cytometry (Fig. 2b) and RT-qPCR (Fig. 2c), although differences in these repressive chromatin marks are considerably less pronounced than the corresponding differences in expression levels. In particular, the “orange” reporter in SCS28-∆VI^1R^, which is expressed more than 100-fold higher than reporters at other sites, exhibits only a twofold reduction in H3K9 methylation. By contrast, H3K14ac levels are markedly increased at the “orange” reporter in SCS28-∆VI^1R^ (Fig. 2c). Consistent with this, deletion of the H3K14 histone acetyltransferase Mst2 reduces “orange” expression in SCS28-∆VI^1R^ and, to a lesser extent, in SCS33 and SCS37 (Supplementary Fig. S3a,b), in agreement with previous reports implicating Mst2 in subtelomere silencing^42^. H4K16ac shows a similar pattern, consistent with its enrichment at several active promoters and its reported role in antagonizing heterochromatin spreading^43^. Because reporter expression is bimodal in SCS28-∆VI^1R^, these elevated histone acetylation marks may also reflect a bimodal chromatin state; however, it remained unclear whether acetylation increased uniformly across all cells or specifically in cells with high (ON) “orange” expression. To address this question, we used flow cytometry to sort “orange” ON and OFF cell populations, followed by ChIP-qPCR to quantify H3K9me3 and H3 tail acetylation. These experiments revealed that H3K9me3 is reduced in ON cells relative to OFF cells, consistent with a weakened heterochromatic state. In contrast, H3 tail acetylation is nearly absent in OFF cells while it increases dramatically in ON cells (Fig. 2f). Together, these results demonstrate that reporter activity is associated with distinct chromatin states, with histone acetylation correlating most closely with the gene expression state.

### Silencing of telomere-distal SH regions is maintained independently of common nucleation pathways

**Fig. 3:**
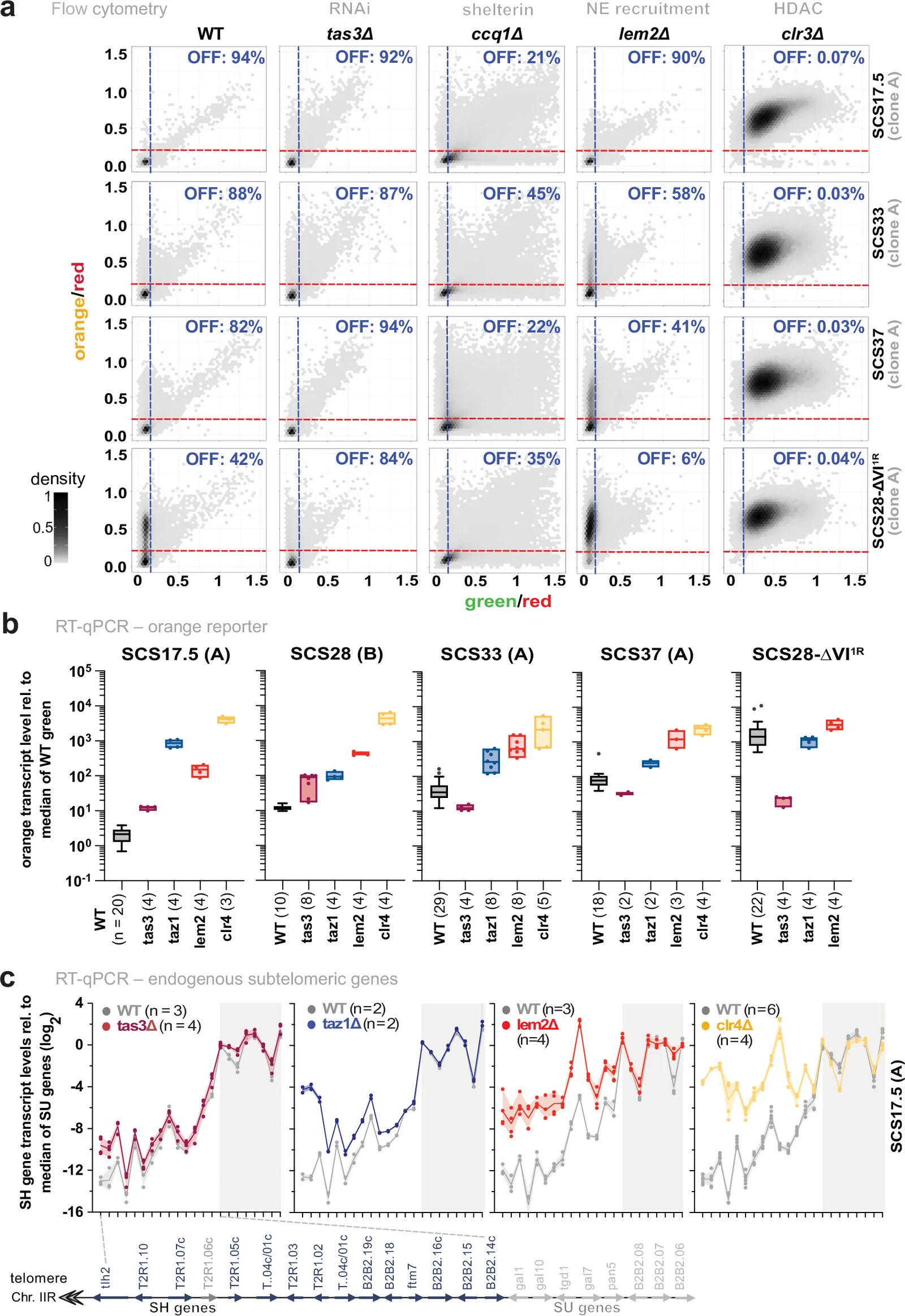
Silencing of telomere-distal SH regions is maintained independently of common nucleation pathways. **a** Two-dimensional hexbin density plots showing red-normalized green (*x*-axis) and orange (*y*-axis) fluorescence for SCS reporters in various genetic backgrounds. Blue and red dashed lines indicate OFF thresholds for green and orange reporters, respectively. (see Fig. 2). Columns represent wild type (WT), *tas3Δ*, *ccq1Δ*, *lem2Δ*, and *clr3Δ*; rows represent SCS constructs with variable ‘orange’ reporter at 17.5, 33, or 37 kb in full-length SH-2R and SCS28-ΔVI^1R^. WT data are identical to Fig. 2b. *tas3∆* reporters were analyzed independently, with corresponding WT controls in Supplementary Fig. S3a. **b** RT-qPCR analysis of ‘orange’ reporter transcripts at 17.5, 28, 33 and 37 kb in SH-2R, and at 28 kb in SCS28-ΔVI^1R^ in WT, *tas3Δ*, *taz1Δ*, *lem2Δ* and *clr4Δ*. Clones (A, B) are indicated in brackets. Transcript levels were normalized to *act1* and are shown relative to the median ‘green’ reporter expression in WT samples. For *n* > 10, Tukey box-and-whisker plots show the median and interquartile range; whiskers extend to the most extreme values within 1.5× the interquartile range. For *n* ≤ 10, floating bars show the median and range (minimum to maximum). Individual data points are shown; *n* denotes independent biological replicates and is indicated below each sample. **c** RT-qPCR analysis of endogenous SH and SU genes across genetic backgrounds in SCS17.5 (clone A). Profiles are shown for *tas3Δ*, *taz1Δ*, *lem2Δ*, and *clr4Δ* with WT values (grey) for comparison. Transcript levels were normalized to *act1* and are shown relative to median SU-region expression in WT (log₂ scale). Individual biological replicates are shown with median and range; *n* is indicated in each panel. The schematic shows SH (blue) and SU (grey) gene organization; grey-marked SH genes were excluded from analysis.

Next, we examined whether previously defined SH heterochromatin nucleation and silencing pathways impact our SCS reporters. We deleted components of the RNAi- and shelterin-dependent pathways, as well as the inner nuclear membrane protein Lem2 (Supplementary Fig. S4a). Loss of the RITS scaffold protein Tas3, the shelterin component Ccq1, or the nuclear membrane protein Lem2 results in modest derepression of the “green” and most “orange” reporters, consistent with their overlapping roles at subtelomeres (Fig. 3a). Despite these similarities, each mutant displays a distinct phenotype. Consistent with the localized and redundant roles of RNAi and shelterin in heterochromatin nucleation, *tas3∆* exhibits a subtle expression increase of the telomere-proximal 17.5 reporter, whereas *ccq1*∆ displays stronger and broader defects due to additional shelterin binding sites within the SH region^20,36^. In contrast, *lem2*∆ primarily affects telomere-distal silencing, consistent with the broader role of Lem2 in subtelomeric heterochromatin maintenance. By comparison, deletion of the HDAC Clr3, a component of SHREC, which is redundantly recruited by Ccq1 and Lem2^30,31,36^, leads to full derepression at every locus (Fig. 3a, and Supplementary Fig. S4b). Intriguingly, whereas the “orange” reporter in SCS28-∆VI^1R^ displays a bimodal repression pattern in WT cells, this bimodality is suppressed upon deletion of *tas3* or *ccq1*, resulting in reduced “orange” expression. This suggests that loss of RNAi and shelterin redirects silencing factors from regions dependent on these pathways, such as telomere-proximal and pericentromeric loci, to the “orange” reporter in SCS28-∆VI^1R^.

We confirmed these position-specific requirements by RT-qPCR (Fig. 3b). When examining the different reporters, some common and unique behaviors emerged. Deletion of *clr4* abolishes silencing at every reporter, confirming that the observed expression patterns in wild-type cells reflect distinct heterochromatin states rather than sequence-dependent effects. Conversely, deletion of *tas3* predominantly affects telomere-proximal reporter sites. At 17.5 kb, transcript levels increase modestly (~10-fold), consistent with our flow cytometry data and previous findings^20,29^. However, reporters at 33 and 37 kb remain unchanged or show slightly enhanced repression. We observed a similar pattern following deletion of *ago1*, which encodes the Argonaute subunit of RITS (Supplementary Fig. S4c). Notably, the high expression levels observed in WT SCS28-∆VI^1R^ are reduced by ~100-fold in *tas3∆* or *ago1∆*, reaching values comparable to those in the non-truncated SCS28 strain (Fig. 3b, and Supplementary Fig. S4c). Loss of *taz1* also leads to strong derepression of the 17.5 kb “orange” reporter and a moderate increase at telomere-distal reporters. Remarkably, although RNAi and Taz1 have been reported to act redundantly at subtelomeric loci^20,36^, their combined loss does not increase “orange” expression in SCS28-∆VI^1R^ but instead further reduces it compared with the *taz1*∆ single mutant (Supplementary Fig. S3d). Deletion of *lem2* elevates orange expression across reporters, whereas single-cell data obtained by flow cytometry indicate a stronger effect at distal reporters (Fig. 3b). This difference may indicate that a subset of cells contributes disproportionately to the RT-qPCR signal. The position-specific functions of Tas3, Taz1, and Lem2 also apply to endogenous subtelomeric SH genes, both in the presence and absence of reporter integration (Fig. 3c, top panels; Supplementary Fig. S4e). Together, these findings indicate that the contributions of RNAi and shelterin to subtelomeric silencing are largely restricted to telomere-proximal regions.

### Common block sequences locally control gene silencing at the subtelomere

**Fig. 4:**
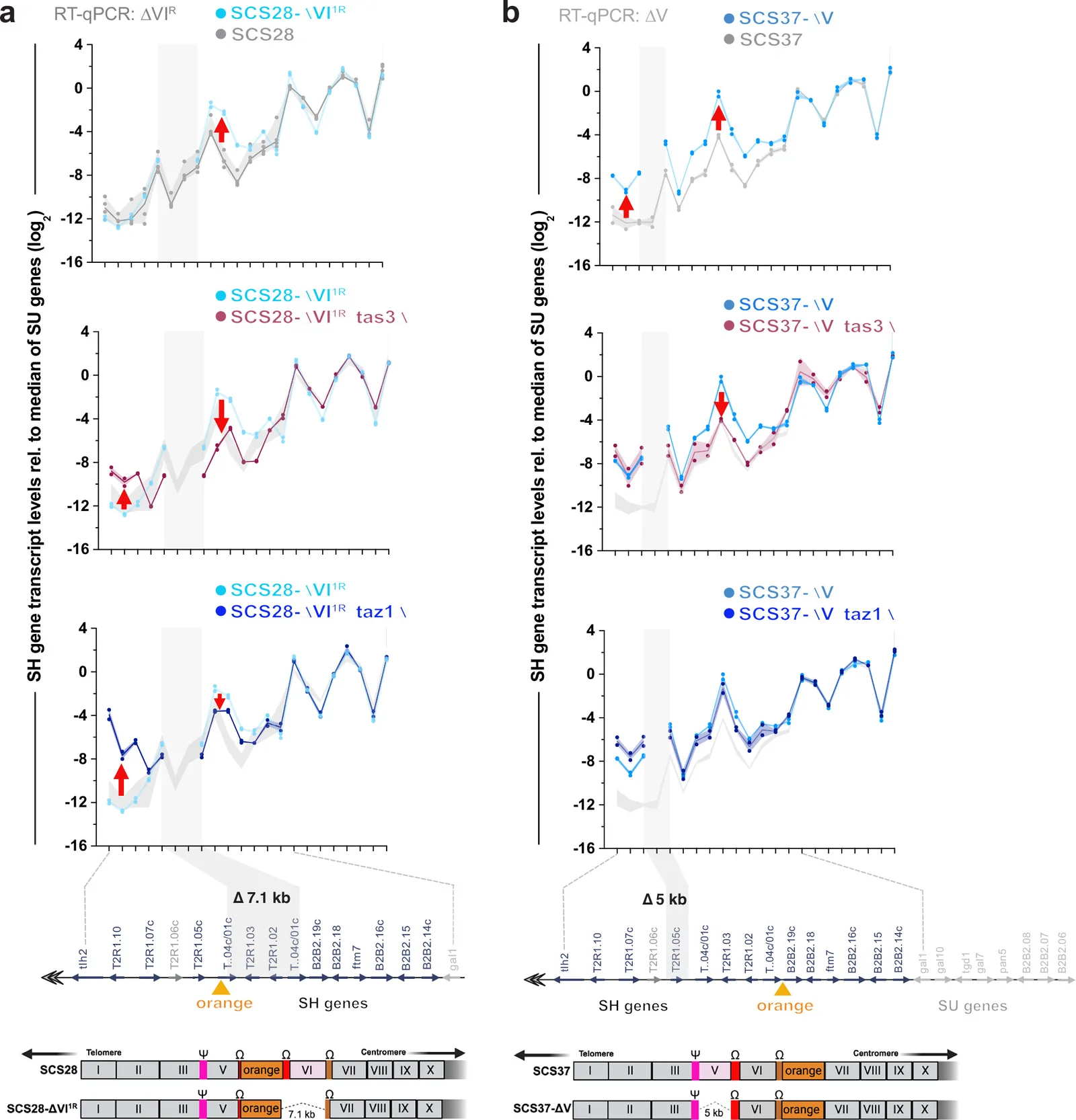
Common block sequences locally control gene silencing at the subtelomere. **a** RT-qPCR analysis of the effect of the 7.1-kb block VI deletion on endogenous subtelomeric gene expression. Top: SCS28 SH-2R (SCS28; grey) versus the SH-1R-like block VI deletion strain (SCS28-ΔVI^1R;^ light blue). Middle: SCS28-ΔVI^1R^ (light blue) versus SCS28-ΔVI^1R^ *tas3*Δ (dark red). Bottom: SCS28-ΔVI^1R^ (light blue) versus SCS28-ΔVI^1R^ *taz1*Δ (dark blue). Two biological replicates (*n* = 2) from a representative experiment are shown as individual data points with mean and range, except for SCS28-ΔVI^1R^, WT: *n* = 4. **b** RT-qPCR analysis of the effect of the 5-kb block V deletion on endogenous subtelomeric gene expression. Top: SCS37 SH-2R (SCS37; grey) versus the block V deletion strain (SCS37-∆V; medium blue). Middle: SCS37-∆V (medium blue) versus SCS37-∆V *tas3*Δ (dark red). Bottom: SCS37-∆V (medium blue) versus SCS37-∆V *taz1*Δ (dark blue). Two biological replicates are shown as individual data points with mean and range. For (**a**) and (**b**), transcript levels were normalized to *act1* and are shown relative to the median expression of SU-region genes (log₂ scale). The shaded area in the middle and bottom panels indicates the data range of the corresponding non-deletion strain. Red arrows indicate loci with substantial expression differences between the corresponding strains. Schematics below the graphs indicate the positions of the reporter insertions and the corresponding block VI or V deletions. Created in BioRender. Braun, S. (2026) https://BioRender.com/acno2l3.

The differential effects of heterochromatin factors across the subtelomere, with nucleation factors exerting stronger effects toward the telomere end, raise the question of how local silencing is controlled. Two general models could account for this: (1) silencing is determined primarily by distance from the nucleation site; or (2) silencing is controlled by local cis-acting elements. To distinguish between these models, we assessed the impact of deleting two common block sequences, VI and V (Fig. 4a, b).

We first focused on the structural variant SCS28-∆VI^1R^, which lacks the 7.1-kb block VI and shows weaker silencing of the “orange” reporter than its non-truncated sister clone (Fig. 2). We examined endogenous gene expression in both reporter strains by RT-qPCR. Consistent with the position-specific effects of RNAi and shelterin mutants (Fig. 3b, c), gene expression near *tlh2* is unaffected by deleting block VI, which lies approximately 15 kb downstream of the RNAi-dependent nucleation site (Fig. 4a, top panel). However, several genes downstream of ∆VI (*SPBPB2B2.19c, SPBPB2B2.18*, and *ftm7*) are moderately derepressed (~10-fold), despite their increased proximity to the nucleation site, indicating that reducing this distance does not enhance their silencing. Conversely, additional deletion of *tas3* partially restores silencing of these genes in SCS28-∆VI^1R^ (Fig. 4a, middle panel), mirroring the behavior of the “orange” reporter (SCS28-∆VI^1R^ *tas3*∆, Fig. 3b). As expected, *tlh2*-proximal genes are upregulated in SCS28-∆VI^1R^ *tas3*∆, consistent with Tas3’s role in RNAi-dependent nucleation. We observed a similar pattern upon deletion of *taz1*, although the rescue of silencing downstream of ∆VI appears weaker (Fig. 4a, bottom panel).

Next, we deleted block V, which lies upstream of block VI and spans 5 kb. Since block V overlaps the 28-kb reporter insertion site, we performed the deletion in the SCS37 reporter background (SCS37-∆V). While we observed derepression of genes both upstream and downstream of ∆V, this effect was more pronounced for *tlh2* and adjacent genes (Fig. 4b, top panel). Additional deletion of *tas3* partially restores silencing of genes downstream of ∆V, as observed for ∆VI. In contrast, *tlh2*-proximal genes display similarly elevated expression in SCS37-∆V and SCS37-∆V *tas3*∆, indicating overlapping contributions of block V and RNAi to silencing of genes upstream of the deletion (Fig. 4b, middle panel). By comparison, while additional deletion of *taz1* does not restore silencing in SCS37-∆V, deletion of block V partially alleviates the silencing defects of *taz1*∆ at *tlh2*-proximal genes (Fig. 4b, bottom panel). We observed similar effects upon deletion of block V in the parental strain lacking reporter insertions, although changes in gene expression were weaker and largely restricted to genes upstream of the block V deletion. Nevertheless, combining the block V deletion with either *tas3* or *taz1* partially alleviates the silencing defects, as observed in the reporter strains (Supplementary Fig. S5). Overall, these findings argue against a simple distance-dependent model, in which repeated blocks act primarily as passive spacers for heterochromatin spreading. Instead, they indicate that the common blocks function as distinct elements that locally shape subtelomeric silencing, as evidenced by the distinct consequences of deleting either block VI or block V, either alone or in combination with *tas3*∆ or *taz1*∆.

We next investigated whether specific cis-regulatory elements within the common blocks directly regulate local silencing. Adjacent to Ω homologous box sequences, block VI is also flanked by two LTRs, *SPLTRB.69* and *SPLTRB.68* (Supplementary Fig. S6a); the latter is absent in SCS28-∆VI^1R^. Because LTRs have been proposed to act as boundary elements separating euchromatin from heterochromatin domains^19,44^, we hypothesized that the loss of *SPLTRB.68* might underlie the altered silencing phenotype in SCS28-∆VI^1R^. To test this, we deleted *SPLTRB.68* using CRISPR in the non-truncated sister clone SCS28 and another reporter strain, SCS33. Neither deletion substantially affects “orange” expression (Supplementary Fig. S6b), indicating that loss of *SPLTRB.68* alone is insufficient to explain the phenotype. However, removing a different LTR (*SPLTRB.67*) located further downstream caused a >10-fold increase in “orange” expression relative to the corresponding WT strain (Supplementary Fig. S6b). These findings suggest that while *SPLTRB.68* is largely dispensable for repression; specific LTR elements, such as *SPLTRB.67*, can modulate local silencing and contribute to the organization of SH heterochromatin.

Having found only limited support for a role of LTRs in this phenotype, we next asked whether common block sequences also harbor heterochromatin nucleation sites. SH genes adjacent to the 7.1-kb fragment in SCS28-∆VI^1R^ maintain silencing independently of RNAi and the shelterin complex (Fig. 4a), suggesting that this segment or nearby sequences may function as a cryptic heterochromatin nucleation site capable of recruiting Clr4, independently of these nucleation pathways and pre-existing H3K9 methylation. To identify such sites, we examined Clr4 enrichment in an *ago1*∆ *H3K9R* strain at subtelomeric regions and detected a small but distinct Clr4 peak near the 7.1-kb segment within block V (Supplementary Fig. S6c, d). Comparable peaks are present on both Tel1L and Tel2R at similar, though not identical, positions. Intriguingly, the Clr4 binding site on Tel2R is adjacent to H3K9me3 peaks that persist in Swi6^*HP1*^ mutants deficient in heterochromatin spreading (Supplementary Fig. S6e)^45^. These Clr4 and H3K9me3 peaks overlap with or are close to the ncRNAs *SPNCRNA.2012*, *SPNCRNA.6640*, and *SPNCRNA.6641*. Similar ncRNAs have been linked to RNAi-independent Clr4 recruitment at the pericentromere and heterochromatin islands^15,18,46–48^. These findings suggest that this telomere-distal region may harbor cryptic, ncRNA-dependent nucleation sites.

Together, these data indicate that common block sequences define autonomous subtelomeric silencing domains rather than acting as passive spacers for heterochromatin spreading. Telomere-proximal regions remain dependent on RNAi- and shelterin-mediated nucleation, whereas more distal regions may harbor cryptic, potentially ncRNA-dependent nucleation sites and are further modulated by LTR elements.

### Silencing at distal subtelomeric subdomains has unique genetic requirements

**Fig. 5:**
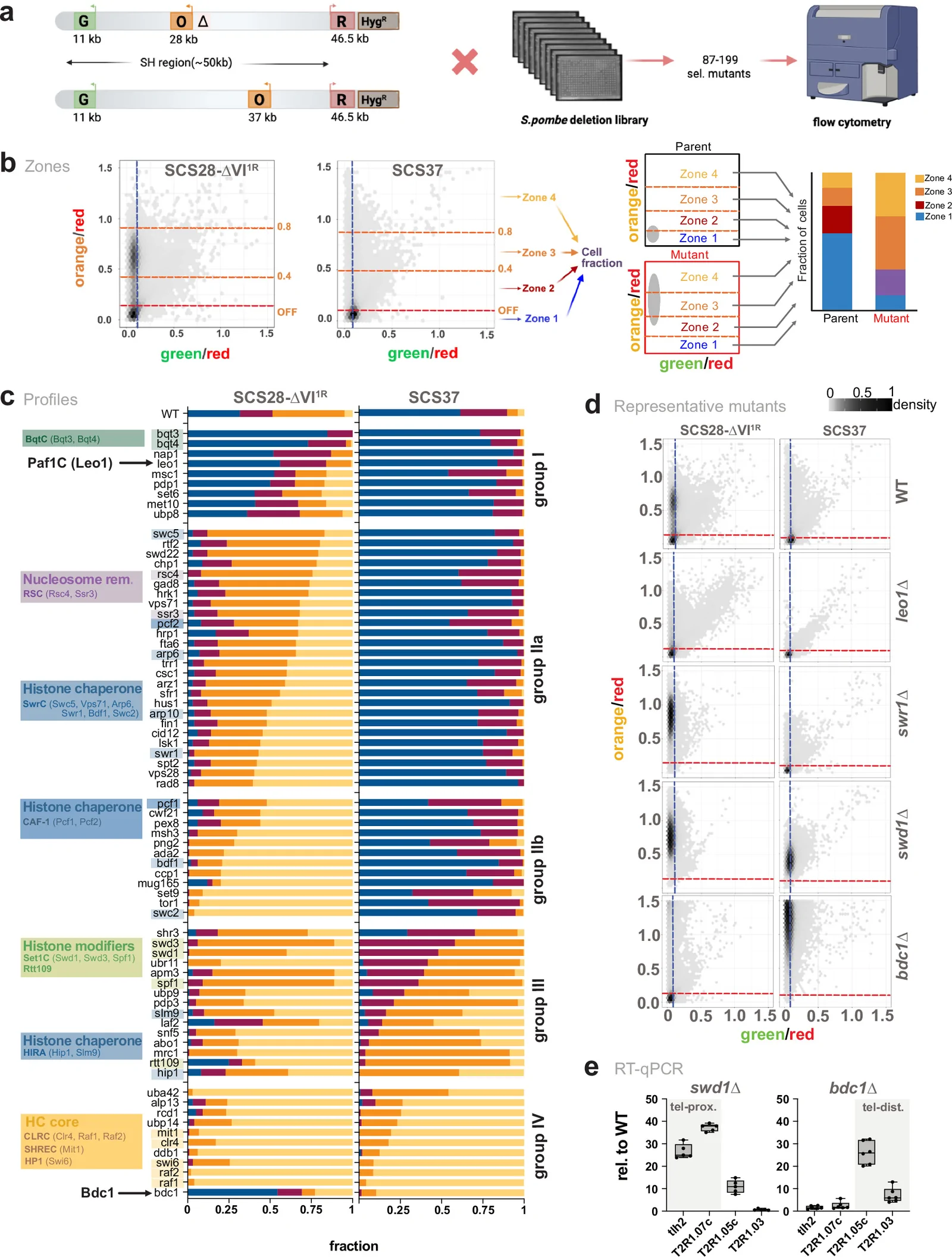
Silencing at distal subtelomeric subdomains has unique genetic requirements. **a** Schematic of the fluorescence-based targeted screen. SCS28-ΔVI^1R^ and SCS37 contain orange reporters at 28 kb or 37 kb, respectively, with ‘green’ and ‘red’ reporters fixed at 11 kb and 46.5 kb. Both strains were crossed with a deletion library of ~3000 non-essential genes. A subset of 199 for SCS28-ΔVI^1R^ and 87 SCS37 mutants were selected for flow cytometry analysis. Created in BioRender. Braun, S. (2026) https://BioRender.com/831pt7a. **b** Two-dimensional hexbin density plots showing green (*x*-axis) and orange (*y*-axis) fluorescence normalized to red. OFF thresholds are as in Fig. 2b. Orange fluorescence was stratified into four zones by increasing orange/red ratio, with cutoffs at the OFF threshold, 0.4, and 0.8. The fractions in each zone were quantified, with increased fractions in zone 3 and 4 indicating orange reporter activation. **c** Stacked bar plots showing cell fractions across zones 1-4 in wild-type and mutants in SCS28-ΔVI^1R^ (left) or SCS37 (right). Mutants were clustered by zone distribution using optimal k-means clustering (*k* = 5; see Supplementary Fig. S7d); groups IIa and b were combined for subsequent analysis. Within groups, mutants are ordered by increasing fractions in zones 3 and 4 in SCS28-ΔVI^1R^ (left), with corresponding zone SCS37 distributions shown on the right. Genes are grouped by functional category, including transcriptional regulators, chromatin remodelers, histone chaperones, histone modifiers, and heterochromatin core components. **d** Representative two-dimensional hexbin density plots showing green (*x*-axis) and orange (*y*-axis) fluorescence normalized to ‘red’ for SCS28-ΔVI^1R^ and SCS37 reporter constructs. **e** RT-qPCR analysis of endogenous gene expression in independently generated *swd1* and *bdc1* knockout strains in the parental *SD4[2R*+*]* background lacking the reporters. Transcript levels were normalized to *act1* and are shown relative to the median WT value. Independent biological replicates (*swd1∆*, *n* = 5; *bdc1∆*, *n* = 6) from three independent experiments are shown as individual data points. Tukey box-and-whisker plots show the median and interquartile range; whiskers extend to the most extreme values within 1.5× the interquartile range. Shaded areas highlight telomere-proximal (*tlh2*, *SPBCPT2R1.07c*; left) and telomere-distal (*SPBCPT2R1.05c*, *SPBCPT2R1.03*; right) genes.

The phenotypic differences among core pathway mutants support the idea that silencing at each locus relies on distinct genetic requirements, potentially involving distinct sets of trans-acting factors operating across the subtelomeric region. To explore this further, we performed a targeted screen using the SCS28-∆VI^1R^ and SCS37 reporters, which represent different structural variants and display distinct epigenetic behaviors. SCS28-∆VI^1R^ additionally has the advantage that it allows the detection of silencing antagonists. We crossed these reporters to a curated set of chromatin factor mutants (partial list from Greenstein et al.^49^; see Supplementary Data S4–S6) and examined the resulting strains by flow cytometry (Fig. 5a). We prioritized genes that affect the silencing at “orange”, as factors leading to de-repression of “green” overlapped with known, general regulators of heterochromatin (Supplementary Fig. S7a, b). Because strains in the mutant library carry intact SH regions on all subtelomeric arms, the genetic crosses generated F1 progeny with mixed subtelomeric configurations. Nonetheless, flow cytometry analysis revealed no detectable differences between these progeny and the parental reporter strains (Supplementary Fig. S7c). To quantify silencing phenotypes of the SCS28-∆VI^1R^ and SCS37 reporters, we analyzed the distribution of individual cells by dividing the population into four zones based on the level of “orange” repression and quantified the fraction of cells in each zone in WT and mutant strains (Fig. 5b). For SCS37, in which the “orange” reporter is strongly repressed in WT cells, most mutants exhibit loss of silencing, whereas mutants in SCS28-∆VI^1R^ display both loss and gain of silencing (Supplementary Fig. S7a, b). Using the combined results from both reporter screens, we performed k-means clustering, which identified five major clusters (Supplementary Fig. S7d). Because two clusters displayed highly similar phenotypes and were enriched for members of the same protein complexes, we classified them together as group II, comprising subgroups IIa and IIb. Group I contains mutants with enhanced silencing primarily in SCS28-∆VI^1R^, whereas subgroups IIa and IIb comprise mutants with silencing defects exclusively in SCS28-∆VI^1R^. Group III contains mutants with strong silencing loss in SCS28-∆VI^1R^ and partial silencing defects in SCS37, whereas group IV comprises mutants with pronounced silencing defects of both reporters.

A noteworthy example from group I (mutants that enhance silencing of SCS28-∆VI^1R^) is the Paf1C subunit Leo1, which also partially enhances silencing in SCS37 (Fig. 5c). This finding is consistent with previous reports showing that Paf1C promotes nucleosome turnover while antagonizing heterochromatin assembly and spreading^50–52^. Additional factors include the nuclear envelope-bound proteins Bqt3 and Bqt4, which are implicated in telomere tethering (Fig. 5c). We also identified members of Ino80C (Ies2, Ies4, Iec1 Iec3) and two Jumonji proteins, Jmj2 and Jmj4, which are potentially involved in H3K4 trimethylation^53^ (Supplementary Fig. S7a). This link to H3K4 methylation may relate to the formation of H3K4me-dependent boundaries in open chromatin (see discussion).

Group II (subgroups IIa and IIb) contains mutants with silencing defects in SCS28-∆VI^1R^. Among those mutants, chromatin remodeling factors display unusual behaviors. Previously, mutations in these genes have been implicated in restricting heterochromatin spreading^49^ and assembly^54^. Surprisingly, we found that several remodeling complex members are required for “orange” silencing in SCS28-∆VI^1R^ but not SCS37, including Swr1C, which is involved in H2A.Z deposition (Swr1, Swc2, Swc5, Arp6, Vps71, Bdf1), and RSC (Rsc4 and Ssr3). We also found that members of the histone chaperones CAF1 (Pcf1, Pcf2) specifically affect SCS28-∆VI^1R^. This behavior contrasts with that at the MAT locus, likely reflecting the role of SH as a buffering sink for free heterochromatin factors^21,55,56^. In this scenario, the gain of silencing observed in remodeler mutants at pericentromeres and the MAT locus comes at the expense of factors redistributed from the SH region, consistent with the much broader sensitivity of the SCS28-∆VI^1R^ reporter to the loss of these remodeling complex members.

Group III mutants exhibit loss of “orange” silencing in both reporters, with a stronger effect in SCS28-∆VI^1R^. In addition to the replication fork-associated protein Mrc1, previously shown to promote heterochromatin inheritance^57–60^, this group is particularly enriched for members of the Set1C complex (Fig. 5c, d) and subunits of the HIRA histone chaperone (Hip1, Slm9; Fig. 5c, and Supplementary Fig. S7e). We validated this telomere-proximal biased silencing effect for the Set1C member Swd1 and the HIRA subunit Hip1 by RT-qPCR (Fig. 5e, and Supplementary Fig. S7f). Although several of these group III genes have been reported to affect heterochromatin silencing^61–63^, mutants in these particular complexes overlap with those we previously showed to specifically disrupt ectopic heterochromatin at the euchromatic *ura4* locus, while having a weaker impact on constitutive sites^49^.

Group IV mutants that disrupted “orange” silencing in both reporters include several previously characterized heterochromatin factors, including the Clr6 deacetylase complex^49^, the E3 ubiquitin ligase adapter protein Ddb1^64^, and members of the H3K9 methyltransferase Clr4 complex and the HP1 homolog Swi6.

Beyond the factors conforming to groups I-IV, we identified several mutants with distinct phenotypes. The most striking example is *bdc1*, which encodes a bromodomain protein of the NuA4 HAT complex. Bdc1 antagonizes silencing at 28 kb but is essential for silencing at 37 kb. We also confirmed its requirement for repression of telomere-distal genes in the parental *SD4[2R*+*]* strain lacking the reporters (Fig. 5e). Bdc1 has not previously been implicated in heterochromatin regulation and may have a unique, context-dependent role at SH domains. Although too few mutants were specific to SCS37 to define a separate group, one notable example is Rtt109, the H3K56 acetyltransferase in *S. pombe*^65^. ﻿While these factors do not belong to a single molecular pathway, their distinct phenotypes underscore the modular nature of SH heterochromatin and the diverse regulatory mechanisms acting on individual subdomains.

### SH chromatin undergoes unique dynamic changes not seen at other constitutive heterochromatin loci

**Fig. 6:**
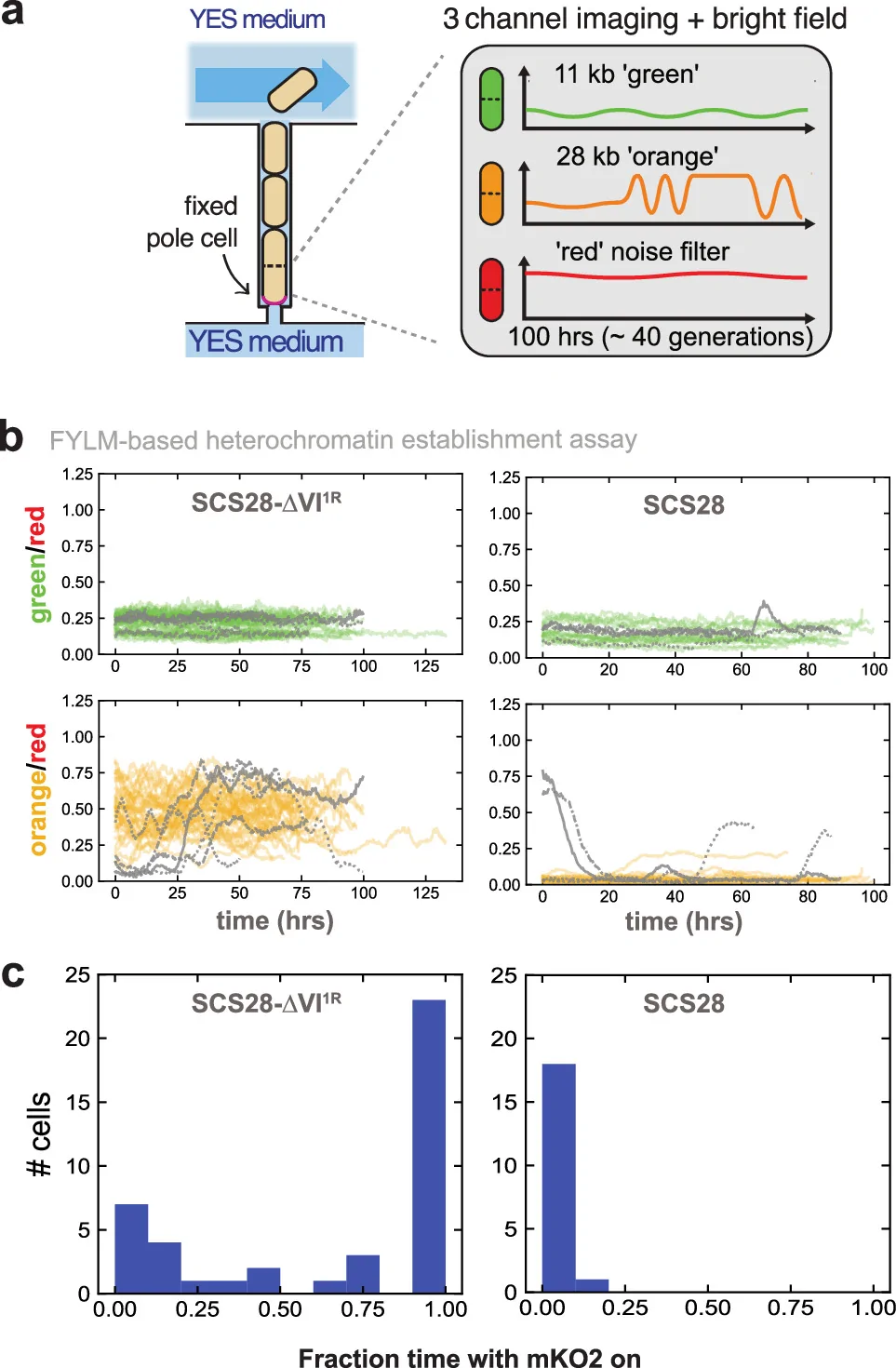
Intergenerational tracking reveals unique dynamic changes in SH chromatin. **a** Schematic of FYLM experiment. **b** Normalized fluorescence traces for cells that maintained the nucleation sensor (‘green’) in the OFF state throughout an experiment. Thresholds for ON and OFF states were determined within each experiment using signals from *clr4Δ* cells (see Methods). Cells were pooled from three independent experiments (total number: SCS28-ΔVI^1R^, *n* = 42 cells; SCS28, *n* = 19 cells). Grey stylized lines indicate cells whose orange-to-red ratio crossed between ON and OFF states during the experiment. **c** Histograms of time spent in ‘orange’ ON and OFF states for each cell. For each cell, the number of hours where the cell’s orange-to-red ratio was above the ON threshold was divided by the total number of hours collected for that cell.

Previously, we documented widespread fluctuation in silencing over time at the MAT locus using a sensor positioned 4 kb distal to the constitutive *cenH* nucleation site, while a sensor at the nucleation site remained silent^12^. To determine whether SH heterochromatin exhibits similar temporal dynamics, we monitored the 28 kb “orange” reporter using the Fission Yeast Lifespan Microdissector (FYLM)^66^, which enables single-cell tracking of reporter expression over more than 40 generations (Fig. 6a). We selected the SCS28-∆VI^1R^ reporter and its non-truncated SCS28 sister strain due to their distinct silencing ground states. A *clr4∆* strain served as a control to scale “green” and “orange” fluorescence in the absence of heterochromatin. Live-cell imaging showed that the “green” reporter, located near the *tlh2* nucleation site, remains fully repressed throughout the experiment in both clones, as expected (Fig. 6b, top traces). In contrast, as predicted from flow cytometry, the “orange” reporter displays clone-specific behavior: the majority of cells in SCS28-∆VI^1R^ occupy fully ON or intermediate states, whereas cells in SCS28 are predominantly OFF. Nonetheless, both clones show moderate switching activity, with infrequent transitions between the ON and OFF states observed in a small number of cells (see example traces in Fig. 6b, depicted in grey). Quantification confirmed that the majority of SCS28-∆VI^1R^ cells remain ON and most SCS28 cells remain OFF for the duration of the experiment, consistent with low-frequency transitions (Fig. 6c).

The temporal behavior of both 28 kb reporters differs markedly from that observed at the MAT locus^12^. There, we observe either extreme fluctuations at “orange” in all cells or no fluctuations at all and across the entire time course, depending on the heterochromatin nucleator. In contrast, cells carrying the SCS28 reporters remain stably ON or OFF throughout the experiment, with only infrequent fluctuations. These findings demonstrate that SH heterochromatin at the ~28-kb region, independent of its precise genetic context, adopts a distinct, metastable epigenetic state.

### The SH-domain contains subdomains with unique epigenetic behaviors

**Fig. 7:**
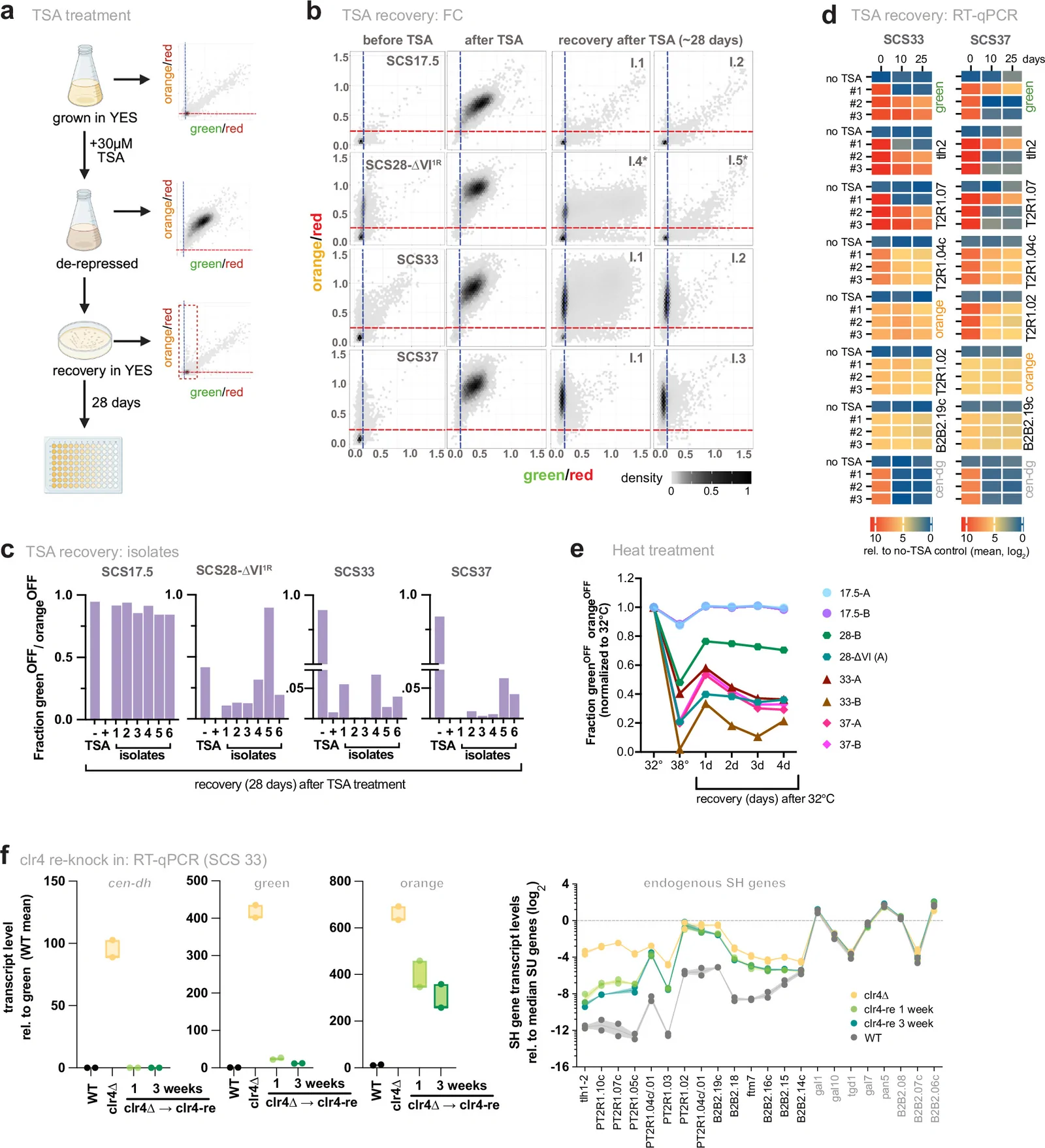
The SH-domain contains subdomains with unique epigenetic behaviors. **a** Schematic of trichostatin A (TSA) treatment and recovery. Created in BioRender. Braun, S. (2026) https://BioRender.com/1d68tam. **b** Two-dimensional hexbin density plots showing green (*x*-axis) and orange (*y*-axis) fluorescence normalized to ‘red’; OFF thresholds as in Fig. 2b. Columns show untreated wild-type (WT), 30 μM TSA treatment, and two independent isolates after 28 days of recovery in nutrient-rich media. Rows represent SCS strains with ‘orange’ reporters at 17.5, 28, 33, or 37 kb; ‘green’ and ‘red’ reporters are as in Fig. 2a. **c** Bar plots showing recovery dynamics after TSA treatment. *y*-axis as the fractions of cells in the ‘orange’ OFF/‘green’ OFF state. Conditions are untreated, 30 µM TSA-treated, and 28-day recovery. Six independent recovery isolates per SCS construct are shown. **d** RT-qPCR analysis of the recovery dynamics of reporter and endogenous subtelomeric transcripts in SCS33 and SCS37 after TSA treatment and recovery after 0, 10, or 25 days. Three independent recovery isolates (#1-3) and an untreated control were analyzed per strain; the day 0 sample is shared among isolates. Transcript levels were normalized to *act1* and are shown relative to mean expression of the corresponding untreated control across days 0, 10, and 25 (log_2_ scale). **e** Recovery of reporter silencing after 38 °C heat treatment and return to 32 °C. The fraction of cells in the ‘orange’OFF/‘green’OFF state was measured before and immediately after treatment and during four days of recovery. Each line represents an SCS reporter clone (A, B) with ‘orange’ at 17.5, 28, 33 or 37 kb. **f** Transcript recovery following *clr4* reintroduction in SCS33-A. Left: RT-qPCR of *cen*-*dg*, ‘green’ (11 kb), and ‘orange’ (33 kb) after *clr4* deletion and reintroduction, with 1 or 3 weeks of recovery. Transcript levels were normalized to *act1* and are shown relative to the mean ‘green’ expression in WT. Right: RT-qPCR of endogenous SH and SU genes in WT, *clr4Δ*, and after 1 or 3 weeks of *clr4* reintroduction, normalized to *act1* and shown relative to median SU expression in WT (log₂ scale). Independent biological replicates (*n* = 2) are shown as mean and range.

Having established metastability at SCS28, we next asked whether the differences in the steady-state distributions among the “orange” reporters (Fig. 2) reflect distinct epigenetic states along the subtelomeres. To test this, we used a classic recovery approach, which monitors heterochromatin re-establishment following transient perturbation with the HDAC inhibitor trichostatin A (TSA)^67^. To globally reset heterochromatin states, we cultured our reporter strains (SCS17.5, 28-∆VI^1R^, 33, and 37) overnight in TSA^12,68^, then removed the inhibitor and propagated cells continuously for up to 28 days (Fig. 7a). For the 28 kb and 33 kb sensors, we examined both clones (A and B). After TSA withdrawal, we propagated multiple isolates of each sensor strain and monitored silencing dynamics over time. All sensors exhibited complete derepression of the “orange” reporter, while the “green” reporter at 11 kb showed partial derepression (Fig. 7b, and Supplementary Fig. S8a). For examining heterochromatin re-establishment, we selected days 12 and 28, by which time silencing profiles had stabilized (Fig. 7b, and Supplementary Fig. S8a–c).

In all strains, we observed rapid re-establishment of silencing of the “green” reporter, indicating that *tlh2*-proximal heterochromatin is quickly restored (Fig. 7b and quantified in Fig. 7c). In contrast, the silencing dynamics of the “orange” reporter vary depending on its genomic position. At 17.5 kb, silencing is uniformly restored, indicating that rapid restoration of *tlh2*-proximal heterochromatin extends to this site. However, at 28 kb, recovery is heterogeneous. Isolates derived from the bimodal parental clone SCS28-∆VI^1R^ adopted a range of states following recovery, from derepressed to fully silenced states. Similarly, TSA-recovered isolates from the non-truncated SCS28 sister clone exhibit both repressed and bimodal silencing profiles, the latter being reminiscent of SCS28-∆VI^1R^ (Supplementary Fig. S8a and quantified in Fig. 7c and Supplementary Fig. S8b). These results suggest that the region around 28 kb can adopt at least two distinct epigenetic states—fully repressed or bimodal—that can emerge spontaneously and persist over time. This further implies that the variability in SCS28-∆VI^1R^ is not solely attributable to genetic difference but is instead also influenced by the specific chromatin context at this locus.

The “orange” reporters at 33 and 37 kb behaved distinctly from both 17.5 and 28 kb. In the unperturbed state, both reporters are stably repressed (Fig. 2b) except for SCS33-B. However, neither locus fully regained silencing following TSA treatment (Fig. 7b, quantified in Fig. 7c, Supplementary Fig. S8a–c), indicating that regions distal to 28 kb harbor a more fragile epigenetic state. This is also evident at the level of the neighboring endogenous genes, which we tracked by RT-qPCR in SCS33 and SCS37 (Fig. 7d). Here, we observed rapid recovery of *cen-dg* in all isolates, whereas “green”, *tlh2*, and *SPBCPT2R1.07c*, which lies downstream of “orange” in SCS17.5, recovered quickly in half the isolates, consistent with flow cytometry (Fig. 7b). In contrast, the telomere distal genes *SPBCPT2R1.04c*, *SPBCPT2R1.02*, and *SPBPB2B2.19c* genes as well as “orange” at either 33 kb or 37 kb experienced only weak recovery.

One possible explanation for the lack of recovery at telomere distal sites is that chromatin in this region is more susceptible to the loss of H3K9-methylated nucleosomes. If so, re-establishing its repressed state may require genetic conditions that stabilize H3K9 methylation. Epe1, a known antagonist of heterochromatin, promotes nucleosome turnover at H3K9-methylated regions^69^. To test this hypothesis, we repeated the TSA recovery experiment in an *epe1Δ* background. Indeed, silencing of the “orange” reporters at 33 kb and 37 kb was partially restored under this condition (Supplementary Fig. 8d), indicating that chromatin at these positions is particularly sensitive to disruption of H3K9 methylation and benefits from protection against Epe1-mediated turnover.

To determine whether this heightened sensitivity extends beyond HDAC inhibition, we next disrupted gene silencing by transient heat stress, an orthogonal perturbation of heterochromatin. We previously found that transient heat exposure weakened heterochromatin^12^, likely through relocalization of Dcr1 from the nucleus to the cytoplasm under elevated temperature^70^. SCS strains were grown at 32 °C, shifted to 38 °C overnight, and then returned to 32 °C to monitor the re-establishment of silencing. Both SCS17.5 clones showed only minimal induction of “orange” expression during temperature treatment and rapidly recovered silencing upon return to 32 °C (Fig. 7e). In contrast, the SCS28, SCS33, and SCS37 reporters displayed substantial loss of silencing at 38 °C. Among these, only SCS28-∆VI^1R^ partially restored silencing, whereas the SCS33 and SCS37 strains failed to re-establish detectable repression. Notably, the 28 kb sensor also re-establishes silencing after TSA treatment, indicating that its capacity for heterochromatin recovery is maintained across distinct perturbations. By contrast, the 33 kb and 37 kb sensors, although robustly silenced under steady-state conditions, are highly sensitive to both heat stress and HDAC inhibition.

Finally, to determine whether the impaired recovery of telomere-distal heterochromatin also extends to de novo heterochromatin establishment, we transiently deleted the *clr4* gene and measured the silencing status of both reporters and endogenous subtelomeric genes in SCS33 (clone A) by RT-qPCR (Fig. 7f). Following *clr4* deletion and subsequent re-integration into its endogenous locus using CRISPR, we found that pericentromeric and subtelomeric loci dependent on RNAi quickly re-established silencing, particularly at the *dh* repeats. In contrast, silencing at the “orange” reporter at 33 kb remained incomplete, even 3 weeks after *clr4* re-integration. Analysis of individual subtelomeric genes revealed partial recovery at telomere-proximal loci, whereas more telomere-distal genes located downstream of the reporter insertion site exhibited little or no repression. Together, these findings indicate that de novo heterochromatin establishment is markedly inefficient within the SH domain at telomere-distal loci that are distant from RNAi-dependent nucleation sites.

## Discussion

By deploying a system that tracks a single SH domain in *S. pombe* together with single-cell sensors, we probed both the establishment and epigenetic behavior of SH heterochromatin. Three central findings emerge from this work: (1) Heterochromatin assembles within discrete SH subdomains, possibly through additional cryptic nucleation sites, each exhibiting distinct genetic dependencies and dynamic behaviors; (2) SH heterochromatin displays a spectrum of maintenance profiles across individual cells; (3) SH heterochromatin harbors characteristics that set it apart from constitutive heterochromatin at other genomic loci.

### Formation of SH heterochromatin likely occurs in unique subdomains

Our findings indicate that the genetic requirements for subtelomeric silencing vary substantially across SCS sites, underscoring the position-specific organization of heterochromatin. The RNAi pathway exerts a short-range effect at endogenous SH genes, selectively increasing the expression of the telomere-proximal “orange” reporter, SCS17.5. Shelterin acts over a broader region but has weaker effects on the 28–37 kb reporters and no detectable role near the SH/SU border. Finally, the silencing of SCS28-∆VI^1R^ and SCS37 depends on distinct sets of factors. Notably, silencing of “orange” in SCS28-∆VI^1R^, which mimics the naturally truncated Tel1R arm lacking the 7.1-kb fragment, is restored in RNAi-deficient mutants (*tas3∆, ago1∆*) to levels comparable to the SCS28 sister clone, which retains the complete SH sequence.

We propose two models that can explain these observations: (a) Heterochromatin nucleation occurs exclusively via telomere-proximal RNAi and shelterin pathways, with local chromatin structures modulating silencing efficiency and thereby producing apparently distinct genetic dependencies; or (b) heterochromatin in the SH region is nucleated at multiple, independent sites. In the first model, RNAi or shelterin pathways trigger heterochromatin spreading across the entire SH domain up to the SH/SU boundary. Supporting this, heterochromatin has been shown to spread efficiently over comparable distances when the entire SH region has been removed^21^, implying that spreading can occur independently of SH sequences. Similarly, in budding yeast, functional segregation of the nucleation and spreading pathways suggests that spreading can emerge from a single telomere-proximal site^71^. Model (b), by contrast, would require previously overlooked cryptic nucleation sites internal to SH.

We currently favor this second model for several reasons. First, silencing is maintained at telomere-proximal sites in the absence of both RNAi- and shelterin-dependent nucleation pathways. Second, Clr4 remains associated with distinct, non-coding RNA-expressing loci even in the absence of RNAi and pre-existing H3K9 methylation. This pattern resembles that observed at pericentromeres^18^ and at heterochromatin islands^15,48^, where non-coding RNAs expressed from Clr4 recruitment sites promote heterochromatin nucleation independently of RNAi through the Erh1-Mmi1 complex (EMC). Third, these Clr4 binding sites overlap with persistent H3K9me3 domains in a spreading-deficient *swi6* mutant (Supplementary Fig. S6)^18,45^. Together, these findings support a model in which RNA-dependent but RNAi-independent recruitment of Clr4, potentially mediated by the EMC, enables heterochromatin nucleation within the SH domain.

### Multiple epigenetic states co-exist at the subtelomere

A hallmark of heterochromatin is epigenetic variegation, in which multiple gene expression states can be inherited in a metastable manner. One early example is Telomere Position Effect (TPE), first documented in flies^72^ and later studied extensively in budding yeast^73^. Early studies using artificial minichromosomes^74^ demonstrated TPE in *S. pombe*, and a more recent study showed metastable maintenance of subtelomeric heterochromatin using *ade6* reporters^60^. Our results identify at least three distinct epigenetic behaviors within the subtelomeres: First, the region surrounding 17.5 kb is tightly silenced and rapidly recovers from perturbations. We interpret these observations as evidence that this *tlh2-*proximal site efficiently recruits heterochromatin assembly factors, thereby conferring a chromatin state that is highly resistant to perturbation. This behavior is consistent with robust nucleosome assembly, high occupancy, and low turnover^69,75,76^. Second, “orange” in SCS28-∆VI^1R^ behaves in a manner most consistent with metastable epigenetic inheritance. Silencing at this site can adopt multiple states that are stably propagated across generations, and perturbations regenerate the same clonal distribution observed during initial reporter characterization. This type of metastable behavior is somewhat reminiscent of PEV in flies^77^. The observation that the 7.1-kb fragment missing in this reporter is also absent in the natural Tel1R structural variant suggests that this region frequently undergoes recombination. It further implies that different epigenetic states on individual chromosomal arms may co-exist, even in strains with native SH sequences. Finally, the more distal reporters in SCS33 and SCS37 show strong silencing under steady-state conditions but lose it completely after perturbation, with no recovery even after a month or upon *clr4* reintroduction. This indicates a highly fragile epigenetic state: silencing is stably maintained once established but can hardly be re-established after disruption. A similar pattern occurs at the MAT locus upon RNAi loss^68^ where silencing remains stable but fails to recover after perturbation^12,17,78^. This has been attributed to the inability of Clr4 to nucleate in the absence of the RNAi machinery, even though maintenance remains intact. A comparable mechanism may operate near the 33-kb and 37-kb regions, where nucleation or spreading factors may act infrequently, transiently, or fail to rebind acetylated chromatin.

### Unique aspects of SH heterochromatin point to a more euchromatic and unstable chromatin environment

When comparing subtelomeres with other heterochromatin loci, several aspects of SH heterochromatin stand out. One is their dynamic behavior, which occupies an intermediate position between the stable MAT locus and the highly unstable states of RNAi-driven domains^12,79^. At SCS28-∆VI^1R^ and SCS28, we observe infrequent ON ⟷ OFF fluctuations that appear like switches between metastable states. Their detection in even a small cell population suggests switching rates much higher than those of classic bistable systems such as *MAT∆K*, which occur at 10^−4^ per generation^80^. These findings place SH heterochromatin in an intermediate, metastable category, consistent with previous reports.

Moreover, the genetic requirements of SH-heterochromatin more closely resemble those of ectopically assembled heterochromatin in euchromatin than those of constitutive heterochromatin. Specifically, we previously showed that heterochromatin spreading in euchromatin depends on the HIRA histone chaperone and the histone variant H2A.Z-depositing Swr1 complex, while being strongly antagonized by chromatin remodelers^49^. A similar pattern emerges for SCS28-∆VI^1R^ and SCS37, although here remodeler subunits can either promote or oppose silencing. We interpret this as evidence of greater nucleosome instability than at constitutive heterochromatin sites, consistent with a more euchromatin-like environment. Another parallel with ectopic spreading is sensitivity to H3K4 methylation: H3K4me3 deposited by Set1 acts as a barrier to spreading when heterochromatin boundaries are removed^13^. At SH heterochromatin, loss of the H3K4me3 demethylase Jmj2^53^ enhances silencing of SCS28-∆VI^1R^, which suggests that failure to remove H3K4 methylation imposes a barrier to spreading. Since SH heterochromatin domains lack defined DNA-encoded boundaries^19,81^, such epigenetic barriers may redirect H3K9 methylation to internal sites such as the 28-kb region, thereby enhancing silencing.

Finally, SH heterochromatin competes with other heterochromatin domains for limiting silencing factors. This is evident from our observation that RNAi mutants enhance silencing at telomere-distal sites in strains lacking the common block VI or V, indicating that factors released from RNAi-dependent heterochromatin sites elsewhere are redirected to SH heterochromatin. Further, our genetic screen uniquely identified chromatin remodelers that promote silencing at SCS28-∆VI^1R^ and SCS37, in contrast to their usual antagonistic role at other heterochromatin regions^49,54^. We propose that, in their absence, silencing is strengthened at constitutive heterochromatin sites, thereby diverting limiting factors away from SH regions. In line with this model, we observed that RNAi- and shelterin-dependent silencing is concentrated at telomere-proximal regions, but that silencing profiles become more uniform across SH in their absence. Such spatial buffering may stabilize silencing at internal SH sites while preventing heterochromatin from spreading into the adjacent SU domain, thereby protecting stress-responsive genes from inappropriate silencing^21^.

SH heterochromatin, while divergent in sequence across species, has globally conserved features such as its “mosaic” and repetitive nature and lack of defined boundaries, representing an example of “convergent” chromosome evolution. In *S. pombe*, we show that SH heterochromatin harbors multiple, epigenetically distinct behaviors. These differences are not dictated by the overall presence of repressive H3K9 methylation but rather reflect variation in histone acetylation and the activity of yet-to-be-defined cryptic nucleators that operate independently of RNAi or shelterin. Notably, subtelomeres are hotspots of structural variation and genome innovation, with similar alterations reported at individual subtelomeric loci in humans. Whether similar epigenetic fragmentation of subtelomeric heterochromatin occurs in other eukaryotes remains an open question. Elucidating how these position-specific silencing behaviors influence genome stability, gene regulation, and long-term adaptation will be a key goal for future studies.

## Methods

### Yeast strains and media

All *S. pombe* strains, plasmids and oligonucleotides used in this study are listed in tables in the Supplementary Data S1, S2, and S3, respectively. Gene deletions were generated by standard genome engineering procedures using transformation with polymerase chain reaction (PCR) products and genomic integration via homologous recombination. Generated strains were validated by colony PCR. Reporter strains for monitoring silencing by flow cytometry (FC) were generated by sequentially inserting three transcriptionally encoded fluorescent reporters, as described by Greenstein et al.^12^, into the subtelomeric regions of chromosome arm IIR in the *SD4[2R*+*]* strain from Junko Kanoh’s laboratory^21^ using the CRISPR/Cas9-based SpEDIT method^82^.

Superfolder GFP (SF-GFPs.p., “green”) driven by the ade6 promoter was integrated proximal to the tlh2 gene approximately 11 kb downstream of the telomeric repeats; ade6p-driven Kusabira orange (mKO2s.p., “orange”) was integrated at 17.5, 28, 33 or 37 kb from the telomeric repeats; act1p-driven E2Crimson (E2Cs.p., “red”) was inserted at the nearest euchromatic region ∼46.5 kb (note: subtelomeric positions are corrected for the ∼4 kb sequence missing at the end of chromosome IIR in the annotated chromosome sequence on www.pombase.org). For each of the four “orange” reporter insertion sites (17.5, 28, 33, or 37 kb), two biologically independent clones (A and B) were analyzed. Additional mutations (e.g., *tas3∆*, *taz1∆*) were introduced into these reporter strains by homologous recombination, except for *clr4* deletions, which were introduced prior to the insertion of the “green” and “orange” reporters.

For reverse transcriptase combined with quantitative PCR (RT-qPCR), chromatin immunoprecipitation (ChIP)-qPCR and flow-cytometry experiments, cells were grown in rich medium (Yeast Extract Supplemented, YES) at 32 °C.

### RNA extraction and cDNA synthesis

RNA extraction and cDNA synthesis were carried out as previously described^62,64,83^: Yeast cultures (50 ml, OD₆₀₀ = 0.4–0.8) were harvested at 4 °C and frozen in liquid nitrogen. Cell pellets were resuspended in Trizol with zirconia beads and lysed by bead beating (4 × 30 s, 5 min rests on ice). After centrifugation, RNA was extracted twice with chloroform, precipitated using isopropanol, washed twice with 75% ethanol, air-dried, and resuspended in 60 μl RNase-free water. Concentration and purity were assessed with a NanoDrop™ spectrophotometer. A 20 μg aliquot of RNA was treated with DNaseI (Ambion) for 1 h at 37 °C, and the reaction was stopped with 6 μl of inactivation reagent. For cDNA synthesis, 5 μg of DNase-treated RNA was reverse-transcribed using oligo(dT)20 primers (50 μM) and 0.25 μl SuperScript IV (Invitrogen), following the manufacturer’s instructions. cDNA was quantified by real-time PCR using PowerTrack™ SYBR Green Mastermix (Applied Biosystems™) on a QuantStudio 5 Real-Time PCR System. Primers used for qPCR are listed in Supplementary Data S3. Transcript levels were normalized to *act1* and further processed as described in the corresponding Figure Legends and Source File.

### Chromatin immunoprecipitation (ChIP)

ChIP-qPCR was performed as described previously^62,64,84^: Yeast cultures (OD₆₀₀  = 0.6–0.8) were cross-linked with 1% formaldehyde for 10 min and quenched with 150 mM glycine. Cells were washed twice with 50 ml ice-cold PBS (phosphate-buffered saline), and cell pellets were frozen in liquid nitrogen. Cells were lysed in ice-cold lysis buffer A (50 mM HEPES-KOH pH 7.5, 140 mM NaCl, 1 mM EDTA, 1% Triton X-100, 0.1% sodium deoxycholate) containing protease inhibitors and zirconia beads. Chromatin was sheared by sonication (30 s on/off cycles, 30 min, 4 °C) and incubated overnight with antibodies (2 µg per IP sample) against H3K9me2 (Abcam ab1220), H3K9me3 (Active Motif 39161), H3K14ac (Abcam ab52946), or H4K16ac (Active Motif 39167), followed by capture with Dynabeads Protein G. Beads were washed, de-crosslinked, treated with proteinase K, and DNA was purified using a ChIP DNA Clean and Concentrator kit (Zymo Research). Input (1:100) and IP (1:20) DNA samples were quantified by real-time PCR using PowerTrack™ SYBR Green Mastermix (Applied Biosystems™) on a QuantStudio 5 system. Primers used for qPCR are listed in (Supplementary Data S3). IP signals were normalized to input and further processed as described in the corresponding Figure Legends and Source Data.

### Flow cytometry

For gene expression analysis in single cells, flow cytometry (FC) analysis was performed according to a previously described protocol^12^. *S. pombe* cells were grown to stationary phase in YES media and then diluted to a concentration of OD600 = 0.1 in YES, followed by incubation at 32 °C for 4–5 h prior to FC analysis. The Fortessa X-50 instrument (BD), equipped with a high-throughput sampler (HTS) module, was employed for flow cytometry analysis. Sample sizes ranged from approximately 2000 to 100,000 cells, depending on the growth conditions of the respective strain. Compensation was performed using strains expressing no fluorescent proteins (XFP) and a single-color control XFP (SF-GFPsp (“green”), mKO2sp (“orange”) or E2Csp (“Red”)). Compensated “green” and “orange” signals were normalized to (“red”) expressed from a euchromatic control locus in every single cell. Additionally, a maximum expression value for “green” and “orange” was set based on their expression in a heterochromatin-deficient control strain (*clr4Δ*), where reporters should be in an ON state. However, since the reporters are inserted at heterochromatin domains, which are prone to recombination in *clr4Δ* strains, there is a risk of losing these reporters. To overcome this issue, color-negative cells were excluded by setting a minimum cutoff for “green” and “orange” This threshold was determined using a control strain expressing only the “red” reporter, which represents a fully repressed state for both “green” and “orange”. Specifically, the “OFF threshold” was calculated as the median fluorescence intensity of the “red”-only control plus 15 times the median absolute deviation (MAD). “Max” expression values for “green” and “orange” were then calculated from these color-positive cells in the no-heterochromatin control strain. Subsequently, “red” normalized “green” and “orange” signals were scaled to the corresponding “max” values in our analysis strains, and the scaled, normalized signals were plotted in 2D hexbin or density plots for visualization. To estimate the fraction of fully nucleated cells, cells falling below the defined “green” and “orange” OFF thresholds were gated and isolated. The fraction of these fully repressed cells was then calculated and reported as the OFF percentage in the corresponding hexbin plots.

### Flow cytometry-based screen

Our curated chromatin knock-out collection comprises a subset of chromatin function mutants from the *S. pombe* BIONEER (ver3) gene deletion library. SCS reporters were crossed into this library as previously described^49,62^. Knock-out strains were validated in this and our previous study^62^ using deletion-specific barcode sequences published by Han et al.^85^ together with genomic PCR analysis. Validation results are shown in Supplementary Data S4–S6.

Scaled and normalized fluorescence intensity values for the “orange” reporter, as described above, were stratified into four discrete expression zones (Zones 1–4) using custom-defined thresholds along the *Y*-axis. These thresholds included an experimentally determined “OFF threshold” (orange^OFF^), 0.4 and 0.8. The orange^OFF^ threshold was established based on a control strain expressing only the “red” reporter, which represents a fully repressed baseline state for both “green” and “orange” reporters. The “OFF threshold” was calculated as the median fluorescence intensity of the “red”-only control plus 8 times the MAD. Cells were assigned to one of four zones corresponding to increasing levels of “orange” expression: OFF (Zone 1), mid-low (Zone 2), mid-high (Zone 3), and ON (Zone 4). Samples containing fewer than 1000 cells were excluded from further analysis. The fractions of cells within each zone were calculated for each sample, and the data were visualized as stacked bar plots to represent the distribution of “orange” expression states.

Mutants were clustered based on fractions obtained from zone analysis using k-means clustering. The optimal number of clusters (k) was determined in Python, with data handling performed using NumPy v2.2.6 and pandas v2.3.0. Within-cluster sum of squares (WCSS) and silhouette scores were calculated across a range of *k* values with scikit-learn v1.6.1 and visualized using matplotlib v3.10.3. The KneeLocator method from kneed v0.8.5 was used to identify the optimal k according to the elbow method.

### Flow cytometry activated cell sorting (FACS)

Cells were grown overnight in 3 ml YES media and then diluted in the morning to OD = 0.1 in YES and grown for 5–6 h (OD = 0.4). Cells from 2 mL of culture were pelleted and washed once with ice-cold FACS buffer (1XPBS, 1% BSA, and 5 mM EDTA). Cells were resuspended in 500 µl of ice-cold FACS buffer and passed through a 35–40 μm cell-strainer. An additional 1.5 mL cold FACS Buffer was added to the strainer to wash any trapped cells into the collection tube. Sorting was performed using FACSAria2 (Becton Dickinson). Cells considered for sorting had to pass selection for (1) Young, primarily singlet G2 cells, using a size gate determined by forward (FSC) and side (SSC) scatter, as described in Greenstein et al.^12^, and (2) intact heterochromatin at 17.5 kb (“green”^OFF^), excluding all cells above the range of an unstained control. Cells that passed this double filter were then gated into “orange”^OFF^, and “orange”^HIGH^ signal defined by the following: OFF encompassed signal overlapping that of an unstained control, and HIGH encompassed signal overlapping that of the “orange” only control strain PAS52, which carries *ade6p:*mKO2 at a euchromatic locus^12^. Approximately 8 × 10^6^ cells from each subpopulation were collected. Immediately after sorting, cells were divided into two thirds for ChIP experiments and one third for RT-qPCR, and were subjected to the appropriate fixation treatment for either experiment. ChIP experiments were performed as previously described^12^.

### Fission yeast lifespan micro-dissector (FYLM)

FYLM experiments were performed as previously described^12^. Fluorescence was quantified as the mean intensity within a region of interest. For each fluorophore (SF-GFPsp, mKOsp, and E2Csp), an empty catch tube was used to measure autofluorescence across the entire experiment. The autofluorescence was subtracted from the fluorescence traces for each cell at each frame and the “green” and “orange” to “red” ratios were calculated from these background-subtracted values. A *clr4*∆ mutant was included in each experiment to define the ON/OFF state threshold for each fluorophore ratio. The threshold for ON was defined as mean minus three standard deviations of the maximum ratio attained by *clr4*∆ cells. In Fig. 4, ratios were normalized to the mean of the maximum ratios attained by each *clr4*Δ mutant cell within each experiment.

### Heat recovery assay

Freshly plated cells were inoculated into 96-well plates containing 200 µL of YES medium per well and incubated overnight with shaking (Elmi) at either 32 °C or 38 °C (Day −1). The next morning, cultures were diluted into fresh YES medium (200 µL) and maintained at the same temperature for approximately 6 h before being analyzed by flow cytometry (Day 0). Following this measurement, cultures were diluted again into YES medium and incubated overnight at 32 °C. On Day 1, cells from the overnight cultures were re-diluted into YES medium at 32 °C, grown for 6 h, analyzed by flow cytometry, and returned to the same temperature for overnight growth. This cycle was repeated for three additional days (Days 2–4).

### Trichostatin A (TSA) recovery assay

Cells from freshly streaked plates were inoculated into 96-well plates containing 200 μL YES medium supplemented with 30 μM TSA and incubated overnight with shaking (Elmi) (Day −1). On the following day (Day 0), cultures were diluted into fresh YES medium containing 30 μM TSA and analyzed by flow cytometry. Subsequently, cells were plated onto YES agar and incubated at 32 °C for 2–3 days to allow recovery. Single colonies were picked, patched onto YES plates, and incubated for an additional 2–3 days at 32 °C. These isolates were maintained on YES plates for a total of 12 days. On Day 12, cultures grown overnight in 96-well plates containing 200 μL YES were diluted into fresh YES medium, incubated for approximately 6 h, and analyzed by flow cytometry. Isolates were continuously maintained on YES plates, and the same growth and measurement procedure was repeated on Days 19 and 28.

### Reporting summary

Further information on research design is available in the Nature Portfolio Reporting Summary linked to this article.

## Supplementary information

Supplementary Information

Description of Additional Supplementary Files

Supplementary Data 1-6

Reporting Summary

Transparent Peer Review file

## Source data

Source Data

## Data Availability

Source data are provided with this paper. The Flow cytometry raw data generated in this study have been deposited on Zenodo under accession number 21360361 https://doi.org/10.5281/zenodo.21360361. The processed flow cytometry data are also available on Zenodo. The RT PCR and ChIP data generated in this study are provided in the Source Data File. All *S. pombe* strains generated for and used in this study are listed in Supplementary Data S1 and are available upon request. Source data are provided with this paper.

## Code availability

Flow cytometry scripts for their analysis have been deposited on Zenodo under the accession number 21360361 https://doi.org/10.5281/zenodo.21360361.
