# Supplementary material for: Discrete subdomains establish epigenetic diversity in subtelomeric heterochromatin in fission yeast
Source: Nat Commun. 2026 Oct 12;17:11564. doi: 10.1038/s41467-026-78445-5 (PMC13748586; doi:10.1038/s41467-026-78445-5)
Supplement: Supplementary file 1 [file 41467_2026_78445_MOESM1_ESM.pdf]

Supplementary Information

Discrete Subdomains Establish Epigenetic Diversity in Subtelomeric Heterochromatin in Fission Yeast

Agnisrota Mazumder, John Cooper, Can Goksal, Rosario Y. J. Brockhausen, Jonas M. Schauß, Jasbeer S. Khanduja, Richard I. Joh, Ahmed A. A. Amine, Johannes J. Groos, Junko Kanoh, Mo Motamedi, Ilya J. Finkelstein, Bassem Al-Sady[‡], and Sigurd Braun[‡]

Supplementary Figures S1-S8

[‡] Corresponding authors

Bassem.Al-Sady@ucsf.edu
Sigurd.Braun@gen.bio.uni-giessen.de

Supplementary Figures

a Similarity matrix SH genes (tel2R)

	SPBCPT2R1.04c	SPBCPT2R1.01c	SPBCPT2R1.05c	SPBPB2B2.14c	SPBCPT2R1.03	SPBCPT2R1.06c	ftm7	SPBCPT2R1.07c	SPBCPT2R1.10	SPBPB2B2.19c	SPBPB2B2.16c	SPBCPT2R1.02	SPBPB2B2.18	mean	max
SPBCPT2R1.04c		99.7	79.3	80.4	45.6	39.5	34.2	38.9	47.4	44.5	39.1	39.8	40.5	52	100
SPBCPT2R1.01c	99.7		79.3	81.8	46.8	37.5	33.6	39.7	47.6	44.0	39.8	40.4	40.8	53	100
SPBCPT2R1.05c	79.3	79.3		83.7	57.1	36.9	33.3	40.6	40.7	38.1	39.8	40.0	39.7	51	84
SPBPB2B2.14c	80.4	81.8	83.7		46.5	37.4	32.5	37.7	43.3	42.4	39.0	42.3	41.3	51	84
SPBCPT2R1.03	45.6	46.8	57.1	46.5		44.0	35.4	34.9	40.5	44.6	44.3	31.4	33.5	42	57
SPBCPT2R1.06c	39.5	37.5	36.9	37.4	44.0		51.7	37.2	37.8	42.1	38.6	37.1	40.0	40	52
ftm7	34.2	33.6	33.3	32.5	35.4	51.7		50.6	36.2	32.7	33.3	30.0	36.2	37	52
SPBCPT2R1.07c	38.9	39.7	40.6	37.7	34.9	37.2	50.6		35.0	36.6	38.3	30.9	41.1	38	51
SPBCPT2R1.10	47.4	47.6	40.7	43.3	40.5	37.8	36.2	35.0		40.7	40.2	41.0	35.7	41	48
SPBPB2B2.19c	44.5	44.0	38.1	42.4	44.6	42.1	32.7	36.6	40.7		40.4	40.2	42.4	41	45
SPBPB2B2.16c	39.1	39.8	39.8	39.0	44.3	38.6	33.3	38.3	40.2	40.4		43.1	43.1	40	44
SPBCPT2R1.02	39.8	40.4	40.0	42.3	31.4	37.1	30.0	30.9	41.0	40.2	43.1		32.8	37	43
SPBPB2B2.18	40.5	40.8	39.7	41.3	33.5	40.0	36.2	41.1	35.7	42.4	43.1	32.8		39	43
mean	52.4	48.3	48.1	48.0	41.7	40.0	36.9	38.4	39.9	40.4	40.0	37.2	38.8		

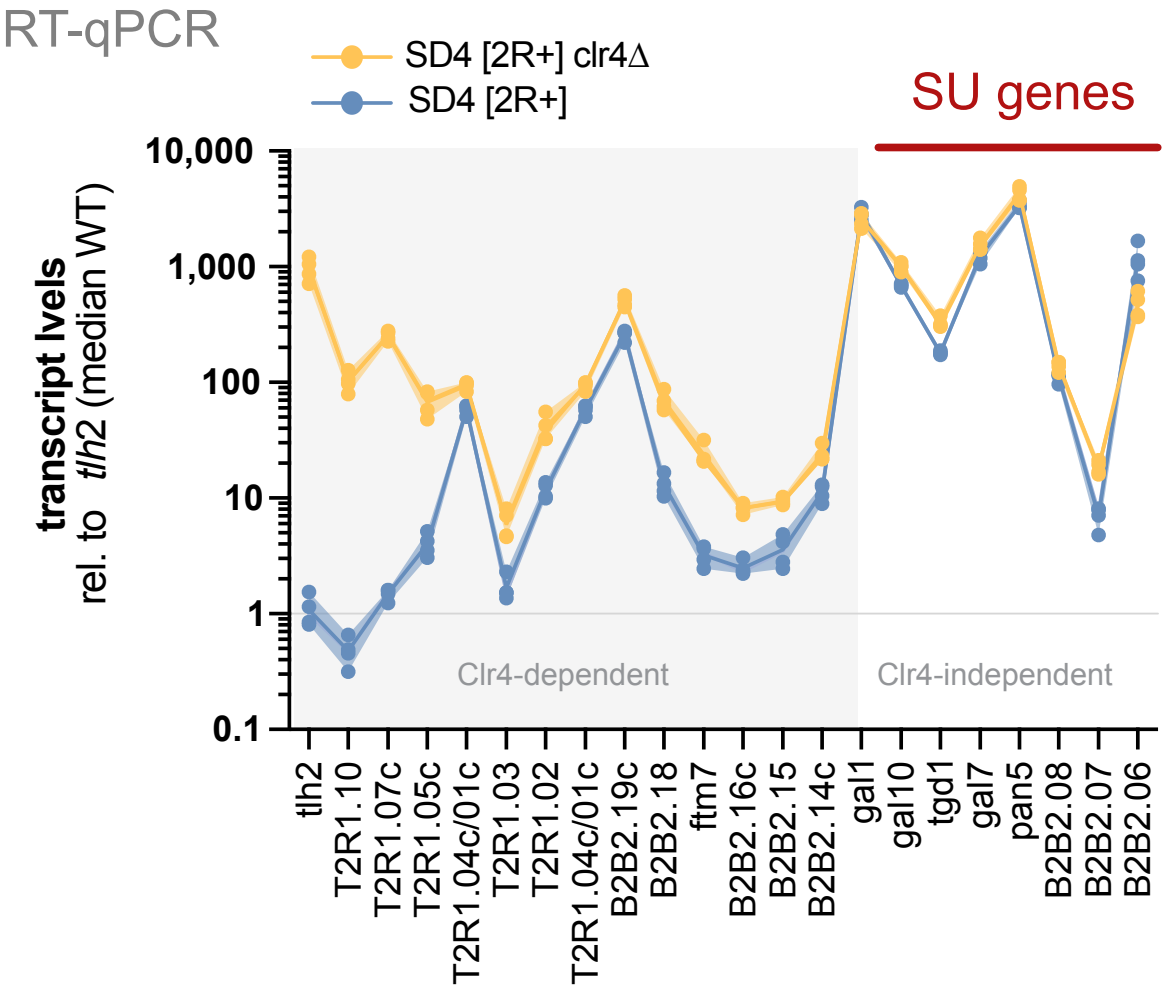

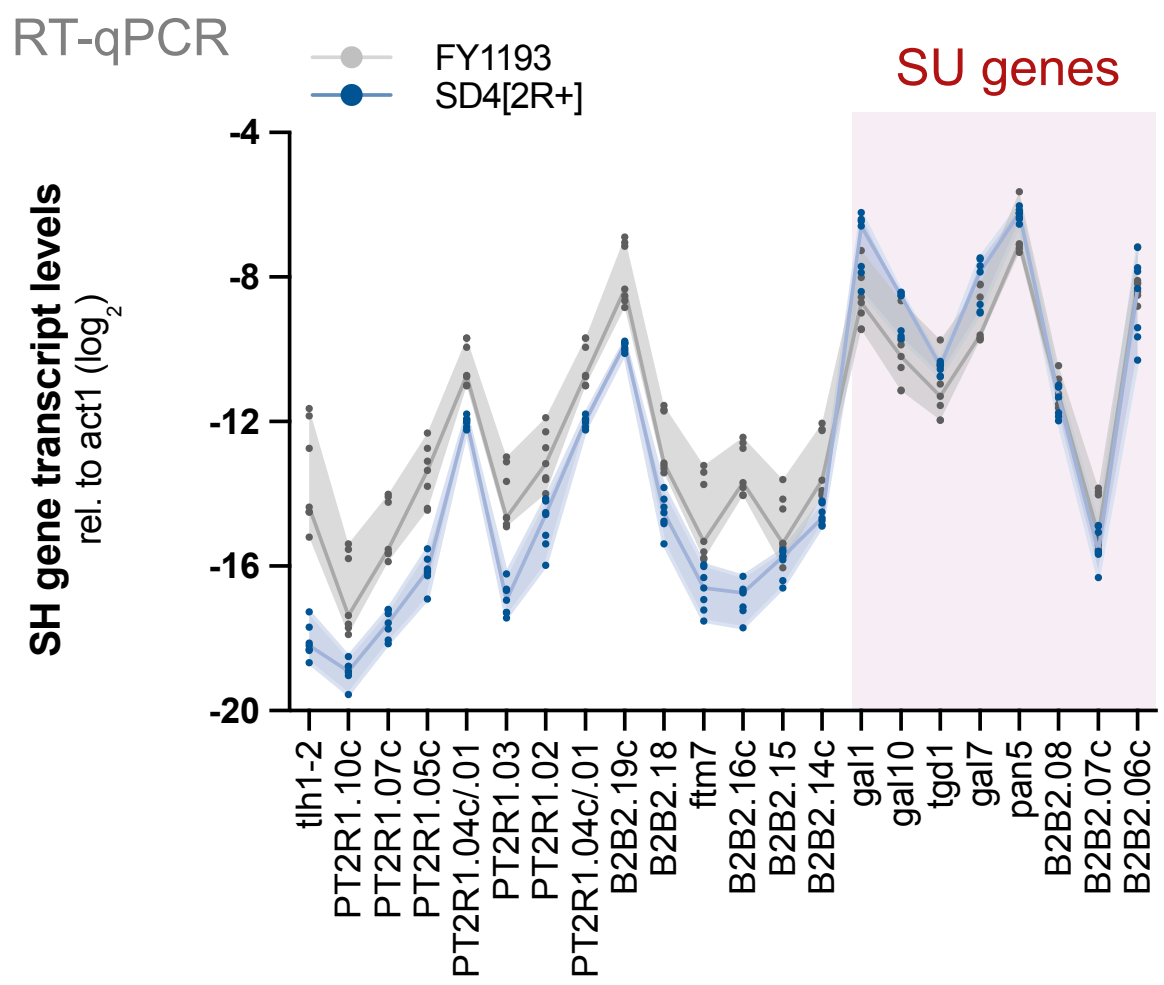

Suppl. Figure S1: SH gene expression patterns are not impacted by the total number of subtelomeres.

(a) Similarity matrix of SH-region genes on chromosome arm Tel2R. The matrix displays pairwise nucleotide sequence similarity (%) among SH genes. Rows and columns represent individual genes. The two right columns display for each gene the mean and maximum similarity values, respectively, of all pairwise gene comparisons. The bottom row also shows the mean similarity for each gene.

(b) Clr4-dependence of SH and SU gene expression the *SD4[2R+]* strain. RT-qPCR analysis of SH and SU genes in the wild-type (blue) and *clr4Δ* (yellow) *SD4[2R+]* background. Transcripts were normalized to *act1* and are shown relative to the median *tlh2.* expression level in WT samples. Independent biological replicates (n = 4) are shown as individual data points with median and range.

(c) RT-qPCR analysis of SH and SU gene transcript levels in the *SD4[2R+]* (blue) and FY1193 (grey). FY1193 (Ekwall et al. 1999) is derived from laboratory strain 972 and retains all native subtelomeric arms. Transcript levels were normalized using strain-specific genomic DNA to account to account for SH copy-number differences and subsequently to *act1* (log_2 scale). Independent biological replicates (n = 7) are shown as individual data points with median and range.

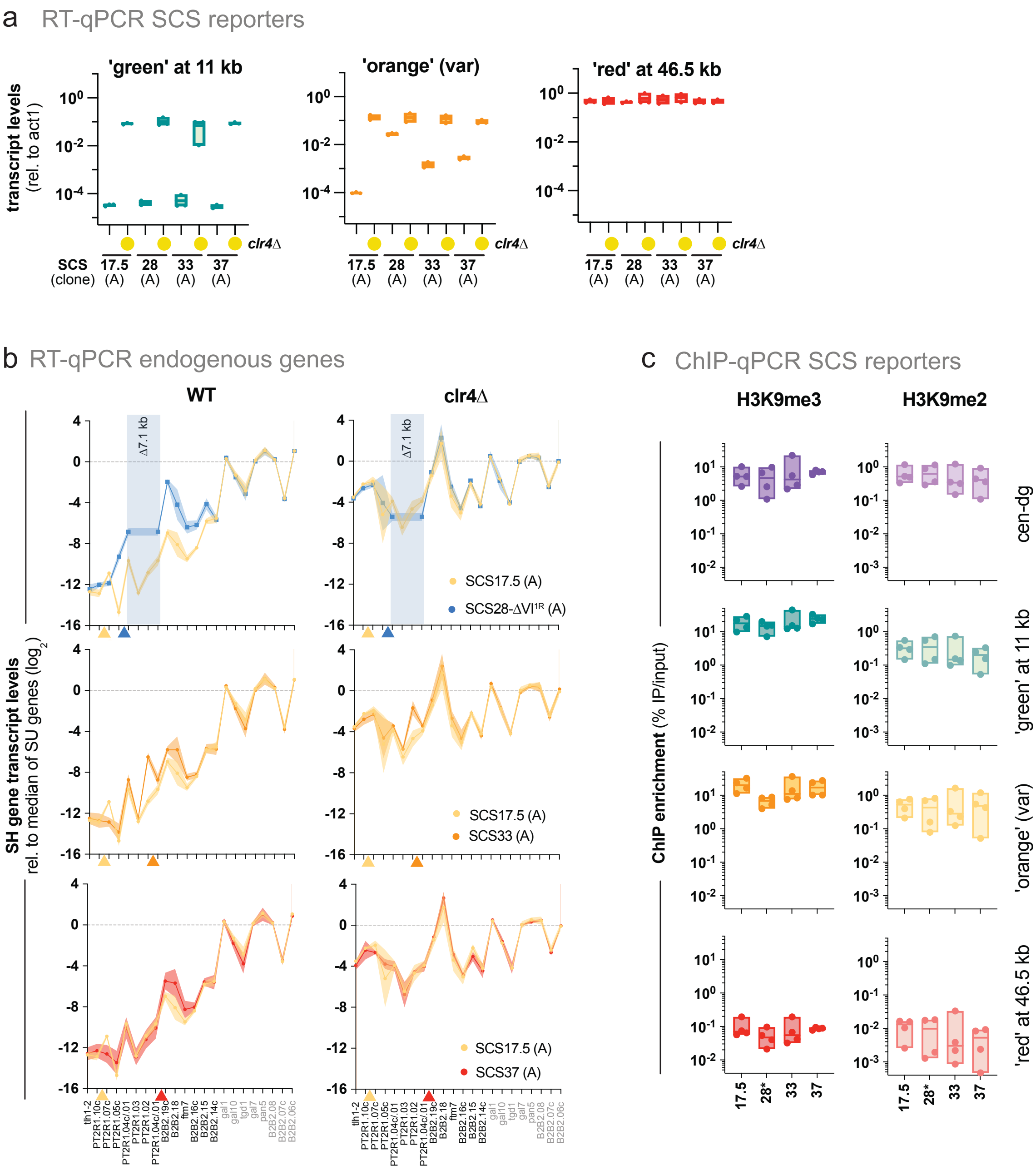

Supplementary Figure S2: Characterization of gene silencing and genetic architecture of single-cell reporter strains.

(a) RT-qPCR analysis of reporter transcript levels in wild-type and *clr4*Δ strains across all SCS constructs (clone A). Transcript levels of 'green' (11 kb), 'orange' (17.5, 28, 33, and 37 kb), and 'red' (46.5 kb) reporters were normalized to *act1*. Individual data points represent independent biological replicates (n = 3).

(b) RT-qPCR analysis of SH and SU gene expression in SCS17.5 (yellow) compared with SCS28-ΔVI^{1R} (blue; top), SCS33 (orange; middle), and SCS37 (red; bottom) in wild-type (left) and *clr4*Δ (right) backgrounds (clone A). Transcript levels were normalized to *act1* and are shown relative to median SU-region expression ($\log_2$ scale). Independent biological replicates: WT SCS17.5, n = 14; WT SCS28-ΔVI^{1R}, n = 15; WT SCS33, n = 11; WT SCS37, n = 9; *clr4*Δ strains, n = 4 for each strain. Data are shown as mean (line) with 95% confidence interval (shaded area). The schematic shows SH and SU gene position; the ~7.1-kb region absent from SCS28-ΔVI^{1R} is shaded in blue.

(c) ChIP-qPCR for H3K9me3 (left) and H3K9me2 (right) enrichment, shown as IP/input without additional normalization (cf. Figure 2e). Panels show, from top to bottom, cen *dh-dg*, the 'green', 'orange', and 'red'. Independent biological replicates (n = 4) are shown as individual data points with median and range.

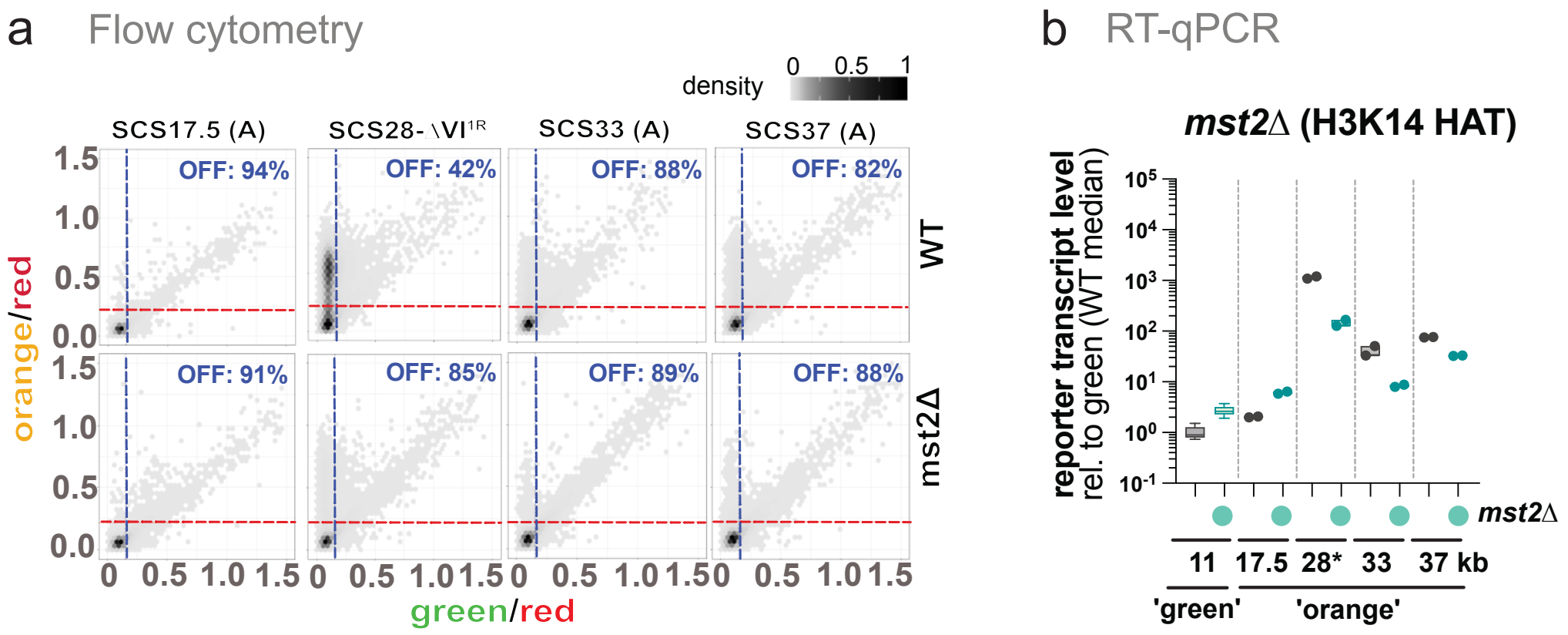

Supplementary Figure S3: Dependence of SCS reporter silencing on Mst2.

(a) Two-dimensional hexbin density plots showing red-normalized 'green' (x-axis) and 'orange' (y-axis) fluorescence for SCS reporters in WT (top) and *mst2Δ* cells (bottom). Blue and red dashed lines indicate OFF thresholds for green and orange reporters, respectively.

(b) RT-qPCR analysis of reporter transcript levels in WT and *mst2Δ* cells. Transcript levels of the 'green' reporter (11 kb) and 'orange' reporters (17.5, 28-ΔVI^{1R}, 33, and 37 kb) were measured in wild-type (grey) and *mst2Δ* (green) strains. Transcript levels were normalized to *act1* and are shown relative to the median 'green' reporter expression in WT. For 'green' reporters, independent biological replicates ($n = 8$) are shown as Tukey box-and-whisker plots; boxes show the median and interquartile range, and whiskers extend to the most extreme values within 1.5× the interquartile range. For each 'orange' reporter, individual data points represent independent biological replicates ($n = 2$).

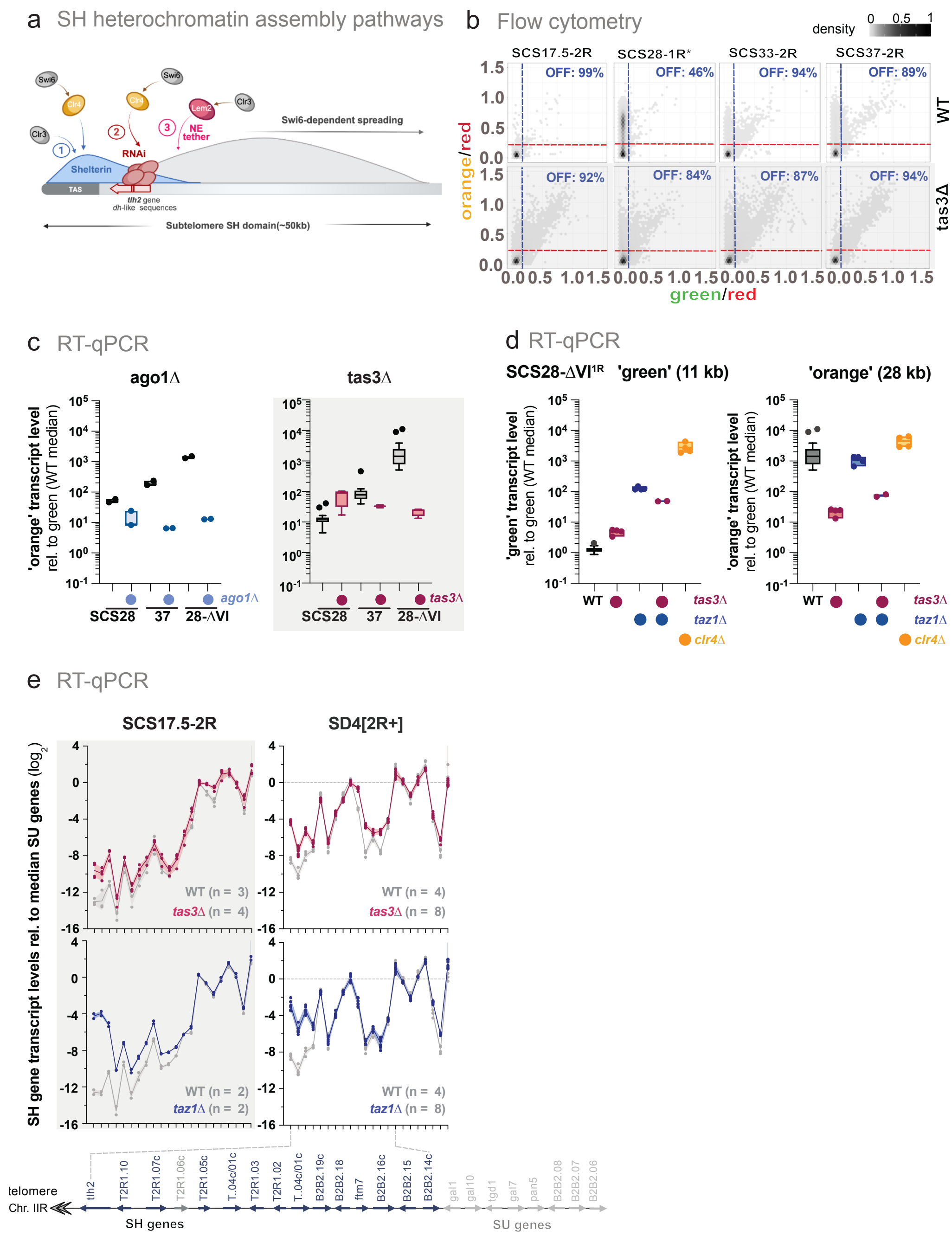

Supplementary Figure S4: Dependence of SCS reporter silencing on RNAi and shelterin pathways.

(a) Schematic representation of heterochromatin nucleation pathways at the telomeric and subtelomeric SH region (~50 kb). The diagram illustrates the shelterin-dependent pathway (blue), RNAi-mediated pathway (red), and Lem2-dependent nuclear tethering pathway (pink). Heterochromatin spreading is indicated as Swi6-dependent. Created in BioRender. Braun, S. (2026) https://BioRender.com/flokio9.

(b) Two-dimensional hexbin density plots of WT and *tas3Δ* cells. The shaded area reproduces data from Figure 2; see Figure 2 legend for plot definitions and details.

(c) RT-qPCR analysis of 'orange' transcript levels in *ago1Δ.* Left: 'orange' transcript levels in wild-type and *ago1Δ* SCS28, SCS37 and SCS28-ΔVI^{1R} strains. Transcript levels were normalized to *act1* and are shown relative to median 'green' reporter expression in WT. Individual data points represent independent biological replicates (n = 2). The shaded panel reproduces the corresponding WT and *tas3Δ* data from Figure 3b.

(d) RT-qPCR analysis of 'green' and 'orange' reporter transcripts in SCS28-ΔVI^{1R} in the indicated genetic backgrounds. Independent biological replicates: WT, n = 16; *tas3Δ*, n = 4; *taz1Δ*, n = 4; *tas3Δ taz1Δ*, n = 2; *clr4Δ*, n = 4). For $n > 4$ (WT samples), Tukey box-and-whisker plots show the median and interquartile range; whiskers extend to the most extreme values within 1.5× the interquartile range. For $n \leq 4$, individual data points are shown with median and range. Single-mutant data are reproduced from Figure 3b.

(e) RT-qPCR analysis of transcript levels across the SH and SU regions in SCS17.5-2R (left) and *SD4[2R+]* (right) strain backgrounds. Transcript levels were normalized to *act1* and are shown relative to median SH expression ($\log_2$ scale). Independent biological replicates are shown with median (lines) and range (shaded areas). The precise number of biological replicates (n) is indicated in each plot. The schematic shows the organization of SH and SU genes. In the left panel, the light grey shading indicates data reproduced from Figure 3c.

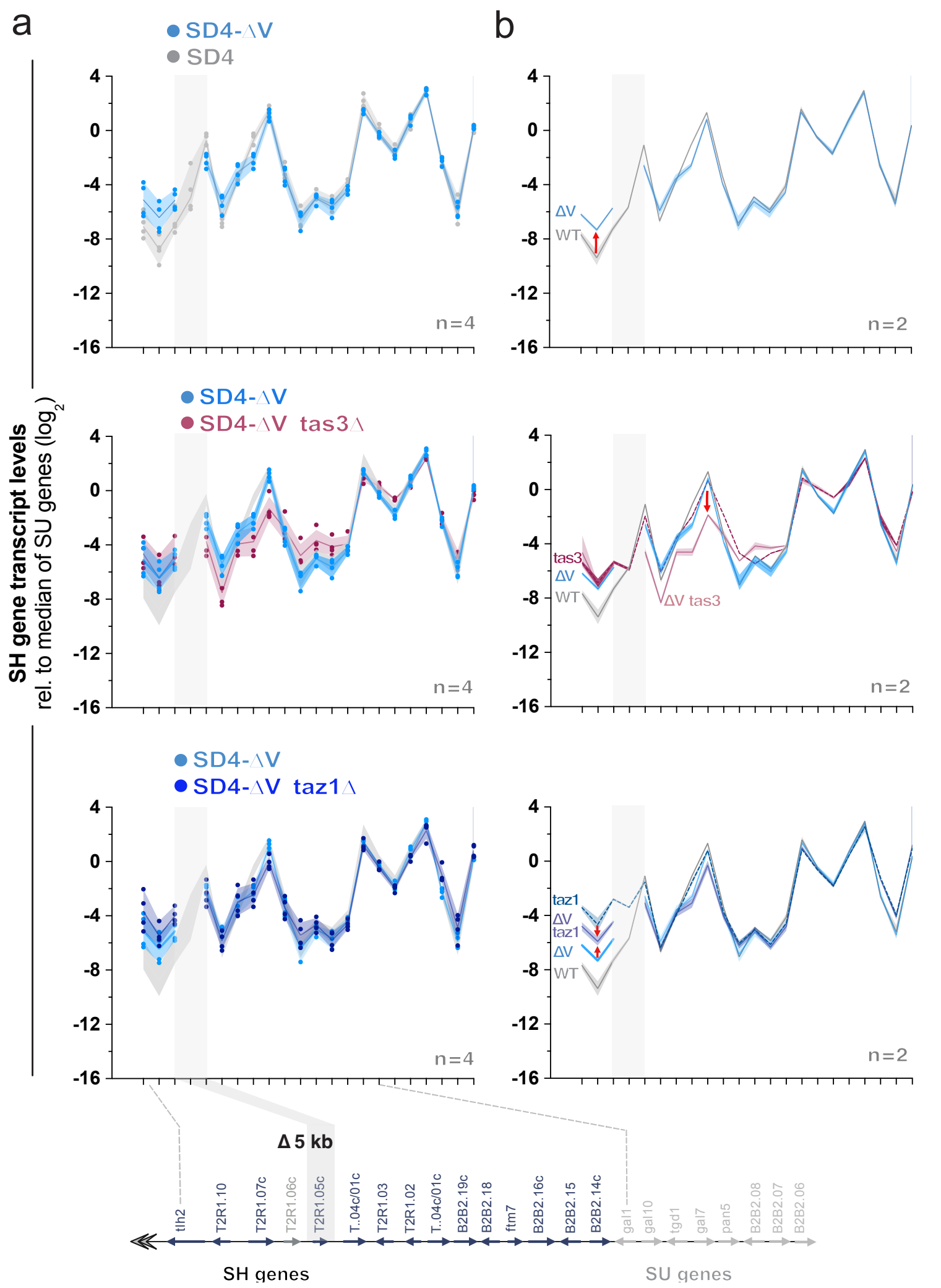

Supplementary Figure S5: Block V locally control gene silencing at the subtelomere in the parental *SD4[2R⁺]* strain

(a, b) RT-qPCR analysis of the effect of the 5-kb block V deletion on endogenous subtelomeric gene expression in the parental *SD4[2R⁺]* strain lacking reporter insertions. Top: *SD4[2R⁺]* WT (grey) versus *SD4[2R⁺]* ΔV (light blue). Middle: *SD4[2R⁺]* ΔV (light blue) versus *SD4[2R⁺]* ΔV *tas3*Δ (dark red). Bottom: *SD4[2R⁺]* ΔV (light blue) versus *SD4[2R⁺]* ΔV *taz*Δ (dark blue). Transcript levels were normalized to *act1* and are shown relative to the median SU-region expression (log_2 scale). For (a), biological replicates (n = 4) from two independent experiments are shown as individual data points with the mean and range. For (b), biological replicates (n = 2) from one experiment are shown as mean and range. WT and ΔV data are reproduced from (a). The middle and bottom panels in (b) additionally show the corresponding *tas3*Δ and *taz1*Δ single mutants lacking the block V deletion (dotted lines). Red arrows indicate loci with substantial expression differences between the corresponding strains.

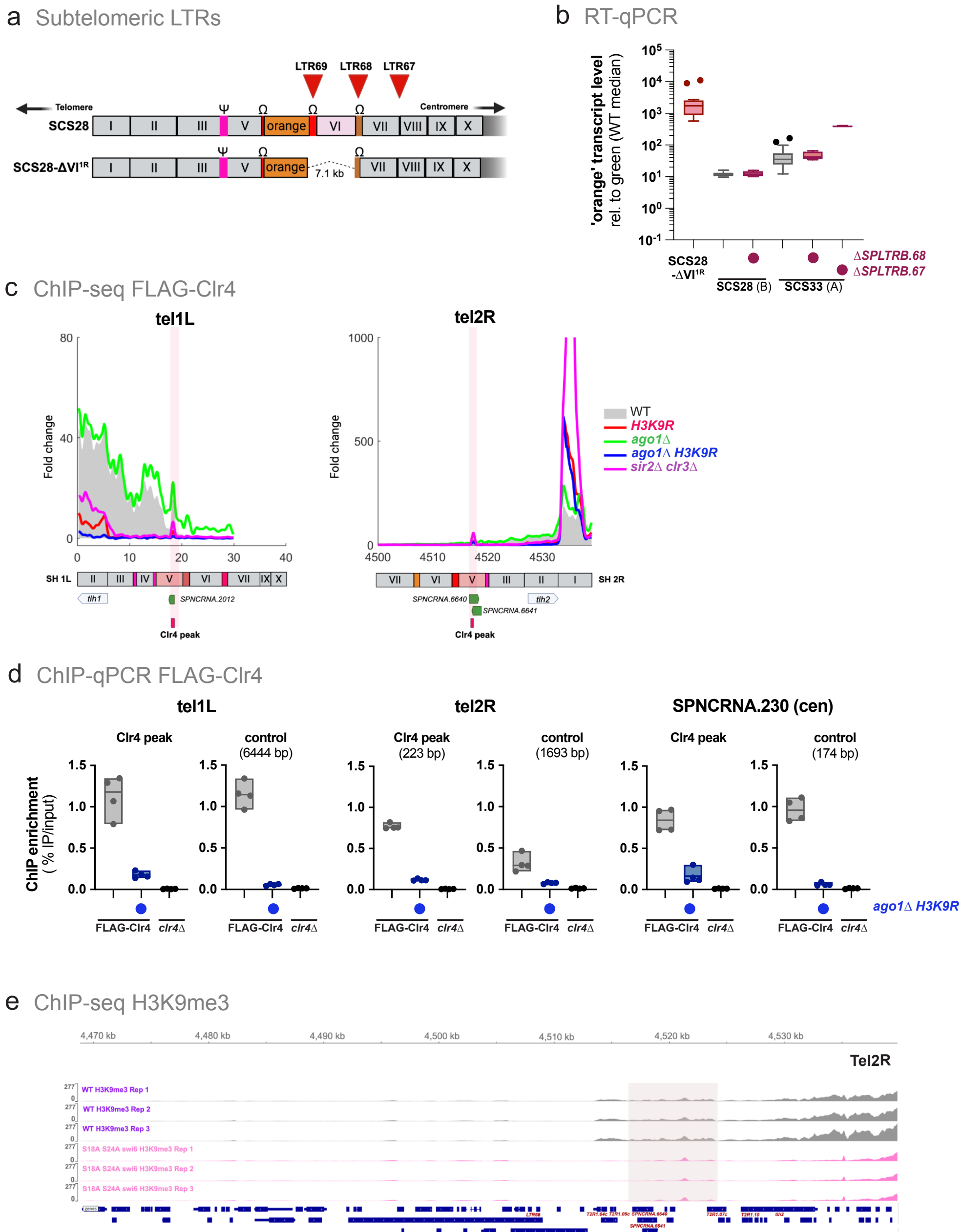

Supplementary Figure S6: Control of SH gene silencing by *cis* elements outside known nucleation sites.

(a) Schematic showing the positions of long terminal repeats (LTRs, red arrows) in SCS28 and SCS28-ΔVI1R. Created in BioRender. Braun, S. (2026) https://BioRender.com/acno2l3.

(b) RT-qPCR analysis of 'orange' reporter transcript levels in SCS28-ΔVI1R, SCS28 and SCS33 strains in the presence and absence of *SPLTRB.68* and *SPLTRB.67*. Transcript levels were normalized to *act1* and are shown relative to the median 'green' reporter expression in WT (log_{10} scale). Independent biological replicates are shown as Tukey box-and-whisker plots (SCS28-ΔVI1R, n = 16; SCS28, n = 10; SCS28 *SPLTRB.68Δ*, n = 4; SCS33, n = 29; SCS33 *SPLTRB.68Δ*, n = 6; and SCS33 *SPLTRB.67Δ*, n = 4). Boxes show the median and interquartile range; whiskers extend to the most extreme values within 1.5× the interquartile range. Data for SCS28-ΔVI1R, SCS28 and SCS33 are identical to those in Figure 2c.

(c) 3xFLAG-Clr4 ChIP-seq profiles at Tel1L (left) and Tel2R (right) in WT and mutant strains. Schematics of SH regions show common sequence blocks (Oizumi et al. 2021), Clr4 peaks and ncRNAs containing potential Mmi1-recognition sites. ChIP-seq data are reproduced from Khanduja et al. 2024 (NCBI GEO submission GSE223202, GSE223203). The Clr4 ChIP signals are shown after subtraction of the 3xFLAG-only control (*clr4Δ::3xFLAG*).

(d) 3xFLAG-Clr4 ChIP-qPCR analysis in WT and the indicated mutant strains. N-terminally tagged Clr4 was expressed from its endogenous locus; the *clr4Δ* control expressed only the triple-FLAG tag. Data are shown as IP/input without additional normalization; independent biological replicates (n = 4) are shown as individual data points with median and range. Clr4 peaks within *Tel1L* and *Tel2R* correspond to those shown in (c); control regions are indicated by their distance from the respective peak (bp).

(e) H3K9me3 ChIP-seq enrichment at Tel2R in wild-type and *swi6-S18A,S24A* strains. Input-normalized ChIP-seq data from Kennedy et al. 2026 (NCBI GEO submission GSE271394) are shown for three biological replicates per genotype (n = 3). Chromosomal coordinates follow PomBase annotation. A light pink box marks H3K9me3 peaks retained in the mutant near *SPBCPT2R1.07c*. Normalized bigWig tracks were visualized using Integrative Genomics Viewer (IGV); grey denotes wild-type, pink denotes the *swi6-S18A,S24A* mutant.

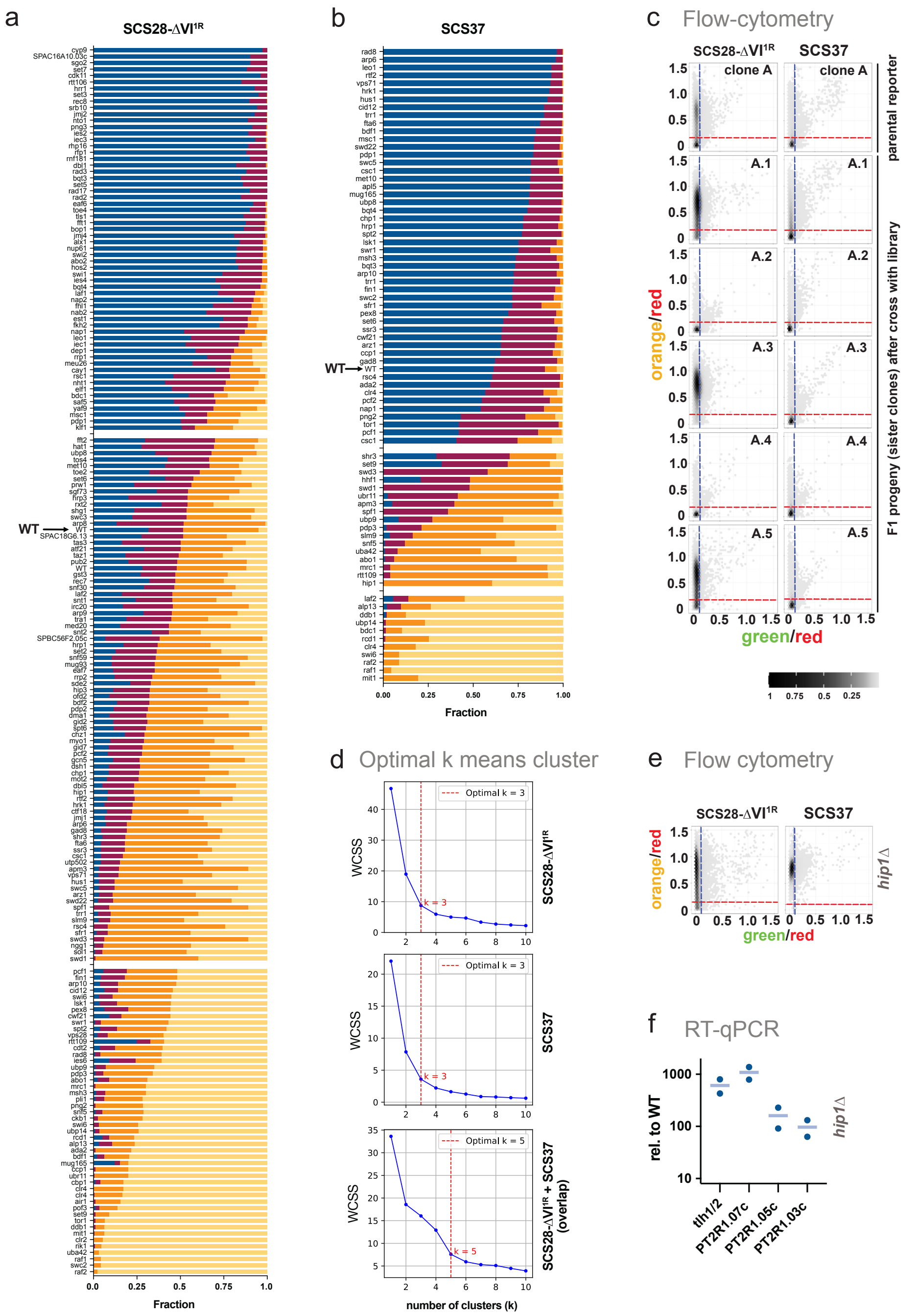

Supplementary Figure S7: Validation of targeted fluorescence-based screen and clustering.

(a, b) Stacked bar plots showing the distribution of cells across four fluorescence expression zones (zones 1–4, increasing 'orange' fluorescence normalized to 'red') in wild-type and mutant strains carrying the SCS28-ΔVI^{1R} (a) or SCS37 (b) reporters. Genes identified in the individual screens were grouped into three clusters based on zone distribution using optimal k-means clustering (k = 3; see d). Within each cluster, horizontal bars represent individual mutants, ordered from top to bottom by increasing fraction of cells in zones 3 and 4. Wild-type is indicated by an arrow.

(c) Effect of heterogeneous subtelomeric arms generated by large-scale genetic crosses of SCS reporter strains with the deletion mutant collection on reporter expression measured by flow cytometry. The top row shows two-dimensional hexbin plots of the parental SCS28-ΔVI^{1R} and SCS37 strains. The panels below show representative F1 progeny from crosses with selected wild-type strains from the mutant collection. F1 progeny show fluorescence distributions similar to the parental reporter strains (Figure 2) and TSA-recovered strains (Figure 7), indicating that heterogeneous subtelomeric arms introduced by the crosses do not measurably affect reporter readout.

(d) Determination of the optimal number of clusters for k-means clustering based on expression profiles from the individual SCS28-ΔVI^{1R} and SCS37 screens and the combined dataset. Panels show the within-cluster sum of squares (WCSS) plotted against the number of clusters (k) using the Elbow method. The optimal number of clusters is indicated by a red dashed line. The top, middle and bottom rows correspond to SCS28-ΔVI^{1R}, SCS37 and the combined dataset, respectively. The optimal number of clusters is k = 3 for the individual screens and k = 5 for the combined dataset.

(e) Flow cytometry analysis of *hip1Δ* in SCS28-ΔVI^{1R} and SCS37. See Figure 5b for plot definitions.

(f) RT-qPCR analysis of selected endogenous SH genes in WT and *hip1Δ* cells in the SCS28 (clone B) strain background. Transcript levels were normalized to *act1* and are shown relative to WT expression. Individual data points represent independent biological replicates (n = 2) from a single experiment.

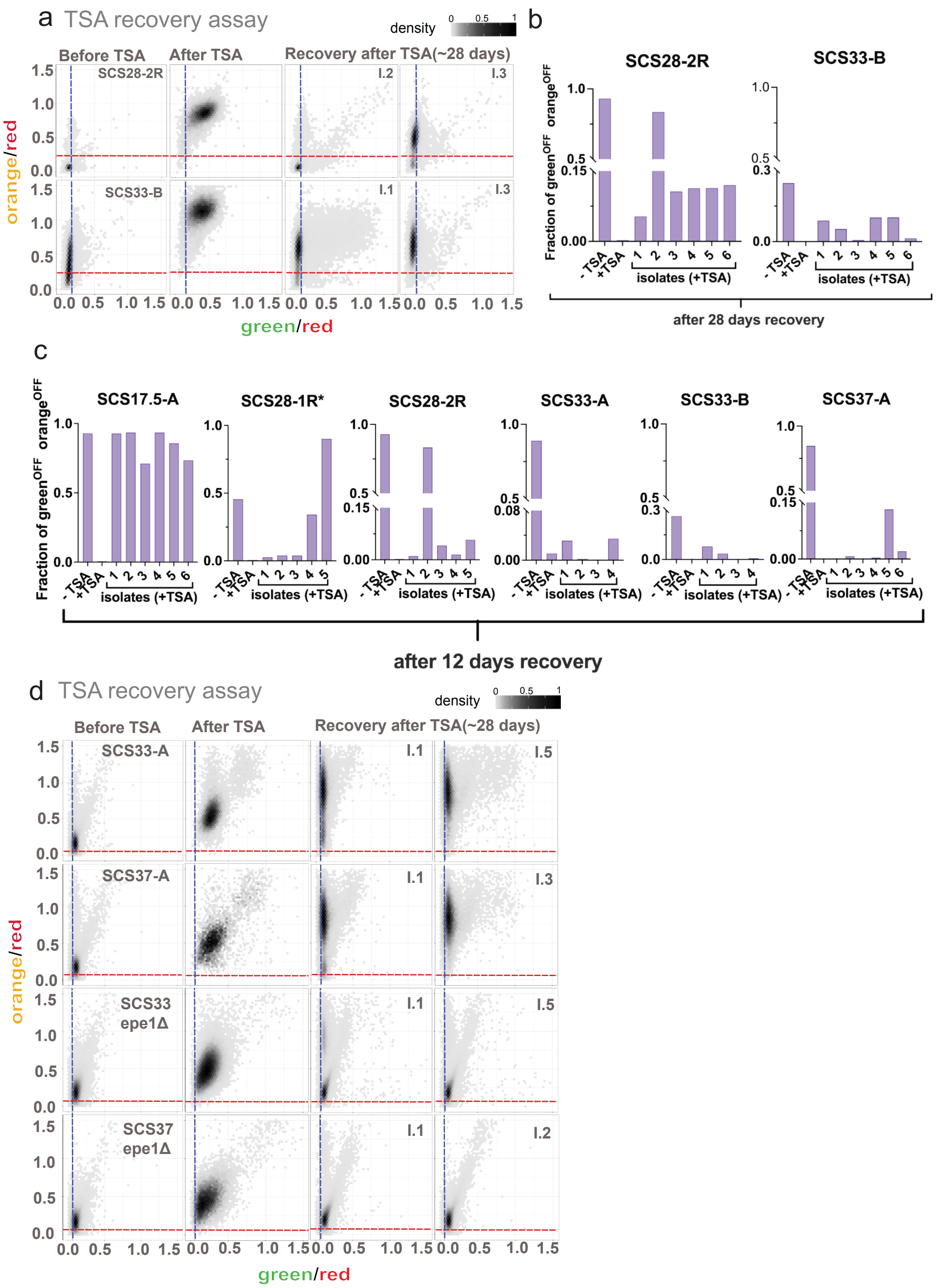

Supplementary Figure S8: Epe1 is required for the apparent fragility of telomere-distal SH heterochromatin.

(a) Two-dimensional hexbin density plots showing 'green' (x-axis) and 'orange' (y-axis) fluorescence normalized to 'red' for SCS reporter constructs. Blue and red dashed lines denote OFF thresholds for 'green' and 'orange' reporters, respectively, as in Figure 2b. Columns show fluorescence profiles in untreated wild-type (WT) cells, following treatment with 30 µM TSA, and in two independent isolates after 28 days of recovery in nutrient-rich medium. Rows correspond to SCS28 and SCS33 constructs (clone B), containing 'orange' reporters at 28 or 33 kb; 'green' and 'red' reporters are located at 11 and 46.5 kb, respectively.

(b, c) Bar plots showing recovery dynamics of SCS reporter clones following TSA treatment. The y-axis represents the fraction of cells in the 'orange'^{OFF}/'green'^{OFF} fluorescence state. Conditions include untreated cells, cells treated with 30 µM TSA, and isolates recovered for 28 days (b) or 12 days (c) in nutrient-rich medium in the indicated reporter strains. Individual bars represent independent post-recovery isolates ($n = 6$ biological replicates per SCS reporter).

(d) Two-dimensional hexbin density plots showing 'green' (x-axis) and 'orange' (y-axis) fluorescence normalized to 'red'. Blue and red dashed lines denote OFF thresholds for 'green' and 'orange' reporters, respectively, as in Figure 7b. Columns show fluorescence profiles in untreated cells, following treatment with 60 µM trichostatin A (TSA), and in two independent isolates after 28 days of recovery in nutrient-rich medium. Rows correspond to SCS33 and SCS37 (clone A), containing 'orange' reporters at 33 and 37 kb, respectively, and the corresponding *epe1Δ* mutants. 'Green' and 'red' reporters are located at 11 and 46.5 kb, respectively.

References:

Ekwall, K., Cranston, G. & Allshire, R. C. Fission yeast mutants that alleviate transcriptional silencing in centromeric flanking repeats and disrupt chromosome segregation. *Genetics* **153**, 1153–69 (1999).

Kennedy, D. R. *et al.* Phosphorylation of HP1/Swi6 relieves competition with Suv39/Clr4 on nucleosomes and enables H3K9 trimethyl spreading. *Nucleic Acids Res.* (2026) **53**(22):gkaf1244. doi:10.1093/nar/gkaf1244.

Khanduja, J. S. *et al.* RNA quality control factors nucleate Clr4/SUV39H and trigger constitutive heterochromatin assembly. *Cell* **187**, 3262-3283.e23 (2024).

Oizumi, Y. *et al.* Complete sequences of Schizosaccharomyces pombe subtelomeres reveal multiple patterns of genome variation. *Nat. Commun.* **12**, 611 (2021).
