# Supplementary material for: Discrete subdomains establish epigenetic diversity in subtelomeric heterochromatin in fission yeast
Source: Nat Commun. 2026 Oct 12;17:11564. doi: 10.1038/s41467-026-78445-5 (PMC13748586; doi:10.1038/s41467-026-78445-5)
Supplement: Supplementary file 2 [file 41467_2026_78445_MOESM2_ESM.pdf]

Description of Additional Supplementary Files

Supplementary Data 1. Fission yeast strains used in this study.

Supplementary Data 2. Plasmids used in this study.

Supplementary Data 3. Primers used in this study.

Supplementary Data 4. Mutants shared between the SCS28-A (truncated reporter strain) and the SCS37-A flow-cytometry-based screen.

Supplementary Data 5. Mutants analyzed in the SCS28-A (truncated reporter strain) flow-cytometry-based screen.

Supplementary Data 6. Mutants analyzed in the SCS37-A flow-cytometry-based screen.
