# Supplementary material for: Discrete subdomains establish epigenetic diversity in subtelomeric heterochromatin in fission yeast
Source: Nat Commun. 2026 Oct 12;17:11564. doi: 10.1038/s41467-026-78445-5 (PMC13748586; doi:10.1038/s41467-026-78445-5)
Supplement: Supplementary file 3 [file 41467_2026_78445_MOESM4_ESM.pdf]

nature portfolio

Corresponding author(s): Bassem Al-Sady, Sigurd Braun

Last updated by author(s): Sep 7, 2023

Reporting Summary

Nature Portfolio wishes to improve the reproducibility of the work that we publish. This form provides structure for consistency and transparency in reporting. For further information on Nature Portfolio policies, see our Editorial Policies and the Editorial Policy Checklist.

Statistics

For all statistical analyses, confirm that the following items are present in the figure legend, table legend, main text, or Methods section.

n/a	Confirmed	
☐	☒	The exact sample size (*n*) for each experimental group/condition, given as a discrete number and unit of measurement
☐	☒	A statement on whether measurements were taken from distinct samples or whether the same sample was measured repeatedly
☒	☐	The statistical test(s) used AND whether they are one- or two-sided<br>*Only common tests should be described solely by name; describe more complex techniques in the Methods section.*
☒	☐	A description of all covariates tested
☒	☐	A description of any assumptions or corrections, such as tests of normality and adjustment for multiple comparisons
☐	☒	A full description of the statistical parameters including central tendency (e.g. means) or other basic estimates (e.g. regression coefficient) AND variation (e.g. standard deviation) or associated estimates of uncertainty (e.g. confidence intervals)
☒	☐	For null hypothesis testing, the test statistic (e.g. *F*, *t*, *r*) with confidence intervals, effect sizes, degrees of freedom and *P* value noted<br>*Give P values as exact values whenever suitable.*
☒	☐	For Bayesian analysis, information on the choice of priors and Markov chain Monte Carlo settings
☒	☐	For hierarchical and complex designs, identification of the appropriate level for tests and full reporting of outcomes
☒	☐	Estimates of effect sizes (e.g. Cohen's *d*, Pearson's *r*), indicating how they were calculated

Our web collection on statistics for biologists contains articles on many of the points above.

Software and code

Policy information about availability of computer code

Data collection	Flow cytometry data were analyzed with BD FACS Diva software
Data analysis	R scripts used for the flow cytometry analysis, have been deposited on Zenodo:https://doi.org/10.5281/zenodo.21360361. Optimal numbers of clusters (k) for k-mean clustering was determined with Python, with, data handling performed using NumPy v2.2.6 and pandas v2.3.0. Within-cluster sum of squares (WCSS) and silhouette scores were calculated across a range of k values with scikit-learn v1.6.1 and visualized using matplotlib v3.10.3. The KneeLocator method from kneed v0.8.5 was used to identify the optimal k according to the elbow method.

For manuscripts utilizing custom algorithms or software that are central to the research but not yet described in published literature, software must be made available to editors and reviewers. We strongly encourage code deposition in a community repository (e.g. GitHub). See the Nature Portfolio guidelines for submitting code & software for further information.

Data

Policy information about availability of data

All manuscripts must include a data availability statement. This statement should provide the following information, where applicable:

- Accession codes, unique identifiers, or web links for publicly available datasets
- A description of any restrictions on data availability
- For clinical datasets or third party data, please ensure that the statement adheres to our policy

Source data are provided with this manusript. The Flow cytometry raw data generated in this study have been deposited on Zenodo under accession number 21360361 [https://doi.org/10.5281/zenodo.21360361]. The processed flow cytometry data are also available on Zenodo. The RT PCR and ChIP data generated in

this study are provided in the Source Data File. All S. pombe strains generated for and used in this study are listed in Table S1 and are available upon request.

Research involving human participants, their data, or biological material

Policy information about studies with human participants or human data. See also policy information about sex, gender (identity/presentation), and sexual orientation and race, ethnicity and racism.

Reporting on sex and gender	n/a
Reporting on race, ethnicity, or other socially relevant groupings	n/a
Population characteristics	n/a
Recruitment	n/a
Ethics oversight	n/a

Note that full information on the approval of the study protocol must also be provided in the manuscript.

Field-specific reporting

Please select the one below that is the best fit for your research. If you are not sure, read the appropriate sections before making your selection.

[x] Life sciences [ ] Behavioural & social sciences [ ] Ecological, evolutionary & environmental sciences

For a reference copy of the document with all sections, see nature.com/documents/nr-reporting-summary-flat.pdf

Life sciences study design

All studies must disclose on these points even when the disclosure is negative.

Sample size	No statistical methods were performed to predetermine the sample size, but the number of biological replicates for each experiment was based on similar studies (Barrales et al., 2016, PMID: 26744419; Ding et al., 2019, PMID: 31811152; Egan et al., 2014, PMID: 24713849; Shichino et al., 2018, PMID: 29424342; Sugiyama et al., 2016, PMID: 26942678; Thillainadesan et al., 2020, PMID: 32415063; Wei et al., 2021, PMID: 33574613).
Data exclusions	No data were excluded from the analysis unless they were classified as outlier using ROUT test (Q = 1%)
Replication	For most experiments, 3-4 (at least 2) independent biological replicates were performed and each replicate was reliably reproduced. For the analysis of RT-qPCR experiments from multiple rounds, up to n = ~25 independent replicates were included. Throughout our study, we were able to replicate all findings reported in this manuscript.
Randomization	For all experiments (Flow cytometry, ChIP-qPCR, RT-qPCR), yeast cultures were grown under the same conditions and collected randomly without bias.
Blinding	Blinding strategies are not relevant in the context of the experiments presented.

Reporting for specific materials, systems and methods

We require information from authors about some types of materials, experimental systems and methods used in many studies. Here, indicate whether each material, system or method listed is relevant to your study. If you are not sure if a list item applies to your research, read the appropriate section before selecting a response.

Materials & experimental systems

n/a	Involved in the study	
[ ]	[x]	Antibodies
[x]	[ ]	Eukaryotic cell lines
[x]	[ ]	Palaeontology and archaeology
[x]	[ ]	Animals and other organisms
[x]	[ ]	Clinical data
[x]	[ ]	Dual use research of concern
[x]	[ ]	Plants

Methods

n/a	Involved in the study	
[x]	[ ]	ChIP-seq
[ ]	[x]	Flow cytometry
[x]	[ ]	MRI-based neuroimaging

Antibodies

Antibodies used	H3K9me2 (Abcam ab1222, Lot 1009758-4 and -6), H3K9me3 (Active Motif AM39161, Lot: 0102204 and 22355218-11), H3K14ac (Abcam ab52946, Lot: GR149741-17), H4K16ac (Active Motif 39167, Lot: 08719903), H3K9me3 (AM39062, Lot: 23342141-14 for sorted ChIP), H3 pan-acetyl (AM39040, Lot: 28518008 for sorted ChIP)
Validation	All antibodies used are commercially available (Abcam, ActiveMotif), and their use in ChIP experiments were described and validated by the manufacturers.

Plants

Seed stocks	n/a
Novel plant genotypes	n/a
Authentication	n/a

Flow Cytometry

Plots

Confirm that:

- [x] The axis labels state the marker and fluorochrome used (e.g. CD4-FITC).
- [x] The axis scales are clearly visible. Include numbers along axes only for bottom left plot of group (a 'group' is an analysis of identical markers).
- [ ] All plots are contour plots with outliers or pseudocolor plots.
- [x] A numerical value for number of cells or percentage (with statistics) is provided.

Methodology

Sample preparation	Fission yeast cells were grown in rich media at 32 degrees C and directly measured for flow in the same (YE) yeast media.
Instrument	Fortessa X-50 instrument (BD), equipped with a high-throughput sampler (HTS) module
Software	Scripts for flow cytometry analysis have been deposited on Zenodo :https://doi.org/10.5281/zenodo.21360361. We use a custom R script not using immunology style contour plots but rather 2D density hexbin plots as we have published prior (Greenstein et al 2018, 2020, 2022, Murawska et al 2021, Challal et al 2023).
Cell population abundance	Here, weare not using classical immunology flow to examine cell populations. We are examining instead isogenic fission yeast strains in each well. We are determining the distribution of cells with respect to their green and orange reporters, as normalized to a red constant reporter. This strategy was extensively validated in Greenstein et al 2018.
Gating strategy	In our flow cytometry analysis (all flow plots except Figure 2F) cells were not gated; instead, the entire population was analysed, as described in the Methods and detailed in the flow analysis code deposited on Zenodo. Therefore, no gating strategy was employed. For the sorting experiment shown in 2f, the gating strategy is provided directly in the figure.

- [ ] Tick this box to confirm that a figure exemplifying the gating strategy is provided in the Supplementary Information.
