# Supplementary material for: Discrete subdomains establish epigenetic diversity in subtelomeric heterochromatin in fission yeast
Source: Nat Commun. 2026 Oct 12;17:11564. doi: 10.1038/s41467-026-78445-5 (PMC13748586; doi:10.1038/s41467-026-78445-5)
Supplement: Supplementary file 4 [file 41467_2026_78445_MOESM5_ESM.pdf]

nature portfolio

Peer Review File

Discrete Subdomains Establish Epigenetic Diversity in Subtelomeric Heterochromatin in Fission Yeast

Corresponding Author: Professor Sigurd Braun

This file contains all reviewer reports in order by version, followed by all author rebuttals in order by version.

Version 0:

Reviewer comments:

Reviewer #1

(Remarks to the Author)
In this manuscript the authors set out to examine in detail how the extensive domains of heterochromatin near telomeres are organised and regulated in the fission yeast Schizosaccharomyces pombe.

Because the subtelomeric regions contain DNA sequence elements that are shared between between multiple telomeres the authors used a previously described strain that retained only one natural subtelomeric region - tel2R (Right telomere chromosome 2; Tashiro et al. 2017).

The authors' main finding is that subtelomeric heterochromatin does not consist of a simple gradient of heterochromatin characterised by strong silencing adjacent to the telomere and silencing declining with distance from the end of the chromosome. Instead, they demonstrate that subtelomeric regions have distinct subdomains in which specific elements influence gene expression/silencing in different regions. A key component of this study is the use of single cell fluorescent reporters inserted at various distances from the telomere to monitor the frequency of expression/silencing. They go on to use these reporters with a collection of ~200 candidate gene deletions to identify factors that specifically alter the proportion of cells expressing/silencing reporters in two different subdomains within subtelomeric heterochromatin. In addition, they combine two types of heterochromatin re-establishment assays with their fluorescent reporters to determine the dynamics of heterochromatin re-establishment following its disruption by 1. treatment with the HDAC inhibitor Trichostatin A or 2. clr4 gene deletion and reintroduction. Heterochromatin was re-established rapidly within the 17.5 kb region from telomere that includes the siRNA target tlh2. However, in both re-establishment assays, when using the orange fluorescent (OFP) reporters 28/33/37 kb from the telomere, individual cells displayed greater variability in expression/silencing. Interestingly, once OFP reporter silencing was disrupted 33 kb (and 37 kb with TSA) from the telomere its silencing was rarely fully re-established during the time of the experiment (3-4 weeks). In general, the expression of endogenous genes in its vicinity also remained high. Thus, once subtelomeric genes are turned on, their expressed state remains relatively stable through multiple cell divisions.

Overall, this is a comprehensive study that uses both bulk and single cell assays to interrogate the composition and regulation of subtelomeric heterochromatin and nearby genes.

Points to consider:

1. The authors found that silencing of subtelomeric genes in SD4(tel2R), their single SH subtelomeric domain strain, is stronger than that of FY1193 where all SH domains are intact. Could the removal of all other subtelomeric SH domains strengthen silencing of this single SH domain by increasing the availability of heterochromatin factors?
Does it appear to be weaker because the SH domain genes assayed are present in multiple copies in FY1193? Has the RT-qPCR data been normalised for the gene copy number e.g. by qPCR on gDNA for these strains?

2. The authors test whether the deletion of genes encoding proteins that are known to affect telomeric silencing affect the OFP reporter inserted 17.5 kb, 28 kb, 33 kb, or 37 kb from the 2R telomere. They use cells deleted for the RNAi component Tas3, the shelterin component Ccq1, the inner nuclear membrane protein Lem2, or the HDAC Clr3. The overall conclusion is that Tas3/RNAi has some effect near the telomere (17.5 kb OFP reporter) whereas Lem2 loss affects silencing further away from the telomere (33 kb and 37 kb OFP reporters) while loss of Ccq1 affects silencing robustness at all reporters.

Deletion of the Clr3/HDAC completely disrupts silencing of all reporters regardless of their location relative to the telomere.

Can the authors provide clearer explanations for these differing effects. Why does Tas3 loss only have a modest effect near the telomere whereas Lem2 loss results in increased expression of all OFP reporters?
It is unclear why the authors chose to delete the tas3 gene rather than ago1 which would remove the better-known direct effector of RNAi. Does ago1D have the same impact on reporter gene and SH gene expression as tas3D?

3. On page 6 the authors introduce a tangential observation exploring how a 7.1 kb region with SH, which is absent in some natural SH domains, and was lost in the OFP 28 kb clone A, but not clone B, reporter. Loss of this 7.1 kb region leads to weaker OFP 28 kb reporter silencing in clone A relative to clone B. This implies that 7.1 kb region contains a secondary heterochromatin nucleation site. The authors strongly argue that Clr4 may be recruited by several Mmi1 recruitment sites that they identify in ncRNAs expressed from this part of the SH subtelomeric region, however, this was not directly tested.
Is the small Clr4 signal detected (Supp Fig. 4) lost if the Mmi1 binding sites are deleted? In the absence of any direct test of this hypothesis it would perhaps be better not to include this. Its inclusion makes the text a bit cumbersome.

4. In the screen, SCS strains crossed with existing knockout collection, which have natural telomeres. Although the HYG marker could be used to select the SCS telomere, the other telomeres would be randomly segregated. Could the variable amount of subtelomeric regions (heterochromatin) have influenced any phenotypes? Were ‘clean’ knockouts of any genes made in the SCS backgrounds directly to check?

5. In the RT-qPCR and ChIP experiments, values are compared to a variety of controls. There seems to be no explanation as to why comparisons were sometimes made with dg and sometimes with act1, sometimes %IP or median SU values, EC (not defined) for instance. This could be confusing, for non expert readers - some explanation should be provided in the methods or legends.

6. SD4[2R+] and a strain with all its telomeres intact (FY1193) are compared to look for any differences in profile of SU heterochromatin at tel2R. However, there did not appear to be a comparison of heterochromatin (or transcript levels) between SD4[2R+] and any SCS strain that contains FP markers driven by the ade6 promoter. The ade6+ gene is relatively highly expressed (~10 molecules per cell; PomBase and references therein); this may be much higher than endogenous genes in the SH and SU regions even in the absence of Clr4. Do the authors have any data on this? Is it possible that inserting a relatively strong promoter affects the levels of heterochromatin assembled in its vicinity? In particular, could it impact the re-establishment of heterochromatin in some sub-domains, particularly on the ade6promoter-driven FP marker gene itself? After treatments such as TSA silencing is not reimposed on e.g. SCS33. Have the authors checked the levels of heterochromatin on nearby genes/regions to test if heterochromatin was re-establish on non-marker genes, or performed re-establishment assays in a strain without marker genes?
Related: did the authors notice any recombination between these direct repeats (ie ade6pro-GFP and ade6pro-OFP that would result in loss of the OFP gene), especially in cells lacking heterochromatin?

7. Note that Ekwall et al 1998 (Cell 91:1021-32) initially used TSA to disrupt and monitor the re-establishment of centromeric silencing.

8. Figure 1. Why switch from the natural left to right tel2R orientation in top panel of Fig. 1a. to a potentially confusing right to left map in the lower panel and histograms in 1b? It would make more sense for all display items to keep the same left to right towards tel2R. It would also be useful to have kb scale for histograms so that it is more obvious how far each gene/PCR product is from the telomere.

9. Page 5: The authors use of term suppression to mean reduced or decreased gene expression. This could cause confusion because suppression has a specific meaning in genetic analyses.

Typing errors:
Line 202: repeat of ‘a role’
Line 297: Other.

(Remarks on code availability)

Reviewer #2

(Remarks to the Author)
The authors report distinct regulation of heterochromatic formation at the sub-telomeric region in fission yeast, using insertion of the fluorescent marker cassettes. expression of fluorescent marker were monitored at single cell level in various genetic mutant background and also after recovery from heterochromatic destruction using TSA or heat-shock treatments. They claimed that the re-establishment of the heterochromatin status is distinct by the region, behaving differently among 11-17kb thl2-proximal region, 28kb regions and 33-37kb regions.
Experiments were well-planned and performed with the controls. While I agree to the authors claim, I am not fully convinced by the conclusion or have not fully understood their logics. The heterochromatin re-establish experiment using fluorescent markers is great, but the authors have not explained the reason of selecting 28 days for recovery nor tested various time periods. Original model was simply spreading of Clr4 from the thl2 locus toward internal region. Clearly, this spread can be

antagonised by Epe1. possible explanation is that heterochromatin spread is slow process, needing time or the number of DNA replications - simply 28 days was not enough to expand toward 28 or 33kb region. Possibility of another heterochromatin establishing loci, which is distinct from the thl2-RITS, is not tested neither. For example, the authors could test by removing 5-15 kb DNA between thl1 and 28kb region to exclude simply distance issue.
Abstract is not clear and vague. The authors should rephrase the findings with concise and clearer words to describe new molecular mechanisms that contributes to the subdomains of the subtelomeric region.
Introduction part is informative but focused only to fission yeast. It lacks global relevance and generalised model across the species. Choice of the fission yeast as a model system is not stated. Indeed, the title may need to add 'in fission yeast' at end.

(Remarks on code availability)

Reviewer #3

(Remarks to the Author)
In this study Mazumder et al have performed an in-depth analysis of S. pombe subtelomeric heterochromatin using a series of strains with fluorescent reporters located at varying distances from the telomere. Using these reporters, they demonstrate that subtelomeric heterochromatin is partitioned into discrete subdomains that have distinct dependencies and epigenetic behaviours. This is an extensive and technically impressive study that provides a highly detailed view of subtelomeric heterochromatin. The data are of high quality and are appropriately presented and analysed. The findings will be of significance to those working on S. pombe heterochromatin. The broader (and long term) impact of the study is a little harder to predict and will depend upon “whether similar fragmentation of subtelomeric heterochromatin occurs in other eukaryotes”.

Specific points
1. I do have some issue with the statement (lines 65-67) “Unexpectedly, we find evidence for multiple, epigenetically distinct subdomains within the SH region that initiate heterochromatin independently of previously described, canonical molecular mechanisms.” Is the finding of distinct subdomains really unexpected? Given the structure of the subtelomere with numerous genes, each with the potential to influence local chromatin structure, it perhaps would have been more surprising to find a homogenous structure. The S. pombe mat locus has previously been shown to have multiple mechanisms for heterochromatin establishment (RNAi/cenH, REII/Clr5 and REIII/Atf) and genes with different silencing dependencies. The composition and organization of Drosophila heterochromatin has also been found to be heterogeneous and dynamic (Swenson et al 2016 Elife).
2. Could the authors expand on the finding of high levels of H3K14ac and H3K9me at SCS28*. Presumably this a result of the bimodal cell population, (i.e. an H3K13ac subpopulation and an H3K14ac sub population? If so why are HK9me3 levels not significantly reduced overall at this locus? Is the HP1 level at this locus reduced relative to 28?
3. Notably, the authors find that loss of atf1 reduces silencing at SCS28 and SCS37. Perhaps the possibility that Atf1 is directing Clr4 and heterochromatin assembly in a similar manner to the mat locus could/should be discussed.
4. Paragraph (lines 300-308). The proteins/complexes detailed in this paragraph lack appropriate references (compare to the complexes detailed in the following paragraph). I am confused by the statement. “This shared dependence on H2A.Z, HIRA, and Snf5, along with the overlapping
requirement for silencing at both reporter loci and equivalent euchromatic insertions, suggests that SH regions and ectopic heterochromatin share chromatin features not typically associated with constitutive heterochromatin.” This statement needs clarification as it seems to suggest that HIRA has no role in constitutive heterochromatin in pombe which is not the case (e.g. Greenall et al 2006; Yamane et al 2001). Tsukii et al., (2022) have also shown that H2AZ plays roles in the maintenance of heterochromatin.
5. The re-establishment of silencing of the various reporters is examined after TSA treatment and heat stress. Have the authors also examined whether other stresses (e.g. nitrogen starvation, osmotic & oxidative stress) lead to a loss of silencing?

Minor points/typos
Fig 1a. It may be worth indicating the presence of the rDNA at Chrm IIIR that effectively replace the subtelomeric sequences at this locus. The grey text on this figure is not legible
Fig 1e. The ‘key’ to the bar chart is missing appropriate labels (i.e. WT and clr4Δ)
Fig 2e is described in the text before Fig 2d
Fig 3a. The blue and red dashed lines indicating the off thresholds are barely visible even when the figure is enlarged on a screen. Generally, the text labels are very small and very difficult to read. For example, much of the text in Fig2e is impossible to read on a standard size print copy. Please increase font size on all Figs where possible
Fig 4. In the text labels there doesn’t seem much point to abbreviating histone chaperone to “histone chaperon.”
Line 481 Please correct typo “S’CS28-1R*”
Line 621. S. pombe (italicize)
Line 631 clr4Δ (italicize)

(Remarks on code availability)

Version 1:

Reviewer comments:

Reviewer #1

(Remarks to the Author)
In this revised manuscript the authors have addressed most of the issues raised in my review of the original manuscript. They have normalised the RT-qPCR data with respect to gene copy number and clarified the normalisation of their ChIP data. They have also provided further mechanistic explanations for how the different pathways affect reporter silencing. They have now included gene knockout experiments in strains where the reporter constructs are absent.

(Remarks on code availability)

Reviewer #2

(Remarks to the Author)
The revised manuscript has significantly improved readability and more clearly articulates the key message. All of the concerns I previously raised have been adequately addressed. I fully support the publication of this manuscript in Nature Communications.

(Remarks on code availability)

Reviewer #3

(Remarks to the Author)
The authors have comprehensively addressed all points raised by this reviewer. They have made substantial revisions to the text and incorporated additional data addressing the chromatin state of the ON/OFF populations (Fig. 2f). Collectively, these changes have significantly strengthened the data and the presentation of the manuscript.

(Remarks on code availability)

Open Access This Peer Review File is licensed under a Creative Commons Attribution 4.0 International License, which permits use, sharing, adaptation, distribution and reproduction in any medium or format, as long as you give appropriate credit to the original author(s) and the source, provide a link to the Creative Commons license, and indicate if changes were made.
In cases where reviewers are anonymous, credit should be given to 'Anonymous Referee' and the source.
The images or other third party material in this Peer Review File are included in the article's Creative Commons license, unless indicated otherwise in a credit line to the material. If material is not included in the article's Creative Commons license and your intended use is not permitted by statutory regulation or exceeds the permitted use, you will need to obtain

permission directly from the copyright holder.
To view a copy of this license, visit https://creativecommons.org/licenses/by/4.0/

Point-by-Point Response to Reviewers' Comments (Mazumder et al.)

We thank all the reviewers for their thoughtful feedback. The reviewers raised a number of points which we have comprehensively addressed in this revised manuscript. We believe that by ensuring that we thoroughly addressed all major points, even if this required some time, this manuscript was significantly strengthened in the process.

Here is the summary of the major additions in this revision:

1. **Direct test of the distance-versus-local regulation model.** In addition to exploiting the naturally occurring deletion of subtelomeric block VI (now renamed SCS28-ΔVI^{1R}), we generated a deletion of the neighboring block V and analyzed both deletions alone and in combination with *tas3Δ* and *taz1Δ*. These experiments argue against a simple distance-dependent spreading model and instead show that the common blocks act as local cis-regulatory elements that shape subtelomeric silencing.
2. **Chromatin state of ON/OFF populations.** To better understand the bimodal behavior of SCS28-ΔVI^{1R}, we flow-sorted ON and OFF populations and performed ChIP–qPCR. While H3K9me3 decreases only modestly in ON cells, H3 acetylation increases dramatically and correlates much more closely with the expression state, providing mechanistic insight into the bistable phenotype.
3. **Expanded analysis of TSA recovery.** We repeated the TSA experiments and substantially extended the analysis by RT–qPCR of endogenous subtelomeric genes. The endogenous genes largely mirror the behavior of the reporters, strengthening our conclusions on position-specific silencing dynamics and recovery.
4. **Independent validation of key mutants.** We generated independent knockouts of several mutants of interest directly in the reporter strains. The phenotypes closely match those obtained from genetic crosses, addressing concerns about mixed subtelomeric chromosome arms. In addition to *tas3*Δ, we now also analyzed *ago1*Δ to independently probe the role of the RITS complex.
5. **Re-evaluation of the chromatin mutant library.** We systematically re-examined all mutants from the targeted mini-screen by barcode sequencing and, where necessary, ORF PCR to identify misannotated strains. This led to the removal or correction of incorrectly annotated mutants (including the previously assigned *atf1*Δ strain), substantially increasing the robustness of the genetic screen. We also included the validation results in the supplementary strain table to document these misannotations for future users of the Bioneer deletion collection.
6. **Improved text and figure presentation.** Throughout the manuscript, we revised the text—particularly the Introduction in response to reviewer comments—and improved the presentation of the figures and results.

Reviewer #1

In this manuscript the authors set out to examine in detail how the extensive domains of heterochromatin near telomeres are organised and regulated in the fission yeast Schizosaccharomyces pombe.

Because the subtelomeric regions contain DNA sequence elements that are shared between multiple telomeres the authors used a previously described strain that retained only one natural subtelomeric region - tel2R (Right telomere chromosome 2; Tashiro et al. 2017).

The authors' main finding is that subtelomeric heterochromatin does not consist of a simple gradient of heterochromatin characterised by strong silencing adjacent to the telomere and silencing declining with distance from the end of the chromosome. Instead, they demonstrate that subtelomeric regions have distinct subdomains in which specific elements influence gene expression/silencing in different regions. A key component of this study is the use of single cell fluorescent reporters inserted at various distances from the telomere to monitor the frequency of expression/silencing. They go on to use these reporters with a collection of ~200 candidate gene deletions to identify factors that specifically alter the proportion of cells expressing/silencing reporters in two different subdomains within subtelomeric heterochromatin. In addition, they combine two types of heterochromatin re-establishment assays with their fluorescent reporters to determine the dynamics of heterochromatin re-establishment following its disruption by 1. treatment with the HDAC inhibitor Trichostatin A or 2. clr4 gene deletion and reintroduction. Heterochromatin was re-established rapidly within the 17.5 kb region from telomere that includes the siRNA target tlh2. However, in both re-establishment assays, when using the orange fluorescent (OFP) reporters 28/33/37 kb from the telomere, individual cells displayed greater variability in expression/silencing. Interestingly, once OFP reporter silencing was disrupted 33 kb (and 37 kb with TSA) from the telomere its silencing was rarely fully re-established during the time of the experiment (3-4 weeks). In general, the expression of endogenous genes in its vicinity also remained high. Thus, once subtelomeric genes are turned on, their expressed state remains relatively stable through multiple cell divisions.

Overall, this is a comprehensive study that uses both bulk and single cell assays to interrogate the composition and regulation of subtelomeric heterochromatin and nearby genes.

Points to consider:

1. The authors found that silencing of subtelomeric genes in SD4(tel2R), their single SH subtelomeric domain strain, is stronger than that of FY1193 where all SH domains are intact.
Could the removal of all other subtelomeric SH domains strengthen silencing of this single SH domain by increasing the availability of heterochromatin factors? Does it appear to be weaker because the SH domain genes assayed are present in multiple copies in FY1193? Has the RT-qPCR data been normalised for the gene copy number e.g. by qPCR on gDNA for these strains?

Response:

We appreciate the reviewer's attention to the quantitative differences of subtelomeric gene silencing in different strain backgrounds, which are intriguing. Measurements in FY1193 reflect an average across all subtelomeric arms due to high sequence similarity and consequent cross-annealing of qPCR primers. As the reviewer correctly anticipated, we used strain-specific genomic DNA for input normalization (i.e., copy numbers are internally accounted for each qPCR), thereby excluding the possibility that these differences arise from gene copy number artefacts (this clarification has now been added to the revised **Figure legend** corresponding to **Suppl. Figure S1c**).

At present, we cannot definitively distinguish whether the comparatively higher expression levels in FY1193 reflect redistribution of silencing factors in SD4, for example through increased availability at a single SH domain, or instead arise from heterogeneity across individual arms in FY1193 potentially driven by structural variation, as we reported for chromosomal arm tel1R. It's noteworthy that Tashiro et al. 2017 (PMID: 21211723) demonstrated that H3K9me persists at subtelomeric sequences even in the absence of SH sequences, likely due to continued recruitment of Clr4 by shelterin bound to telomeric repeats; however, that study did not assess potential redistribution. Thus, multiple, non-mutually exclusive mechanisms may underlie the observed differences in absolute silencing levels. However, these quantitative differences remain modest, and the overall profiles of both strain backgrounds are comparable, supporting our conclusions that the silencing patterns observed in SD4 broadly recapitulates those in FY1193 and are therefore likely to be relevant to other strain backgrounds.

2. The authors test whether the deletion of genes encoding proteins that are known to affect telomeric silencing affect the OFP reporter inserted 17.5 kb, 28 kb, 33 kb, or 37 kb from the 2R telomere. They use cells deleted for the RNAi component Tas3, the shelterin component Ccq1, the inner nuclear membrane protein Lem2, or the HDAC Clr3. The overall conclusion is that Tas3/RNAi has some effect near the telomere (17.5 kb OFP reporter) whereas Lem2 loss affects silencing further away from the telomere (33 kb and 37 kb OFP reporters) while loss of Ccq1 affects silencing robustness at all reporters. Deletion of the Clr3/HDAC completely disrupts silencing of all reporters regardless of their location relative to the telomere.
Can the authors provide clearer explanations for these differing effects. Why does Tas3 loss only have a modest effect near the telomere whereas Lem2 loss results in increased expression of all OFP reporters?
It is unclear why the authors chose to delete the tas3 gene rather than ago1 which would remove the better-known direct effector of RNAi. Does ago1D have the same impact on reporter gene and SH gene expression as tas3D?

Response: We appreciate the request for a clearer mechanistic explanation of pathway-specific contributions and justification of mutant choice. Clr3 and Lem2 exhibit broad effects across subtelomeric domains because Clr3 functions within the SHREC complex, while Lem2 regulates the balance between SHREC and the anti-silencing factor Epe1; these opposing activities collectively determine heterochromatin integrity (Barrales et al 2016; PMID: 2674441). In contrast, shelterin and RNAi act more locally at defined nucleation sites: shelterin is recruited via Taz1 to telomeric repeat motifs and adjacent subtelomeric regions, whereas RITS targets transcripts derived from the cenH-like element within the *tlh2* gene*.* Accordingly, RITS-dependent silencing effects are modest at subtelomeres, because RNAi acts redundantly with other nucleation pathways and appears to play a more prominent role in heterochromatin establishment than in its maintenance (Hansen et al. 2005, PMID: 1563206; Barrales et al. 2016, PMID: 26744419). This framework explains why loss of Tas3 produces only modest and specifically telomere-proximal effects, whereas loss of Lem2 leads to increased expression across all reporters. We have clarified this point in the Results section (**Lines 205-212**).
Regarding mutant choice, Tas3 was selected because it functions as a scaffold within RITS, bridging Argonaute (Ago1) and the chromodomain protein Chp1, thereby specifically disrupting RITS integrity. To address the reviewer's concern, we additionally deleted *ago1*. Both, *ago1Δ* and *tas3Δ,* exhibit highly similar effects on reporter and subtelomeric gene expression, demonstrating that disruption of either component comparably impairs RNAi-mediated silencing (**Suppl. Figure 4c**; **Lines 225-228**).

3. On page 6 the authors introduce a tangential observation exploring how a 7.1 kb region with SH, which is absent in some natural SH domains, and was lost in the OFP 28 kb clone A, but not clone

B, reporter. Loss of this 7.1 kb region leads to weaker OFP 28 kb reporter silencing in clone A relative to clone B. This implies that 7.1 kb region contains a secondary heterochromatin nucleation site. The authors strongly argue that Clr4 may be recruited by several Mmi1 recruitment sites that they identify in ncRNAs expressed from this part of the SH subtelomeric region, however, this was not directly tested.
Is the small Clr4 signal detected (Supp Fig. 4) lost if the Mmi1 binding sites are deleted? In the absence of any direct test of this hypothesis it would perhaps be better not to include this. Its inclusion makes the text a bit cumbersome.

Response: We thank the reviewer for this point. Indeed, our data indicate that the 7.1-kb fragment is required for local silencing of adjacent genes. This fragment overlaps with the common sequences (block VI) on the subtelomeric arms and its deletion represents a natural structural variant. We now also examine the impact of the loss of this fragment and the neighboring block V on local gene expression in more detail in **Figure 4a and 4b** (**Lines 246-278**). Our primary evidence suggesting additional nucleation sites derives from **(1)** recruitment of Clr4 in a manner that is independent of RITS and H3K9 methylation (in an *ago1Δ H3K9R* background; **Suppl. Figure S6c and S6d**; Khanduja et al., 2024,PMID: 38815580); and **(2)** the near coincidence of an H3K9me3 peak that persists in a Swi6 spreading-deficient mutant (**Suppl. Figure S6e**; Kennedy et al, 2025, PMID: 41385327). However, we agree with the reviewer that for the purposes of this manuscript, elaborating the precise mechanism of recruitment at this locus is beyond the scope, and partial elaboration hinders the manuscript flow. Therefore, we have removed references to the Mmi1 pathway. Instead, we use these observations as evidence consistent with cryptic nucleation sites in this telomere-distal region, without further discussing the underlying mechanism.

4. In the screen, SCS strains crossed with existing knockout collection, which have natural telomeres. Although the HYG marker could be used to select the SCS telomere, the other telomeres would be randomly segregated. Could the variable amount of subtelomeric regions (heterochromatin) have influenced any phenotypes? Were 'clean' knockouts of any genes made in the SCS backgrounds directly to check?

Response: The reviewer is correct that progeny from genetic crosses between SCS reporters and the knockout library contain heterogeneous subtelomeric chromosomal arms (i.e. subtelomeres with and without SH sequences). Because the library also contains wild-type control strains, we performed flow cytometry analysis on selected wild-type sibling F1 progeny derived from these crosses. We did not detect any differences in reporter expression compared to the parental reporter strain (shown in **Suppl. Figure S7c**), indicating that heterogeneous subtelomeric arms do not measurably affect the reporter readout (**Lines 325-328**). In addition, we generated independent knockouts of selected candidates in several reporter backgrounds, as well as in the original SD4 strain, which contains the single tel2R SH region but lacks the reporter constructs, and performed RT-qPCR analyses. Results for representative prominent mutants are presented in **Figure 5e** and **Suppl. Figure S7f** of the revised manuscript. Importantly, chromatin-dependent effects of *swd1* (Set1C complex) and *bdc1* (NuA4 complex) are also observed in the absence of reporter constructs, indicating that original phenotype detected by flow cytometry after the cross is recapitulated in independent KOs and is not dependent on the reporter context (**Lines 367–369** and **379–383**).

5. In the RT-qPCR and ChIP experiments, values are compared to a variety of controls. There seems to be no explanation as to why comparisons were sometimes made with dg and sometimes with act1, sometimes %IP or median SU values, EC (not defined) for instance. This could be confusing, for non expert readers - some explanation should be provided in the methods or legends.

Response: We agree that normalization varied among figures in the previous version and apologize that the rationale was not clearly explained. In the revised manuscript, we have thoroughly re-analyzed all ChIP and RT-qPCR data and sought to present them in a more coherent manner.

For bulk ChIP experiments assessing heterochromatin modifications (e.g. H3K9me2/me3), we applied the following strategy: we normalized ChIP signals first to the corresponding input DNA and subsequently to the averaged pool of subtelomeric loci on the same subtelomeric arm. This approach provides the most robust and consistent results, allowing us to detect even subtle differences compared with normalization to a single internal locus (e.g. act1/EC, euchromatin) on different regions or chromosomes or heterochromatin loci (e.g. pericentromeric *dg* repeats). Details of the pool-normalization procedure for each graph are provided in the accompanying **Source Data** file. The newly analyzed data are shown in **Figures 1b-d** and **2e**; for comparison, we also show input-normalized data in **Suppl. Figure S2c**. This heterochromatin-based normalization cannot be applied when the reference heterochromatin itself is altered, for example in *ago1Δ* or *H3K9R* strains used to measure Clr4 recruitment. For this reason, we presented FLAG-Clr4 binding in **Suppl. Figure S6c** and **S6d** as input-normalized ChIP data without additional normalization. For ChIP following flow cytometry sorting (**Figure 2f**), cell numbers were limited. Hence we opted for a more limited range of normalization loci, as described in Greenstein et al 2018. Specifically, we normalized H3K9me3 at subtelomeres to the levels at pericentromeric dh repeats, whereas H3 acetylation levels were normalized to the euchromatic act1 locus.

For RT-qPCR experiments, we initially used normalization against either act1 or the median value of subtelomere unique (SU) genes downstream of the SH region; however, normalization to SU-region genes provides a more robust internal reference, likely because it is based on multiple genes from the same chromosomal arm, we have applied this approach in most figures. For visualization, endogenous subtelomeric transcript levels are shown relative to the median WT value of *tlh2* (or the 'green' reporter in SCS strains), allowing direct comparison of expression levels across loci and experiments. As the reviewer suggested, we have added brief explanations to the figure legends describing the rationale for the normalization strategy.

6. SD4[2R+] and a strain with all its telomeres intact (FY1193) are compared to look for any differences in profile of SU heterochromatin at tel2R. However, there did not appear to be a comparison of heterochromatin (or transcript levels) between SD4[2R+] and any SCS strain that contains FP markers driven by the ade6 promoter.
The ade6+ gene is relatively highly expressed (~10 molecules per cell; PomBase and references therein); this may be much higher than endogenous genes in the SH and SU regions even in the absence of Clr4.
Do the authors have any data on this?
Is it possible that inserting a relatively strong promoter affects the levels of heterochromatin assembled in its vicinity?
In particular, could it impact the re-establishment of heterochromatin in some sub-domains, particularly on the ade6promoter-driven FP marker gene itself**?**

Response: The reviewer raises an important point that reporter genes are generally expressed at higher levels than native heterochromatin transcripts. When we developed the three-color HSS sensor (Al-Sady et al., 2016, PMID: 27479698; Greenstein et al., 2018, PMID: 30020075) our objective was to identify a reporter that provides a broad dynamic range in fluorescence (~10-fold between *clr4*Δ and wild type) at heterochromatic loci. Because *ura4* yields only a ~4-fold dynamic range, we selected *ade6*, which met this criterion and, like *ura4*, has been extensively validated as a heterochromatin reporter (Allshire et al. 1994, PMID: 8287474; Allshire et al. 1995, PMID: 7851795; Grewal and Klar 1996, PMID: 8689692).

Although reporter insertion may influence the local chromatin environment to some extent, we do not find evidence that it weakens silencing of the subtelomeric domain. On the contrary, the presence of the reporters appears to sensitize the system, resulting in stronger repression, particularly at telomere-proximal genes. This is illustrated in **Suppl. Figure S4e**, which compares wild-type, *tas3*Δ and *taz1*Δ strains in the SCS17.5 reporter and the parental SD4 background. The mechanism underlying this increased repression remains unknown.
To directly address the reviewer's concern, we additionally compared reporter and endogenous gene expression following TSA treatment over a ~4-week recovery period using RT–qPCR (**new Figure 7d**; **Lines 445-450**). The reporters closely mirrored the recovery kinetics of adjacent endogenous genes, indicating that they faithfully report the local epigenetic state and its re-establishment after perturbation. Similarly, in our previous study (Greenstein et al. 2018, PMID: 30020075), reporters inserted at the ΔK MAT locus reproduced the heterochromatin establishment and maintenance behaviors described previously for reporter-free strains. Together, these findings suggest that although reporter insertions sensitize subtelomeric silencing, they faithfully capture the regional dynamics of heterochromatin establishment or recovery.

After treatments such as TSA silencing is not reimposed on e.g. SCS33. Have the authors checked the levels of heterochromatin on nearby genes/regions to test if heterochromatin was re-establish on non-marker genes, or performed re-establishment assays in a strain without marker genes?

Response: We addressed this question by repeating TSA treatment experiments for the SCS33 and SCS37 reporter strains by quantifying transcriptional repression at both reporter loci and adjacent endogenous regions (new data, **Figure 7d**). Cells were treated with TSA, and samples were taken for RT-qPCR analysis immediately after treatment (day 0), as well as after partial and prolonged recovery (day 10 and day 25), alongside untreated controls. We observed that recovery kinetics at the reporter loci closely mirrored those of neighboring genes, thereby directly assessing silencing re-establishment beyond the marker constructs. Specifically, telomere-proximal regions (green reporter, tlh2, T2R1.07) exhibited intermediate recovery, whereas telomere-distal regions (orange reporters SCS33 and SCS37, and genes T2R1.04c, T2R1.02, B2B2.19) showed very slow or incomplete re-establishment of silencing. In contrast, pericentromeric repeats fully re-established silencing by day 10 (**Lines 445-450**). We further observed variability among independent isolates at telomere-proximal genes, consistent with previous reports that heterochromatin establishment is stochastic (Obersriebnig et al., 2016, PMID: 27167753) and consistent with our results following Clr4 re-introduction (**Figure 7f; Lines 474-48**3). Together, these results demonstrate that heterochromatin re-establishment after TSA treatment is recapitulated at endogenous subtelomeric regions and is not restricted to reporter constructs.

Related: did the authors notice any recombination between these direct repeats (ie ade6pro-GFP and ade6pro-OFP that would result in loss of the OFP gene), especially in cells lacking heterochromatin?

Response: We appreciate this point by the reviewer. We have now extensively (Greenstein et al 2018, 2020, PMID: 30020075&3180552; Murawska 2021, PMID: 34731638; Greenstein and Ng et al 2022, PMID: 35584134; Challal et al 2023, PMID: 37279920; Kennedy et al 2025, MID: 41385327) used the ade6p GFP/OFP system to measure heterochromatin behavior. In *clr4+,* we do not observe measurable loss of reporters. We do occasionally, after very long strain maintenance, observe loss of colors in *Δclr4*. However, in this study, we did not maintain strains for long enough periods to observe significant color loss, even in *clr4Δ.*

7. Note that Ekwall et al 1998 (Cell 91:1021-32) initially used TSA to disrupt and monitor the re-establishment of centromeric silencing.

Response: Thank you for the note. We have included the reference in our manuscript (**Lines 418-419**).

8. Figure 1. Why switch from the natural left to right tel2R orientation in top panel of Fig. 1a. to a potentially confusing right to left map in the lower panel and histograms in 1b? It would make more sense for all display items to keep the same left to right towards tel2R.
It would also be useful to have kb scale for histograms so that it is more obvious how far each gene/PCR product is from the telomere.

Response: We appreciate the reviewer's concern regarding the change in orientation and agree that consistency across panels is generally desirable. However, we chose to maintain the reversed orientation to remain consistent with previous studies (e.g. Oizumi et al. 2021, PMID: 33504776) and because displaying all loci in a common telomere-to-distal (left-to-right) direction facilitates direct visual comparison of heterochromatin across different subtelomeres and chromosomal arms. In this configuration, the gradient of H3K9 methylation from the telomeric end toward more distal regions is consistently represented across panels. Nevertheless, to minimize potential confusion, we revised the figures to ensure clearly indicated orientation (e.g. explicit telomere/distal labels) and explain the reasoning in the **Figure 1 legend.** We also added a kb scale to the histograms, allowing readers to better assess the distance of each locus from the telomere.

9. Page 5: The authors use of term suppression to mean reduced or decreased gene expression. This could cause confusion because suppression has a specific meaning in genetic analyses.

Response: We fully agree with the reviewer and have replaced misleading uses of the term "suppression" throughout the manuscript. We retained the term only where it refers to the suppression of a genetic or molecular phenotype.

Typing errors:
Line 202: repeat of 'a role' Corrected
Line 297: Other. Corrected (i.e. entire sentence was removed during revision).
(Remarks on code availability)

Reviewer #2

The authors report distinct regulation of heterochromatic formation at the sub-telomeric region in fission yeast, using insertion of the fluorescent marker cassettes. expression of fluorescent marker were monitored at single cell level in various genetic mutant background and also after recovery from heterochromatic destruction using TSA or heat-shock treatments. They claimed that the re-establishment of the heterochromatin status is distinct by the region, behaving differently among 11-17kb thl2-proximal region, 28kb regions and 33-37kb regions.
Experiments were well-planned and performed with the controls. While I agree to the authors claim, I am not fully convinced by the conclusion or have not fully understood their logics.
The heterochromatin re-establish experiment using fluorescent markers is great, but the authors have not explained the reason of selecting 28 days for recovery nor tested various time periods.

Response: While we showed recovery after 28 days in the original **Figure 7** (formerly Figure 6), we had also included additional intermediate time points (e.g., 12 days) in the previous manuscript (**Suppl. Figure S8c**; formerly Suppl. Figure S6c) (Lines 425-427). To more systematically address recovery kinetics, we repeated the TSA recovery experiments using RT-qPCR as a quantitative readout and analyzed multiple recovery time points (0, 10, and 25 days; **Figure 7d),** comparing both reporter genes and endogenous subtelomeric genes (see also response to Reviewer 1, point 6). These experiments demonstrate that silencing recovery is highly position-dependent, revealing fast (pericentromeres), intermediate (telomere-proximal genes), and slow or incomplete recovery (telomere-distal regions). Importantly, we observed no further recovery at telomere-distal loci between days 10 and day 25, indicating that silencing re-establishment plateaus within this time window. We therefore conclude that extending the analysis beyond ~25 days is unlikely to alter the conclusion that telomere-distal loci recover substantially more slowly, or incompletely, than telomere-proximal and pericentromeric regions (**Lines 445-450**).

Original model was simply spreading of Clr4 from the thl2 locus toward internal region. Clearly, this spread can be antagonised by Epe1. possible explanation is that heterochromatin spread is slow process, needing time or the number of DNA replications - simply 28 days was not enough to expand toward 28 or 33kb region.

Response: We agree that heterochromatin spreading may be a relatively slow process and cannot formally exclude that silencing could eventually be re-established at the 33 and 37 kb loci over even longer timescales. However, 28 days corresponds to ~300 generations in *S. pombe,* which represents a substantial period for re-establishment of chromatin states and is comparable to timescales considered in theoretical models of epigenetic inheritance experiments (e.g. the t_{gen} parameter; Dodd et al 2007, PMID: 17512413). Moreover, we observed no further recovery between days 10 and 25, indicating that silencing re-establishment reaches a plateau within this time window. While recovery at later times therefore cannot be excluded, our data demonstrate that the kinetics and extent of heterochromatin re-establishment differ substantially between chromosomal regions, which is the central conclusion of these experiments.

Possibility of another heterochromatin establishing loci, which is distinct from the thl2-RITS, is not tested neither. For example, the authors could test by removing 5-15 kb DNA between thl1 and 28kb region to exclude simply distance issue.

Response: We appreciate this suggestion. In the previous version, we have already included a reporter construct lacking a 7.1-kb segment between the 28 and 35 kb positions, corresponding to the common SH block VI. In the revised manuscript, we renamed this strain SCS28-ΔVI^{1R} (formerly

SCS28-1R*) to more accurately reflect that this naturally occurring deletion of lock VI resembles the structure of chromosomal arm tel1R. Notably, shortening the SH region does not increase silencing, as expected under a simple distance-dependent spreading model. Instead, deletion of block VI caused de-repression of both the reporter and endogenous genes downstream of the deletion (new **Figure 4a**), arguing against the idea that silencing is determined primarily by distance from sheltering/RITS nucleation sites. We further extended this analysis by deleting the adjacent SH block V (~22-27 kb). Consistent with the block VI deletion, this manipulation did not enhance downstream silencing but instead weakened silencing, particularly upstream in the telomere-proximal region (~15-22 kb). Similar to the block VI deletion, loss of block V did not enhance silencing but instead caused local derepression, particularly at genes upstream of the deletion in the telomere-proximal region (**Figure 4b**, **Lines 246-264**).

Moreover, this defect resembled that of *tas3Δ*, and combining the mutations resulted in largely non-additive effects (Lines 265-268). We now present the analyses of block VI and block V together in the new **Figure 4**.

Although the underlying mechanism remains unresolved, these findings support the existence of local cis-regulatory elements that influence subtelomeric silencing and are consistent with the presence of an additional cryptic heterochromatin nucleation site near block V, where Clr4 is recruited independently of RNAi and pre-existing H3K9 methylation (**Suppl. Figure S6c,d**; **Lines 294-300**).

Abstract is not clear and vague. The authors should rephrase the findings with concise and clearer words to describe new molecular mechanisms that contributes to the subdomains of the subtelomeric region.

Response: We have revised the abstract to address the reviewer's point.

Introduction part is informative but focused only to fission yeast.
It lacks global relevance and generalised model across the species.
Choice of the fission yeast as a model system is not stated.
Indeed, the title may need to add 'in fission yeast' at end.

Response: We have revised the Introduction to explicitly address the reviewer's concerns regarding scope, general relevance, and model justification. Specifically, we now frame heterochromatin spreading within a broader evolutionary context by highlighting conserved principles across eukaryotes before focusing on *Schizosaccharomyces pombe* as a tractable model system. We also explicitly justify the choice of fission yeast by emphasizing its genetically defined and minimally redundant heterochromatin pathways, which enable mechanistic dissection of spreading phenomena. In addition, we have clarified how insights from this system inform general models of heterochromatin regulation across species. Finally, we have revised the title to include 'fission yeast' to more accurately reflect the study's scope.

Reviewer #3

In this study Mazumder et al have performed an in-depth analysis of S. pombe subtelomeric heterochromatin using a series of strains with fluorescent reporters located at varying distances from the telomere. Using these reporters, they demonstrate that subtelomeric heterochromatin is partitioned into discrete subdomains that have distinct dependencies and epigenetic behaviours. This is an extensive and technically impressive study that provides a highly detailed view of subtelomeric heterochromatin. The data are of high quality and are appropriately presented and analysed. The findings will be of significance to those working on S. pombe heterochromatin. The broader (and long term) impact of the study is a little harder to predict and will depend upon "whether similar fragmentation of subtelomeric heterochromatin occurs in other eukaryotes".

We thank the reviewer for this positive assessment of our work and for recognizing the technical scope of the study, the quality of the data, and the significance of our findings.

Specific points

1. I do have some issue with the statement (lines 65-67) "Unexpectedly, we find evidence for multiple, epigenetically distinct subdomains within the SH region that initiate heterochromatin independently of previously described, canonical molecular mechanisms." Is the finding of distinct subdomains really unexpected? Given the structure of the subtelomere with numerous genes, each with the potential to influence local chromatin structure, it perhaps would have been more surprising to find a homogenous structure. The S. pombe mat locus has previously been shown to have multiple mechanisms for heterochromatin establishment (RNAi/cenH, REII/Clr5 and REIII/Atf) and genes with different silencing dependencies. The composition and organization of Drosophila heterochromatin has also been found to be heterogeneous and dynamic (Swenson et al 2016 Elife).

Response: The reviewer does make a good point here. True homogeneity is rare in any type of chromatin domain once examined more closely. Especially larger heterochromatin domains tend to feature multiple nucleators. We should have been more specific that the surprising result to us are the apparent distinct epigenetic behaviors of these domains, ranging from rapid and robust recovery, to bimodality, and finally extreme fragility of otherwise strongly silenced states at steady state. However, multiple nucleation pathways within the subtelomere have not been clearly defined, and we now refer to this as a novel, rather than necessarily surprising result in this revised version.

2. Could the authors expand on the finding of high levels of H3K14ac and H3K9me at SCS28*. Presumably this a result of the bimodal cell population, (i.e. an H3K13ac subpopulation and an H3K14ac sub population? If so why are HK9me3 levels not significantly reduced overall at this locus? Is the HP1 level at this locus reduced relative to 28?

Response: We interpret this reviewer comment to ask whether elevated H3K14Ac levels in SCS28-ΔVI^{1R} (formerly SCS28* or SCS28-1R*) are the result of differential accumulation in bimodal populations, and the implications for H3K9me3 and HP1 accumulation. To address this question, we performed a Flow Cytometry Assisted Cell Sorting (FACS) experiment followed by ChIP, similar to what we did before in Greenstein et al 2018, PMID: 30020075. We sorted SCS28-ΔVI^{1R} cells into HIGH (green OFF, orange ON) and LOW (green OFF, orange OFF) populations and were able to collect 8x10^6 cells per condition. We then performed ChIP for H3K9me3 and for acetylated H3 using a panH3 acetyl antibody (H3K4/K9/K14/K18/K23/K27), which prominently detects H3K14ac and has previously yielded robust results from bulk populations in our hands. We were able to obtain good data on these low-yield sorted materials. The results show that H3 acetylation is nearly absent in the

LOW state but strongly enriched in the HIGH state, reaching levels comparable to those at the actively transcribed *act1* gene. Generally, H3K9me3 is inversely correlated with H3 acetyl, except that H3K9me3 is not absent in the HIGH state, it is rather reduced compared to the low state (**Figure 2f, Lines 187-194**). These findings are consistent with our bulk ChIP data (**Figure 2e**), which showed only a modest reduction in H3K9me3 in SCS28-ΔVI1R compared with other reporters. Together, these data suggest that increased H3 acetylation does not require complete loss of H3K9me3 and that both chromatin features can coexist to some degree at this subtelomeric locus. We also attempted to examine Swi6/HP1 occupancy by performing FLAG-Swi6 ChIP on FACS-sorted SCS28-ΔVI1R populations. However, the limited number of sorted cells (~8 × 10^6 per population) did not yield sufficient signal above background for reliable quantification. We therefore cannot conclude whether Swi6/HP1 occupancy changes in parallel with H3K9me3.

3. Notably, the authors find that loss of atf1 reduces silencing at SCS28 and SCS37. Perhaps the possibility that Atf1 is directing Clr4 and heterochromatin assembly in a similar manner to the mat locus could/should be discussed.

Response: We thank the reviewer for this suggestion. However, we found that the *atf1*Δ mutant used in the original screen was misannotated in the commercial Bioneer deletion library and in fact corresponds to another gene deletion (*sro7*). Although the strain displayed a silencing defect in the reporter assay, this misannotation means we cannot attribute that phenotype to Atf1. We therefore did not pursue this mutant further and cannot draw conclusions about a direct role for Atf1 in Clr4 recruitment or heterochromatin assembly at SCS28-ΔVI^{1R} and SCS37.
Because misannotated strains are known to occur in the Bioneer deletion collection, we systematically re-examined all mutants shown in **Figure 5c** (former Figure 4c) and the **Supplementary Figures S7a and S7b** (former S5a and S5b). We amplified the deletion cassettes by PCR and sequenced the unique barcodes, as described previously (Muhammad et al. 2024, PMID: 39565189), and compared them with the published Bioneer barcode collection (Han et al. 2010, PMID: 20473289). When the barcode did not match, we additionally tested the corresponding open reading frame deletion by PCR.
Overall, we verified 68/83 (81.9%) mutants shared between SCS28-ΔVI^{1R} and SCS37 screens (Figure 5c; see also **Suppl. Table S4**), 158/197 (80,2%; **Suppl. Table S5**) mutants from the SCS28-ΔVI^{1R} screen, and 68/86 (79%; **Suppl. Table S6**) mutants from the SCS37 screen by barcode sequencing, genomic PCR, and comparison with our previous study (Muhammad et al. 2024). To avoid confusion, we removed misannotated mutants from the revised Figure 5c and Suppl. Figures S7a,b unless the corrected gene encodes a protein with a clear chromatin-associated function, in which case we replaced the original annotation with the correct gene name. We also added the barcode sequencing and ORF PCR results to the previous strain table (Suppl. Table S4-S6) to document these misannotations and alert future users of the version 3 of the Bioneer collection.

4. Paragraph (lines 300-308). The proteins/complexes detailed in this paragraph lack appropriate references (compare to the complexes detailed in the following paragraph). I am confused by the statement. "This shared dependence on H2A.Z, HIRA, and Snf5, along with the overlapping requirement for silencing at both reporter loci and equivalent euchromatic insertions, suggests that SH regions and ectopic heterochromatin share chromatin features not typically associated with constitutive heterochromatin." This statement needs clarification as it seems to suggest that HIRA has no role in constitutive heterochromatin in pombe which is not the case (e.g. Greenall et al 2006; Yamane et al 2001). Tsukii et al., (2022) have also shown that H2AZ plays roles in the maintenance of heterochromatin.

Response: We thank the reviewer for highlighting relevant prior studies and identifying potentially misleading statements regarding the roles of these candidates. We have revised the text to clarify that

factors such as HIRA have well-established roles in constitutive heterochromatin (e.g., Greenall et al., 2006, PMID: 16428807; Yamane et al., 2011, PMID: 21211723), while our previous work indicates that their contributions to spreading in ectopic heterochromatin is distinct (**Lines 566-570**). Accordingly, we have revised the relevant sections in the Results and Discussion (**Lines 366-373** and **566-570**). Regarding H2A.Z: upon systematically re-examining the mutants shown in Figure 5 (formerly Figure 4c), we found that *pht1Δ* (H2A.Z) corresponds to a strain with misannotated barcodes (see also our reply to point 3 by this reviewer). The identity of this strain could not be resolved, as the retrieved barcode sequences did not match the annotated gene deletion. We have therefore removed this mutant from the dataset presented in this figure and revised the corresponding text accordingly. We thank the reviewer for identifying these issues and have incorporated additional references to ensure accurate representation of the literature.

5. The re-establishment of silencing of the various reporters is examined after TSA treatment and heat stress. Have the authors also examined whether other stresses (e.g. nitrogen starvation, osmotic & oxidative stress) lead to a loss of silencing?

Response: We thank the reviewer for this point. TSA treatment, i.e., transient inhibition of HDACs or heat treatment, which injects energy into the system, are two quite different stresses showing broadly similar patterns. We have not examined stresses beyond those two at this point. It would be interesting to examine other stresses *S. pombe* could be experiencing, but we feel this is outside the scope of this manuscript, especially as some of those biotic and abiotic stresses themselves alter heterochromatin states through *S. pombe* native pathways, i.e. nitrogen starvation-triggered release of heterochromatin islands, and would thus require significant additional controls to elude confounders.

Minor points/typos

We thank the reviewer for the careful reading of the manuscript and for identifying these typos and inconsistencies, which all have been addressed in the revised version.

Fig 1a. It may be worth indicating the presence of the rDNA at Chrm IIIR that effectively replace the subtelomeric sequences at this locus. The grey text on this figure is not legible

Response: We have added the position of the rDNA locus to the schematic in Figure 1a and adjusted the font color to improve readability

Fig 1e. The 'key' to the bar chart is missing appropriate labels (i.e. WT and clr4Δ). The missing labels have been added

Fig 2e is described in the text before Fig 2d. The figure order has been corrected, and we have carefully checked the manuscript for consistency and correct figure references throughout.

Fig 3a. The blue and red dashed lines indicating the off thresholds are barely visible even when the figure is enlarged on a screen. Generally, the text labels are very small and very difficult to read. For example, much of the text in Fig2e is impossible to read on a standard size print copy. Please increase font size on all Figs where possible. We increased the thickness of the blue and red threshold lines in Figure 3a (and related figures). In addition, we increased font sizes of axis labels, tick axis labels, and other figure annotations wherever this was possible to improve readability

Fig 4. In the text labels there doesn't seem much point to abbreviating histone chaperone to "histone chaperon." Corrected.

Line 481 Please correct typo "S'CS28-1R*" Corrected. During the revision, we also renamed SCS28-1R to SCS28-ΔVI^{1R} for consistency with strains carrying the adjacent block V deletion.

Line 621. S. pombe (italicize) Corrected.

Line 631 clr4Δ (italicize) Corrected.
